# Supplementary material for: Aminopeptidase N Inhibitors as Pointers for Overcoming Antitumor Treatment Resistance
Source: Int J Mol Sci. 2022 Aug 29;23(17):9813. doi: 10.3390/ijms23179813 (PMC9456425; doi:10.3390/ijms23179813)
Supplement: Supplementary file 1 [file ijms-23-09813-s001.zip › ijms-1859095-supplementary.pdf]

Supplementary Materials S1

„Aminopeptidase N inhibitors as pointers for

Overcoming Antitumor Treatment Resistance”

Oldřich Farsa [1], Veronika Ballayová [1,*], Radka Žáčková [1], Peter Kollar [2], Tereza Kauerová [2] and Peter Zubáč [1]

[1] Department of Chemical Drugs, Faculty of Pharmacy, Masaryk University, Palackého 1946/1, 612 00 Brno, Czech Republic
[2] Department of Pharmacology and Toxicology, Faculty of Pharmacy, Masaryk University, Palackého 1946/1, 612 00 Brno, Czech Republic

Content:

Overview of target compounds with their activities 1
Spectral and other structure identification data of all the prepared compounds 8
Examples of 1D and 2D NMR spectra of the selected compounds as figures 18
Determination of purity of target compounds 66
Solubility issues during inhibitory activity determination and attempts to circumvent them 66
Detailed results of determination of antiproliferative activity of target compounds in different cancer cell lines 66

Overview of target compounds with their activities

**Substance code**	**Structure**	**IC_{50} [µmol/L]**
14-2-1[1]	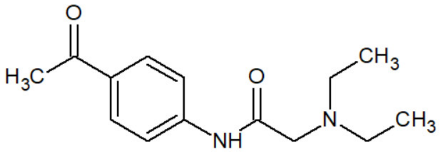	366.0*
14-6-1[1]	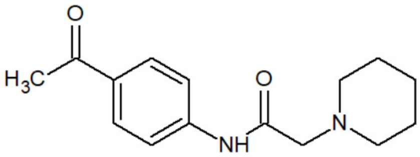	173.0
14-8-1[1]	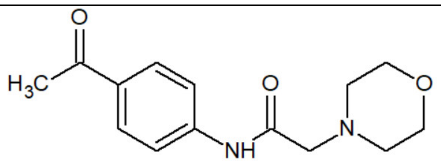	425.4
14-10-1[1]	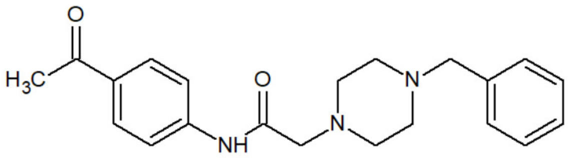	427.3*

22-2-1[2]	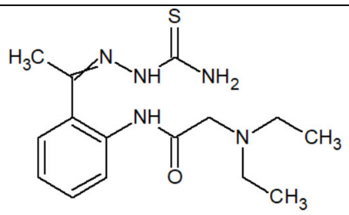	50.4
22-3-1[2]	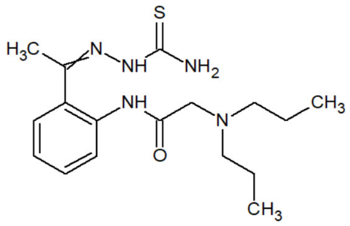	70.7
22-4-1[2]	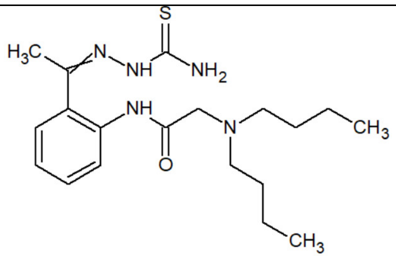	3.4
22-5-1[2]	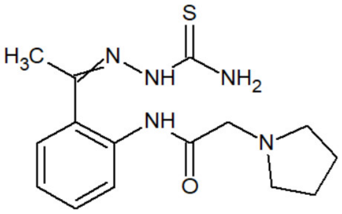	149.2
22-6-1[1]	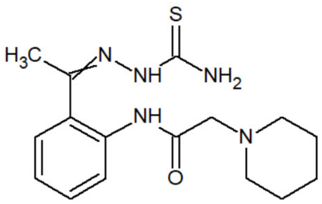	778.0
22-7-1[1]	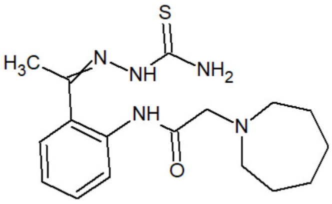	169.3
23-2-1[2]	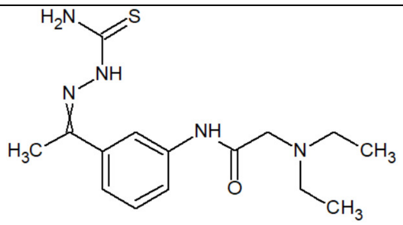	39.5

23-3-1[2]	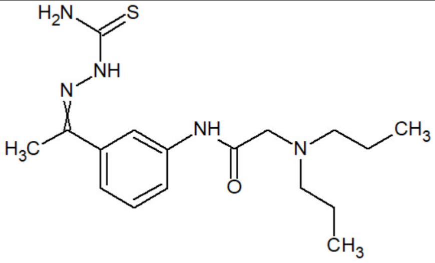	686.5
23-4-1[2]	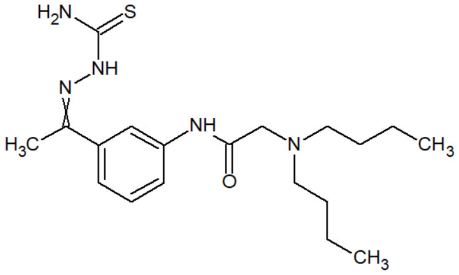	34.6
23-5-1[2]	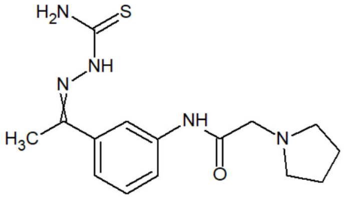	20.26
23-6-1[2]	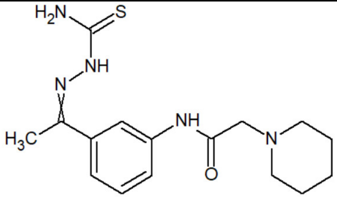	83.1
23-7-3[1]	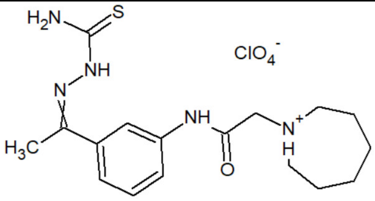	35.6
23-8-1[2]	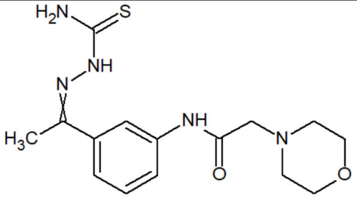	397.7
23-9-1[2]	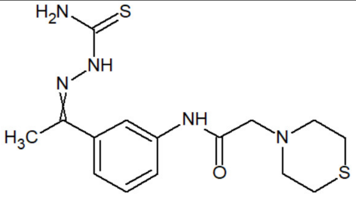	714.7

23-11-1[2]	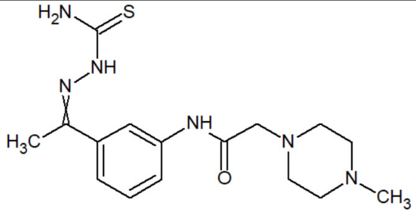	7.86
24-2-2[1]	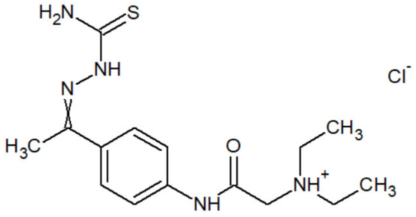	46.4
24-2-3[1]	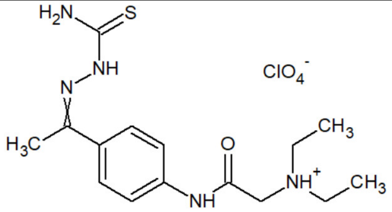	18.9
24-3-1[1]	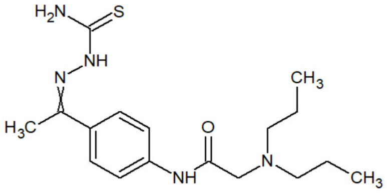	1266
24-4-1[1]	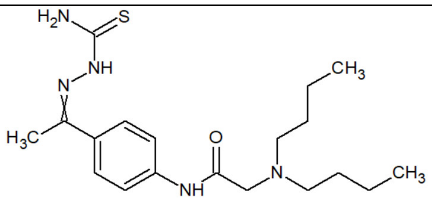	1679
24-5-1[1]	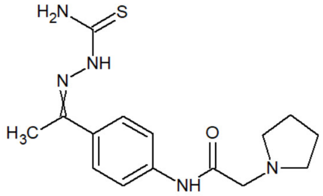	35.6
24-5-3[1]	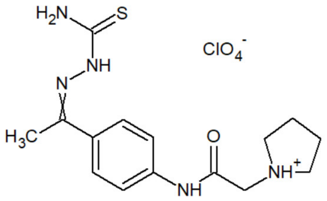	23.5
24-6-1[1]	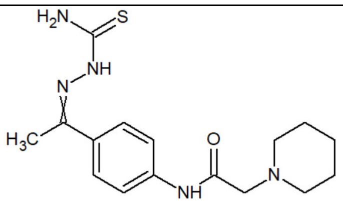	1549

24-6-3[1]	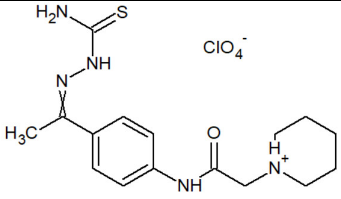	4732**
24-7-1[1]	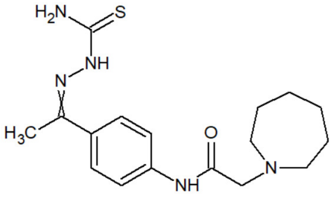	44.1
24-8-1[1]	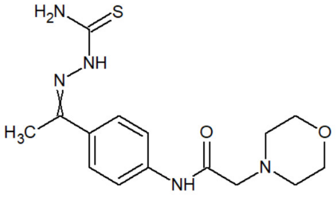	6.79
24-9-1[2]	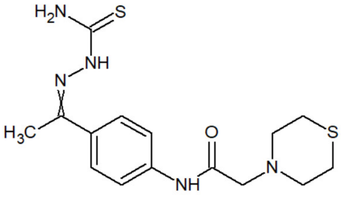	1372
24-10-3[1]	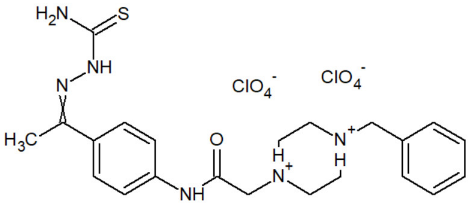	22.3
24-11-2[1]	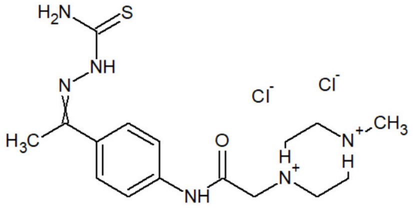	13.3
24-11-3[1]	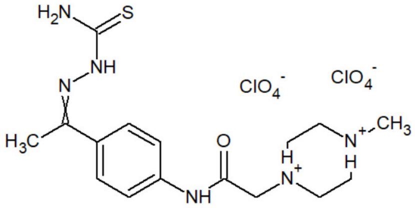	150.1
32-2-1[2]	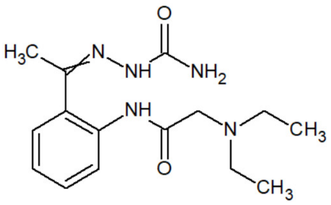	68.3

32-3-1[2]	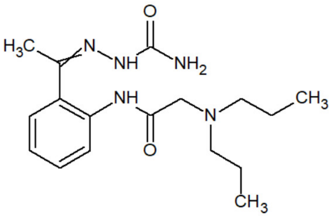	538.1
32-4-1[2]	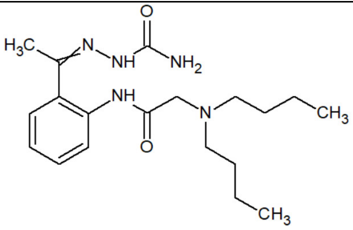	19.5
32-5-1[2]	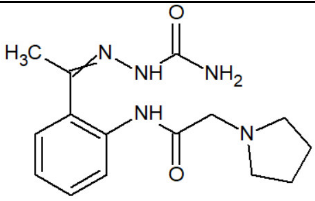	30.0
32-6-1[2]	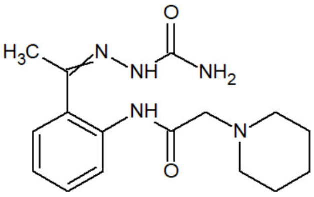	130.5
32-7-1[2]	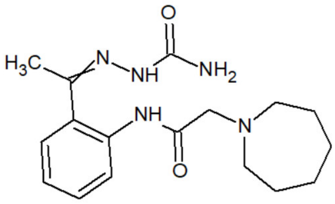	92.4
32-8-1[2]	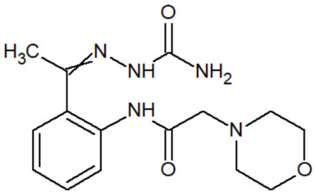	5137
32-11-1[2]	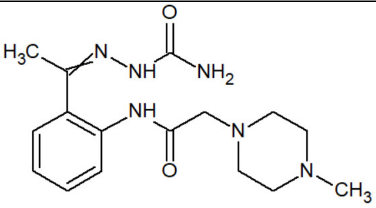	20.6
33-2-1[2]	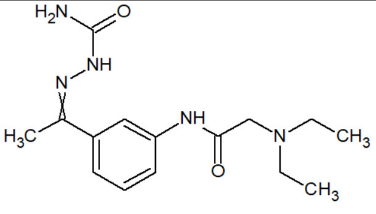	135.1

33-4-1[2]	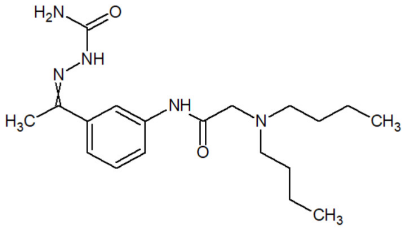	413.7
33-5-1[2]	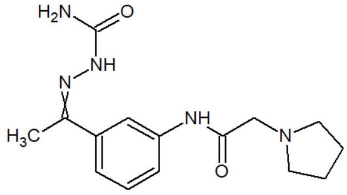	10.4
33-6-1[2]	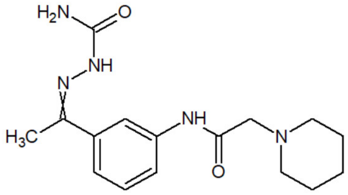	19.7
33-7-1[2]	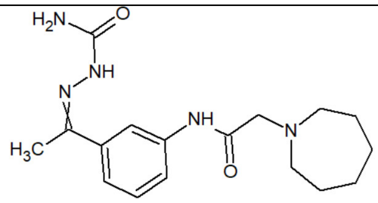	121.0
33-10-1[2]	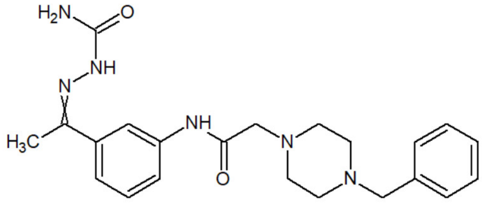	96.8
34-2-1[2]	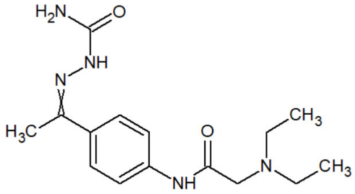	138.0
34-4-1[2]	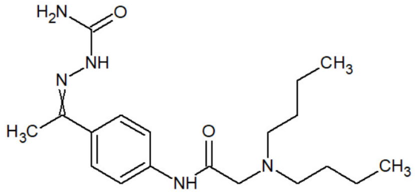	305.3
34-5-1[2]	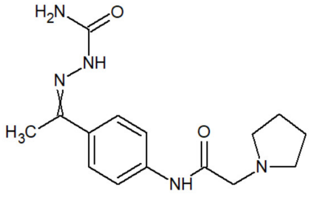	13.6

34-6-1[1]	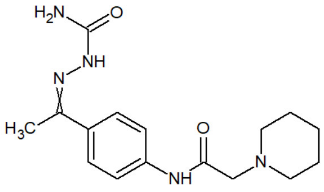	18.4
34-7-1[2]	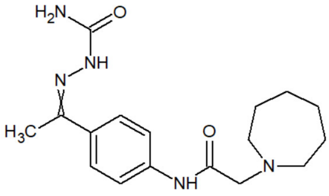	121.9
34-8-1[1]	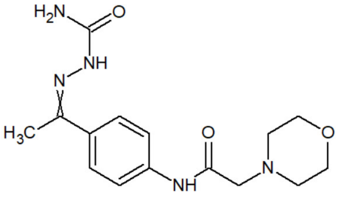	1644
34-9-1[2]	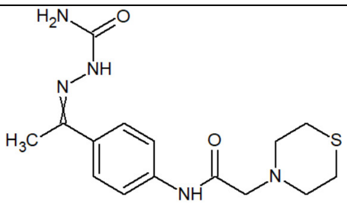	5.9
34-10-1[2]	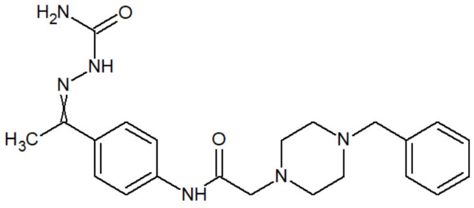	109.12

[1] Synthetized compounds with experimentally determined IC_{50} values

[2] Compounds with predicted IC_{50} values

* Solubility improved by adding DMSO into the buffer solution.

** Solubility improved by adding NMP into the buffer solution.

Spectral and other structure identification data of all the prepared compounds

12-1, 13-1, 14-1

2-(chloroacetamido)acetophenone (12-1). Yield 93%, mp=135.8 – 136.1 °C.
^{1}H-NMR (400 MHz, CHLOROFORM-d), δ [ppm]: 12.48 (s, 1H), 8.74 (d J = 8,8 Hz, 1H), 7.94 (d J = 7,6 Hz, 1H), 7.59 (t J = 8,2 Hz, 1H), 7.19 (t J = 8,2 Hz, 1H), 4.19 (s, 2H), 2.69 (s, 3H)
^{13}C-NMR (101 MHz, CHLOROFORM-d), δ [ppm]: 202.5, 165.8, 139.7, 135.1, 131.6, 123.4, 122,6, 120.8, 43.3, 28.5

3-(chloroacetamido)acetophenone (13-1). Yield 95%, mp=143.6 – 144.2 °C.
^{1}H-NMR (400 MHz, CHLOROFORM-d), δ [ppm]: 8.44 (s, 1H), 8.05 (t J = 4 Hz,1H), 7.91 (s, 1H), 7.75 (t J = 8 Hz, 1H), 7.47 (t J = 8 Hz, 1H), 4.21 (s, 2H), 2.61 (s, 3H)

^{13}C-NMR (101 MHz, CHLOROFORM-d), δ [ppm]: 197.6, 164.1, 137.9, 137.2, 129.5, 125.0, 124.6, 119.6, 42.8, 26.7

4-(chloroacetamido)acetophenone (14-1). Yield 96%, mp=150.6 – 151.2 °C.
^{1}H-NMR (400 MHz, CHLOROFORM-d), δ [ppm]: 8.43 (s, 1H), 7.97 (d J = 8 Hz, 2H), 7.67 (d J = 7,2 Hz, 2H), 4.21 (s, 2H), 2.59 (s, 3H)
^{13}C-NMR (101 MHz, DMSO-d6), δ [ppm]: 196.5, 165.2, 142.7, 132.2, 129.5,118.6, 43.6, 26.5

12-2-1 to 14-14-1

4-[2-(diethylamino)acetamido]acetophenone (14-2-1). Yield 87%, mp=164.7 – 165.0 °C.
^{1}H-NMR (400 MHz, DMSO-d6), δ [ppm]: 9.94 (s, 1H), 7.91 (d J = 8,8 Hz, 2H), 7.79 (d J = 8,8 Hz, 2H), 3.18 (s, 2H), 2.62 – 2.56 (m, 4H), 2.51 (s, 3H), 1.00 (t J = 7,4 Hz, 6H)
^{13}C-NMR (101 MHz, DMSO-d6), δ [ppm]: 196.5, 170.6, 142.8, 129.4, 127.3, 118.6, 57.4, 47.8, 26.5, 11.9

MS (ESI) m/z: 249.20 $[M+H]^+$.

4-[2-(piperidine-1-yl)acetamido]acetophenone (14-6-1). Yield 72,6%, mp=191.3– 191.5 °C.
^{1}H-NMR (400 MHz, DMSO-d6), δ [ppm]: 10.00 (s, 1H), 7.92 (d J = 8,8 Hz, 2H), 7.77 (d J = 8,8 Hz, 2H), 3.09 (s, 2H), 2.51 (s, 3H), 2.45 – 2.43 (m, 4H), 1.56 – 1.52 (m, 4H), 1.41 – 1.38 (m, 2H)
^{13}C-NMR (101 MHz, DMSO-d6), δ [ppm]: 197.0, 169.8, 143.5, 132.3, 129.9, 119.1, 63.2, 54.6, 27.0, 26.0, 24.1

MS (ESI) m/z: 261.22 $[M+H]^+$.

4-[2-(morpholine-4-yl)acetamido]acetophenone (14-8-1). Yield 69,4%, mp=180.6– 181.5 °C.
^{1}H-NMR (400 MHz, DMSO-d6), δ [ppm]: 9.28 (s, 1H), 7.96 (d J = 8,8 Hz, 2H), 7.67 (d J = 8,8 Hz, 2H), 3.79 (t J = 4,4 Hz, 4H), 3.17 (s, 2H), 2.64 (t J = 4,6 Hz, 4H), 2.58 (s, 3H)
^{13}C-NMR (101 MHz, DMSO-d6), δ [ppm]: 196.5, 168.7, 142.9, 131.8, 129.4, 118.7, 66.1, 62.0, 53.1, 26.4

MS (ESI) m/z: 263.19 $[M+H]^+$.

4-[2-(benzylpiperazine-4-yl)acetamido]acetophenone (14-10-1). Yield 70%, mp=206.4 – 207.2 °C.
^{1}H-NMR (400 MHz, DMSO-d6), δ [ppm]: 10.05 (s, 1H), 7.92 (d J = 8 Hz, 2H), 7.77 (d J = 8 Hz, 2H), 7.34 – 7.24 (m, 5H), 3.47 (s, 2H), 3.15 (s, 2H), 2,53 – 2.51 (m, 4H), 2.50 (q J = 1,2 Hz, 4H) 2.43 (s, 3H)
^{13}C-NMR (101 MHz, DMSO-d6), δ [ppm]: 196.6, 169.0, 143.0, 138.2, 131.8, 129.4, 128.8, 128.2, 126.9, 118.6, 62.1, 61.8, 52.8, 52.5, 26.5

MS (ESI) m/z: 352.25 $[M+H]^+$.

22-2-1 to 24-14-1

N-{2-[1-[(carbamothioylamino)imino]ethyl]phenyl}-2-(pyrrolidine-1-yl)acetamide (22-5-1). Yield 49%, mp=197,4 – 198,6 °C.
^{1}H-NMR (400 MHz, DMSO-d6), δ [ppm]: 11.02 (s, 1H), 8.61 (t J = 8,2 Hz, 1H), 8.02 (d J = 6,4 Hz, 1H), 7.56 (t J = 7,2 Hz, 1H), 7.16 (t J = 6,8 Hz, 1H), 4.39 (s, 3H), 3.16 (s, 2H), 3.01 – 2.89 (m, 4H), 2.34 (s, 3H), 1.93 – 1.89 (m, 4H)
^{13}C-NMR (400 MHz, DMSO-d6), δ [ppm]: 191.8, 181.1, 168.9, 139.2, 134.6, 132.1, 122.9, 122.7, 119.8, 56.3, 54.4, 28.8, 22.9
IR (ATR, 4000 – 400 cm-1), ν [cm-1]: 3362, 3260, 3163, 2935, 2858, 2807, 1683, 1664, 1645, 1617, 1573, 1506, 1451, 1434, 1418, 1356, 1334, 1311, 1283, 1240, 1168, 1129, 1112, 1039, 992, 959, 939, 899, 869, 859, 848, 833, 817

MS (ESI) m/z: 320.19 $[M+H]^+$.

N-{2-[1-[(carbamothioylamino)imino]ethyl]phenyl}-2-(piperidine-1-yl)acetamide (22-6-1). Yield 54,7%, mp=205,9 – 206,6 °C;
^{1}H-NMR (400 MHz, DMSO-d6), δ [ppm]: 12.30 (s, 1H), 8.69 (t J = 8,8 Hz, 1H), 8.06 (d J = 6,8 Hz, 1H), 7.58 (t J = 7,2 Hz, 1H), 7.18 (t J = 6,8 Hz, 1H), 4.48 (s, 3H), 3.06 (s, 2H), 2.64 (s, 3H), 2.44 (m, 4H), 1.65 (m, 4H), 1.43 (m, 2H)
^{13}C-NMR (101 MHz, DMSO-d6), δ [ppm]: 201.8, 181.1, 170.4, 139.2, 134.4, 132.2, 122.8, 122.6, 119.8, 63.0, 54.4, 28.8, 25.3, 23.5
IR (ATR, 4000 – 400 cm-1), ν [cm-1]: 3363, 3257, 3162, 2934, 2857, 2807, 1682, 1665, 1643, 1616, 1570, 1505, 1452, 1435, 1417, 1357, 1333, 1311, 1282, 1242, 1168, 1128, 1113, 1038, 994, 961, 949, 898, 871, 859, 847, 830, 814

MS (ESI) m/z: 334.18 $[M+H]^+$.

2-(azepane-1-yl)-N-{2-[1-[(carbamothioylamino)imino]ethyl]phenyl}acetamide (22-7-1). Yield 46%, mp=206.9 – 207.8 °C.
^{1}H-NMR (400 MHz, DMSO-d6), δ [ppm]: 11.15 (s, 1H), 8.66 (t J = 8,4 Hz, 1H), 8.02 (d J = 6,4 Hz, 1H), 7.51 (t J = 7,2 Hz, 1H), 7.16 (t J = 6,4 Hz, 1H), 4.48 (s, 3H), 3.26 (d J = 12 Hz, 2H), 2.63 – 2.59 (m, 4H), 2.54 (s, 3H), 1.53 – 1.46 (m, 8H)
^{13}C-NMR (101 MHz, DMSO-d6), δ [ppm]: 201.1, 181.1, 170.2, 139.2, 133.4, 132.2, 123.3, 122.6, 119.5, 62.1, 54.9, 28.5, 26.3, 25.5
IR (ATR, 4000 – 400 cm-1), ν [cm-1]: 3364, 3258, 3159, 2935, 2857, 2807, 1682, 1669, 1640, 1616, 1570, 1503, 1452, 1437, 1418, 1357, 1335, 1311, 1281, 1245, 1170, 1130, 1115, 1039, 992, 960, 950, 896, 873, 857, 849, 831, 815

MS (ESI) m/z: 348.25 $[M+H]^+$.

N-{3-[1-[(carbamothioylamino)imino]ethyl]phenyl}-2-(diethylamino)acetamide (23-2-1). Yield 75%, mp=262.5 – 263.7 °C.
^{1}H-NMR (400 MHz, DMSO-d6), δ [ppm]: 10.08 (s, 1H), 8.52 (s, 1H), 8.23 (d J = 4 Hz, 1H), 7.84 (t J = 2,4 Hz, 1H), 7.63 (t J = 4,2 Hz, 1H), 7.46 (t J = 4,4 Hz, 1H), 4.38 (s, 2H), 3.16 (s, 2H), 2.68 – 2.61 (m, 7H), 1,37 (t J = 7,6 Hz, 6H)
^{13}C-NMR (101 MHz, DMSO-d6), δ [ppm]: 196.9, 181.1, 169.6, 139.8, 135.1, 129.9, 123.4, 122.6, 118.8, 61.3, 55.9, 28.8, 23.4

IR (ATR, 4000 – 400 cm-1), ν [cm-1]: 3362, 3259, 3164, 2935, 2855, 2810, 1679, 1666, 1644, 1618, 1572, 1505, 1453, 1437, 1415, 1359, 1333, 1312, 1284, 1239, 1167, 1129, 1114, 1041, 995, 963, 949, 900, 873, 861, 849, 832, 816

MS (ESI) m/z: 322.22 $[M+H]^+$.

N-{3-[1-[(carbamothioylamino)imino]ethyl]phenyl}-2-(dipropylamino)acetamide (23-3-1). Yield 72%, mp=213.9 – 214.7 °C.
^{1}H-NMR (400 MHz, DMSO-d6), δ [ppm]: 10.08 (s, 1H), 8.58 (s, 1H), 8.24 (d J = 4,4 Hz, 1H), 7.86 (t J = 2 Hz, 1H), 7.65 (t J = 4,2 Hz, 1H), 7.46 (t J = 4 Hz, 1H), 4.43 (s, 2H), 3.18 (s, 2H), 2.74 – 2.69 (m, 7H), 1.48 – 1.39 (m, 4H), 0,84 (t J = 7,6 Hz, 6H)
^{13}C-NMR (101 MHz, DMSO-d6), δ [ppm]: 196.9, 181.1, 169.6, 139.8, 135.1, 129.9, 123.4, 122.6, 118.8, 61.3, 55.9, 28.8, 25.6, 23.4
IR (ATR, 4000 – 400 cm-1), ν [cm-1]: 3357, 3273, 3170, 2957, 2934, 2873, 2824, 1677, 1668, 1645, 1600, 1510, 1498, 1466, 1407, 1361, 1337, 1316, 1273, 1209, 1180, 1158, 1122, 1112, 1077

MS (ESI) m/z: 350.25 $[M+H]^+$.

N-{3-[1-[(carbamothioylamino)imino]ethyl]phenyl}-2-(pyrrolidine-1-yl)acetamide (23-5-1). Yield 53%, mp=215.6 – 216.4 °C.
^{1}H-NMR (400 MHz, DMSO-d6), δ [ppm]: 10.16 (s, 1H), 8.20 (d J = 4 Hz ,1H), 7.84 (t J = 2,1 Hz, 1H), 7.63 (t J = 4,2 Hz, 1H), 7.43 (t J = 4 Hz, 1H), 4.43 (s, 3H), 3.18 (s, 2H), 2.94 – 2.89 (m, 4H), 2.55 (s, 3H), 1.88 – 1.82 (m, 4H)
^{13}C-NMR (101 MHz, DMSO-d6), δ [ppm]: 196.9, 181.1, 169.0, 139.1, 135.3, 129.9, 123.8, 122.4, 118.8, 62.1, 54.4, 27.8, 22.6,
IR (ATR, 4000 – 400 cm-1), ν [cm-1]: 3359, 3270, 3167, 2956, 2935, 2870, 2820, 1677, 1666, 1640, 1604, 1519, 1494, 1472, 1403, 1360, 1338, 1316, 1275, 1209, 1177, 1161, 1118, 1113, 1075

MS (ESI) m/z: 320.19 $[M+H]^+$.

N-{3-[1-[(carbamothioylamino)imino]ethyl]phenyl}-2-(piperidine-1-yl)acetamide (23-6-1). Yield 58,9%, mp=227.6 – 227.9 °C;
^{1}H-NMR (400 MHz, DMSO-d6), δ [ppm]: 9.87 (s, 1H), 8.20 (d J = 4 Hz ,1H), 7.88 (t J = 2 Hz, 1H), 7.65 (t J = 4 Hz, 1H), 7.46 (t J = 4 Hz, 1H), 4.48 (s, 3H), 3.08 (s, 2H), 2.55 (s, 3H), 2.44 – 2.41 (m, 4H), 1.58 – 1.52 (m, 4H), 1.38 – 1.35 (m, 2H)
^{13}C-NMR (101 MHz, DMSO-d6), δ [ppm]: 197.8, 181.1, 169.0, 139.0, 137.3, 129.1, 124.0, 123.4, 118.8, 62.7, 54.4, 26.8, 25.4, 23.7,
IR (ATR, 4000 – 400 cm-1), ν [cm-1]: 3356, 3305, 3253, 3171, 3039, 2999, 2951, 2933, 2874, 2851, 2808, 2752, 1681, 1665, 1644, 1618, 1602, 1588, 1517, 1469, 1436, 1423, 1406, 1384, 1357, 1332, 1315, 1300, 1271, 1208, 1179, 1163, 1152, 1124, 1113, 1087, 1075, 1054

MS (ESI) m/z: 334.20 $[M+H]^+$.

N-{3-[1-[(carbamothioylamino)imino]ethyl]phenyl}-2-(morpholine-4-yl)acetamide (23-8-1). Yield 62%, mp=241.2 – 241.8 °C.
^{1}H-NMR (400 MHz, DMSO-d6), δ [ppm]: 9.90 (s, 1H), 8.63 (s, 1H), 8.21 (d J = 4 Hz ,1H), 7.87 (t J = 2 Hz, 1H), 7.64 (t J = 4 Hz, 1H), 7.44 (t J = 4 Hz, 1H), 4.61 (s, 2H), 3.62 – 3.60 (m, 4H), 3.09 (s, 2H), 2.58 – 2.55 (m, 4H), 2.31 (s, 3H)

^{13}C-NMR (101 MHz, DMSO-d6), δ [ppm]: 196.9, 181.1, 167.8, 146.8, 139.6, 137.8, 128.2, 123.6, 118.9, 68.1, 65.5, 53.1, 25.3
IR (ATR, 4000 – 400 cm-1), ν [cm-1]: 3356, 3269, 3170, 2957, 2935, 2873, 2821, 1677, 1667, 1645, 1603, 1519, 1495, 1473, 1406, 1360, 1340, 1316, 1274, 1209, 1181, 1157, 1119, 1108, 1072

MS (ESI) m/z: 336.19 $[M+H]^+$.

N-{3-[1-[(carbamothioylamino)imino]ethyl]phenyl}-2-(morpholine-4-yl)acetamide (23-11-1). Yield 69%, mp=232.5 – 233.1 °C.
^{1}H-NMR (400 MHz, DMSO-d6), δ [ppm]: 9.64 (s, 1H), 8.28 (s, 1H), 7.47 (d J = 4 Hz ,1H), 7.75 (t J = 2 Hz, 1H), 7.61 (t J = 4 Hz, 1H), 7.30 (t J = 4 Hz, 1H), 3.10 (s, 2H), 2.62 – 2.48 (m, 8H), 2.37 (s, 2H), 2.27 (s, 3H), 2.16 (s, 3H)
^{13}C-NMR (101 MHz, DMSO-d6), δ [ppm]: 179.0, 168.4, 147.5, 138.6, 138.2, 128.6, 121.7, 120.2, 117.5, 61.9, 54.4, 52.7, 45.7, 14.0
IR (ATR, 4000 – 400 cm-1), ν [cm-1]: 3358, 3275, 3170, 2950, 2937, 2875, 2826, 1659, 1669, 1643, 1605, 1518, 1497, 1471, 1409,1360, 1341, 1313, 1275, 1210, 1180, 1160, 1120, 1114, 1073

MS (ESI) m/z: 349.22 $[M+H]^+$.

N-{4-[1-[(carbamothioylamino)imino]ethyl]phenyl}-2-(dipropylamino)acetamide (24-3-1). Yield 61%, mp=223.1 – 223.8 °C.
^{1}H-NMR (400 MHz, DMSO-d6), δ [ppm]: 9.80 (s, 1H), 8.54 (s, 1H), 7.83 (d J = 8 Hz, 2H), 7.66 (d J = 8 Hz, 2H), 4.40 (s, 2H), 3.12 (s, 2H), 2.43 – 2.38(m, 7H), 1.40 – 1.31 (m, 4H), 0.76 (t J = 8 Hz, 6H)
^{13}C-NMR (101 MHz, DMSO-d6), δ [ppm]: 196.5, 181.2, 170.5, 142.7, 131.9, 129.5, 118.4, 58.4, 56.4, 26.5, 19.9, 11.8
IR (ATR, 4000 – 400 cm-1), ν [cm-1]: 3357, 3272, 3169, 2955, 2934, 2871, 2823, 1679, 1667, 1644, 1602, 1518, 1494, 1470, 1405, 1359, 1337, 1315, 1272, 1211, 1179, 1159, 1120, 110, 1075

MS (ESI) m/z: 350.22 $[M+H]^+$.

N-{4-[1-[(carbamothioylamino)imino]ethyl]phenyl}-2-(dibutylamino)acetamide (24-4-1). Yield 53%, mp=228.7 – 229.3 °C;
^{1}H-NMR (400 MHz, DMSO-d6), δ [ppm]: 9.90 (s, 1H), 8.64 (s, 1H), 7.93 (d J = 8 Hz, 2H), 7.76 (d J = 8 Hz, 2H), 4.49 (s, 2H), 3.21 (s, 2H), 2.58 – 2.54 (m, 4H), 2.52 (s, 3H), 1.46 – 1.42 (m, 4H), 1.30 – 1.24 (m, 4H), 0.86 (t J =6 Hz,6H)
^{13}C-NMR (101 MHz, DMSO-d6), δ [ppm]: 196.5, 181.5, 170.4, 142.7, 131.9, 129.5, 118.4, 58.4, 54.1, 28.7, 26.4, 20.0, 13.9
IR (ATR, 4000 – 400 cm-1), ν [cm-1]: 3358, 3261, 3171, 2957, 2930, 2872, 2830, 1679, 1668, 1643, 1618, 1601, 1517, 1493, 1405, 1360, 1336, 1314, 1270, 1179, 1152, 1083

MS (ESI) m/z: 378.25 $[M+H]^+$.

N-{4-[1-[(carbamothioylamino)imino]ethyl]phenyl}-2-(pyrrolidine-1-yl)acetamide (24-5-1). Yield 53%, mp=234.7 – 234.9 °C;

^{1}H-NMR (400 MHz, DMSO-d6), δ [ppm]: 11.02 (s, 1H), 10.18 (s, 1H), 8.26 (s, 1H), 7.92 (d J= 8 Hz, 2H), 7.65 (d J = 8 Hz, 2H), 4.27 (s, 3H), 3.42 – 3.25 (m, 4H), 2.27 (s, 3H), 1.96 – 1.91 (m, 4H)
^{13}C-NMR (101 MHz, DMSO-d6), δ [ppm]: 178.8, 163.7, 147.3, 139.0, 133.1, 127.4, 118.8, 55.9, 54.1, 22.8, 13.7
IR (ATR, 4000 – 400 cm-1), ν [cm-1]: 3417, 3223, 3145, 3053, 2939, 2851, 2821, 1690, 1580, 1537, 1515, 1453, 1408, 1359, 1335, 1304, 1261, 1188, 1113, 1068, 1033, 1008, 985

MS (ESI) m/z: 319.93 $[M+H]^+$.

N-{4-[1-[(carbamothioylamino)imino]ethyl]phenyl}-2-(piperidine-1-yl)acetamide (24-6-1). Yield 60%, mp=238.7 – 239.2 °C;
^{1}H-NMR (400 MHz, DMSO-d6), δ [ppm]: 10.00 (s, 1H), 8.64 (s, 1H), 7.92 (d J = 8 Hz, 2H), 7.78 (d J = 8 Hz, 2H), 4.49 (s, 2H), 3.11 (s, 2H), 2.52 (s, 3H), 2.49 – 2.46 (m, 4H), 1.58 – 1.54 (m, 4H), 1.42 – 1.39 (m, 2H)
^{13}C-NMR (101 MHz, DMSO-d6), δ [ppm]: 196.5, 181.2, 169.2, 142.9, 131.8, 129.4, 118.6, 62.6, 54.0, 26.5, 25.4, 23.5
IR (ATR, 4000 – 400 cm-1), ν [cm-1]: 3254, 3163, 2933, 2852, 2784, 1685, 1667, 1606, 1583, 1521, 1442, 1406, 1349, 1314, 1272, 1188, 1157, 1116, 1075

MS (ESI) m/z: 334.27 $[M+H]^+$.

2-(azepane-1-yl)-N-{4-[1-[(carbamothioylamino)imino]ethyl]phenyl}acetamide (24-7-1). Yield 49%, mp=245.1 – 245.7 °C;
^{1}H-NMR (400 MHz, DMSO-d6), δ [ppm]: 9.99 (s, 1H), 8.64 (s, 1H), 7.92 (d J = 8 Hz, 2H), 7.78 (d J = 8 Hz, 2H), 4.49 (s, 2H), 3.32 (d J = 12 Hz, 2H), 2.74 – 2.71 (m, 4H), 2.52 (s, 3H), 1.62 – 1.56 (m, 8H)
^{13}C-NMR (101 MHz, DMSO-d6), δ [ppm]: 196.5, 181.2, 170.0, 142.9, 131.8, 129.5, 118.5, 61.6, 55.1, 27.7, 26.6, 26.5
IR (ATR, 4000 – 400 cm-1), ν [cm-1]: 3363, 3263, 3171, 2933, 2914, 2856, 1679, 1643, 1618, 1602, 1518, 1493, 1455, 1404, 1359, 1316, 1272, 1178, 1154, 1124, 1091, 1086

MS (ESI) m/z: 348.14 $[M+H]^+$.

N-{4-[1-[(carbamothioylamino)imino]ethyl]phenyl}-2-(morpholine-4-yl)acetamide (24-8-1). Yield 65%, mp=248.6 – 249.2 °C;
^{1}H-NMR (400 MHz, DMSO-d6), δ [ppm]: 10.16 (s, 1H), 9.86 (s, 1H), 8.25 (s, 1H), 7.89 (d J=8,8 Hz, 2H), 7.64 (d J = 9,2 Hz, 2H), 3.62 (t J = 4,6 Hz, 4H), 3.34 (s, 1H), 3.13 (s, 2H), 2.49 (s, 4H), 2.25 (s, 3H)
^{13}C-NMR (101 MHz, DMSO-d6), δ [ppm]: 196.9, 181.1, 167.8, 146.8, 139.6, 127.2, 118.9, 68.1, 65.5, 53.1, 26.5
IR (ATR, 4000 – 400 cm-1), ν [cm-1]: 3367, 3259, 3169, 3001, 2974, 2913, 1680, 1665, 1642, 1618, 1602, 1618, 1582, 1485, 1455, 1404, 1352, 1317, 1286, 1162, 1119, 1043, 995, 954, 861, 801

MS (ESI) m/z: 336.12 $[M+H]^+$.

N-{4-[1-[(carbamothioylamino)imino]ethyl]phenyl}-2-(thiomorpholine-4-yl)acetamide (24-9-1). Yield 62,6%, mp=254.4 – 254.9 °C;

^{1}H-NMR (400 MHz, DMSO-d6), δ [ppm]: 10.01 (s, 1H), 7.92 (d J = 8 Hz, 2H), 7.79 (d J = 8 Hz, 2H), 4.50 (s, 3H), 3.19 (s, 2H), 2.77 – 2.75 (m, 4H), 2.68 – 2.64 (m, 4H), 2.52 (s, 3H)
^{13}C-NMR (101 MHz, DMSO-d6), δ [ppm]: 196.2, 181.2, 170.3, 142.9, 131.9, 129.5, 118.8, 62.4, 54.5, 27.0, 26.5
IR (ATR, 4000 – 400 cm-1), ν [cm-1]: 3273, 3213, 3183, 3002, 2958, 2827, 1681, 1667, 1644, 1606, 1583, 1506, 1458, 1406, 1390, 1348, 1313, 1272, 1251, 1190, 1131, 1075, 1010, 975, 945, 895, 879, 819, 787

MS (ESI) m/z: 352.18 $[M+H]^{+}$.

22-2-2 to 24-14-2

1-[({4-[1-[(carbamothioylamino)imino]ethyl]phenyl}carbamoyl)methyl]diethylazanium chloride (24-2-2). Yield 59 %, mp=228.9 – 230.3 °C.
^{1}H-NMR (400 MHz, DMSO-d6), δ [ppm]: 11.29 (s, 1H), 10.19 (s, 1H), 9.78 (s, 1H), 8.27 (s, 1H), 7.93 (d J = 8,8 Hz, 2H), 7.65 (d J = 8,8 Hz, 2H), 4.20 (s, 2H), 3.62 (s, 1H), 3.14 (d J = 10 Hz, 4H), 2.26 (s, 3H), 1.26 (t J = 7.6 Hz, 6H)
^{13}C-NMR (101 MHz, DMSO-d6), δ [ppm]: 178.7, 163.5, 147.2, 139.0, 133.2, 127.3, 118.8, 58.1, 53.9, 16.9, 10.8
IR (ATR, 4000 – 400 cm-1), ν [cm-1]: 3274, 3212, 3183, 3000, 2958, 2828, 1682, 1667, 1645, 1606, 1584, 1507, 1459, 1409, 1392, 1350, 1316, 1274, 1250, 1190, 1133, 1074, 1012, 976, 947, 896, 875, 817, 789

MS (ESI) m/z: 322.08 $[M+H]^{+}$.

1-[({4-[-1-[(carbamothioylamino)imino]ethyl]phenyl}carbamoyl)methyl]-4-methylpiperazine-1,4-diium dichloride (24-11-2). Yield 71%, mp=204.3 – 205.7 °C.
^{1}H-NMR (400 MHz, DMSO-d6), δ [ppm]: 11.76 (s, 1H), 10.94 (s, 1H), 10.18 (s, 1H), 8.27 (s, 1H), 7.95 (d J = 8,4 Hz, 2H), 7.65 (d J = 8,8 Hz, 2H), 4.13 (s, 2H), 3.63 – 3,39 (m, 8H), 2.81 (s, 3H), 2.53 (s, 2H), 2.26 (s, 3H)
^{13}C-NMR (101 MHz, DMSO-d6), δ [ppm]: 178.7, 163.5, 147.2, 139.0, 133.2, 127.4, 118.8, 55.4, 53.9, 16.9, 13.8, 10.8
IR (ATR, 4000 – 400 cm-1), ν [cm-1]: 3270, 3216, 3182, 3004, 2960, 2826, 1681, 1667, 1645, 1607, 1584, 1507, 1459, 1407, 1392, 1348, 1315, 1273, 1250, 1188, 1130, 1074, 1012, 974, 946, 896, 878, 817, 788

MS (ESI) m/z: 349.24 $[M+H]^{+}$.

22-2-3 to 24-14-3

1-[({3-[1-[(carbamothioylamino)imino]ethyl]phenyl}carbamoyl)azepan-1-ium perchlorate (23-7-3). Yield 59%, mp=208.9 – 210.0 °C.
^{1}H-NMR (400 MHz, DMSO-d6), δ [ppm]: 10.68 (s, 1H), 10.18 (s, 1H), 9.45 (s, 1H), 8.28 (s, 1H), 8.19 (d J = 4 Hz ,1H), 7.87 (t J = 2 Hz, 1H), 7.55 (t J = 4 Hz, 1H), 7.19 (t J = 4 Hz, 1H), 4.40 (s, 3H), 3.08 (s, 2H), 2.53 (s, 3H), 2.43 – 2.39 (m, 4H), 1.56 – 1.52 (m, 4H), 1.36 – 1.33 (m, 2H)
^{13}C-NMR (101 MHz, DMSO-d6), δ [ppm]: 178.8, 163.4, 148.0, 138.0, 134.3, 127.1, 123.9, 123.1, 118.8, 62.7, 54.9, 26.3, 25.1, 21.6,

IR (ATR, 4000 – 400 cm-1), ν [cm-1]: 3440, 3320, 3150, 3004, 2960, 2826, 1681, 1667, 1640, 1607, 1584, 1507, 1459, 1407, 1380, 1348, 1315, 1275, 1250, 1188, 1120, 1074, 1012, 974, 946, 896, 878
MS (ESI) m/z: 348.05 $[M+H]^+$.

1-[({4-[1-[(carbamothioylamino)imino]ethyl]phenyl}carbamoyl)methyl]diethylazanium perchlorate (24-2-3). Yield 68%, mp=231.3 – 232.7 °C.
^{1}H-NMR (400 MHz, DMSO-d6), δ [ppm]: 10.68 (s, 1H), 10.20 (s, 1H), 9.45 (s, 1H), 8.28 (s, 1H), 7.95 (d J = 8,4 Hz, 2H), 7.59 (d J = 8,8 Hz, 2H), 4.10 (s, 2H), 3.23 – 3,20 (m, 4H), 2.50 - 2.49 (m, 1H), 2.27 (s, 3H), 1.22 (t J = 7,4 Hz, 6H)
^{13}C-NMR (101 MHz, DMSO-d6), δ [ppm]: 178.8, 163.5, 147.0, 138.7, 133.4, 127.5, 118.7, 53.5, 48.6, 13.7, 8.8
IR (ATR, 4000 – 400 cm-1), ν [cm-1]: 3441, 3321, 3149, 3005, 2965, 2827, 1680, 1667, 1640, 1610, 1585, 1506, 1461, 1407, 1381, 1349, 1317, 1276, 1248, 1190, 1121, 1075, 1013, 973, 945, 897, 880
MS (ESI) m/z: 322.28 $[M+H]^+$.

1-[({4-[1-[(carbamothioylamino)imino]ethyl]phenyl}carbamoyl)methyl]pyrrolidin-1-ium perchlorate (24-5-3). Yield 71%, mp=236.8 – 237.7 °C.
^{1}H-NMR (400 MHz, DMSO-d6), δ [ppm]: 10.61 (s, 1H), 10.20 (s, 1H), 9.99 (s, 1H), 8.27 (s, 1H), 7.94 (d J = 8,4 Hz, 2H), 7.59 (d J = 8,4 Hz, 2H), 4.23 (s, 3H), 3.38 – 3.29 (m, 4H), 2.26 (s, 3H), 1.96 – 1.91 (m, 4H)
^{13}C-NMR (101 MHz, DMSO-d6), δ [ppm]: 178.8, 163.8, 147.2, 138.8, 133.2, 127.5, 118.8, 56.1, 54.3, 22.7, 13.8
IR (ATR, 4000 – 400 cm-1), ν [cm-1]: 3439, 3322, 3151, 3006, 2962, 2830, 1682, 1666, 1639, 1610, 1586, 1510, 1460, 1410, 1381, 1350, 1317, 1277, 1252, 1189, 1125, 1075, 1011, 972, 947, 895, 875
MS (ESI) m/z: 320.18 $[M+H]^+$.

1-[({4-[1-[(carbamothioylamino)imino]ethyl]phenyl}carbamoyl)methyl]piperidin-1-ium perchlorate (24-6-3). Yield 74%, mp=254.3 – 255.1 °C.
^{1}H-NMR (400 MHz, DMSO-d6), δ [ppm]: 11.28 (s, 1H), 10.19 (s, 1H), 10.06 (s, 1H), 8.27 (s, 1H), 7.93 (d J = 8,0 Hz, 2H), 7.67 (d J = 8,4 Hz, 2H), 4.17 (s, 2H), 3.48 – 3.36 (m, 4H), 3.15 – 3.08 (m, 2H), 2.26 (s, 3H), 1.78 – 1.66 (m, 5H)
^{13}C-NMR (101 MHz, DMSO-d6), δ [ppm]: 178.8, 163.1, 147.2, 138.9, 133.3, 127.4, 118.8, 57.1, 53.0, 22.2, 17.9, 13.7
IR (ATR, 4000 – 400 cm-1), ν [cm-1]: 3442, 3319, 3153, 3004, 2962, 2826, 1682, 1667, 1639, 1607, 1585, 1509, 1458, 1406, 1381, 1348, 1313, 1275, 1255, 1185, 1119, 1075, 1015, 972, 943, 898, 876
MS (ESI) m/z: 334.1434 $[M+H]^+$.

4-benzyl-1-[({4-1-[(carbamothioylamino)imino]ethyl]phenyl}carbamoyl)methyl]piperazine-1,4-diium diperchlorate (24-10-3). Yield 58%, mp=261.1 – 260.4 °C.

^{1}H-NMR (400 MHz, DMSO-d6), δ [ppm]: 10.46 (s, 1H), 10.20 (s, 1H), 8.28 (s, 1H), 7.92 (d J= 8,8 Hz, 2H), 7.59 (d J = 8,2 Hz, 2H), 7.52 – 7.48 (m, 5H), 4.36 (s, 2H), 3.99 – 3.24 (m, 14H), 2.26 (s, 3H)
^{13}C-NMR (101 MHz, DMSO-d6), δ [ppm]: 178.8, 147.2, 138.8, 133.2, 131.4, 129.8, 129.0, 127.4, 118.9, 13.8
IR (ATR, 4000 – 400 cm-1), ν [cm-1]: 3443, 3320, 3148, 3000, 2958, 2827, 1680, 1668, 1640, 1609, 1585, 1510, 1461, 1407, 1382, 1348, 1316, 1276, 1251, 1190, 1123, 1075, 1013, 976, 942, 899, 881
MS (ESI) m/z: 425.09 $[M+H]^+$.

1-[({4-[1-[(carbamothioylamino)imino]ethyl]phenyl}carbamoyl)methyl]-4-methylpiperazine-1,4-diium diperchlorate (24-11-3). Yield 63%, mp=262.3 – 262.9 °C.
^{1}H-NMR (400 MHz, DMSO-d6), δ [ppm]: 10.76 (s, 1H), 10.20 (s, 1H), 9.84 (s, 1H), 8.28 (s, 1H), 7.95 (d J = 8,4 Hz, 2H), 7.65 (d J = 8,8 Hz, 2H), 4.13 (s, 2H), 3.63 – 3.39 (m, 8H), 2.81 (s, 3H), 2.53 (s, 2H), 2.26 (s, 3H)
^{13}C-NMR (101 MHz, DMSO-d6), δ [ppm]: 178.7, 163.5, 147.2, 138.9, 133.2, 127.3, 118.8, 55.4, 53.9, 16.8, 13.8, 10.8
IR (ATR, 4000 – 400 cm-1), ν [cm-1]: 3441, 3321, 3148, 3002, 2964, 2827, 1680, 1665, 1641, 1608, 1585, 1509, 1458, 1405, 1383, 1349, 1315, 1271, 1249, 1190, 1119, 1075, 1012, 975, 944, 894, 882
MS (ESI) m/z: 349.24 $[M+H]^+$.

32-2-1 to 34-14-1

N-{4-[1-[(carbamoylamino)imino]ethyl]phenyl}-2-(diethylamino)acetamide (34-2-1). Yield 45%, mp=175.2 – 176.1 °C.
^{1}H-NMR (400 MHz, DMSO-d6), δ [ppm]: 9.96 (s, 1H), 8.62 (s, 1H), 7.91 (d, J = 8.5 Hz, 2H), 7.78 (d J= 8.5 Hz, 2H), 4.48 (s, 2H), 3.20 (s, 2H), 2.61 (q J=14.4 Hz, 4H), 2.57 (s, 3H), 0.99 (t, J=7.2 Hz, 6H)
^{13}C-NMR (101 MHz, DMSO-d6), δ [ppm]: 181.18, 171.525, 142.76, 131.90, 129.46, 118.63, 57.33, 47.85, 24.50, 11.88
IR (ATR, 4000 – 400 cm-1), ν [cm-1]: 3458, 3310, 3144, 2908, 1690, 1627, 1582, 1516, 1457, 1405, 1335, 1310, 1294, 1258, 1181, 1129, 1100, 1001, 845, 822, 765, 744, 691, 624, 611, 549

MS (ESI) m/z: 306.23 $[M+H]^+$.

N-{4-[1-[(carbamoylamino)imino]ethyl]phenyl}-2-(piperidine-1-yl)acetamide (34-6-1). Yield 36%, mp=189.1 – 190.2 °C.
^{1}H-NMR (400 MHz, DMSO-d6), δ [ppm]: 9.82 (s, 1H), 9.28 (s, 1H), 7.77 (d J = 8,4 Hz, 2H), 7.64 (d J = 8,6 Hz, 2H), 6.47 (s, 2H), 3.26 (s, 2H), 2.64 (s, 3H), 2.44 – 2.41 (m, 4H), 1.92 – 1.82 (m, 4H), 1.48 – 1.43 (m, 2H)
^{13}C-NMR (101 MHz, DMSO-d6), δ [ppm]: 169.5, 157.3, 143.7, 138.9, 133.1, 126.4, 118.6, 61.7, 55.2, 27.8, 26.5, 13.2
IR (ATR, 4000 – 400 cm-1), ν [cm-1]: 3462, 3307, 3144, 2906, 1688, 1623, 1578, 1514, 1455, 1405, 1335, 1305, 1296, 1254, 1181, 1126, 1103, 996, 842, 826, 763, 744, 687, 628, 609, 556

MS (ESI) m/z: 317.95 $[M+H]^+$.

N-{4-[1-[(carbamoylamino)imino]ethyl]phenyl}-2-(azepane-1-yl)acetamide (34-7-1). Yield 21%, mp=212.4 – 214.1 °C.
^{1}H-NMR (400 MHz, DMSO-d6), δ [ppm]: 9.82 (s, 1H), 9.28 (s, 1H), 7.77 (d J = 8,4 Hz, 2H), 7.64 (d J = 8,6 Hz, 2H), 6.47 (s, 2H), 3.26 (s, 2H), 2.71 – 2.68 (m, 4H), 2.14 (s, 3H), 1.60 - 1.57 (m, 8H)
^{13}C-NMR (400 MHz, DMSO-d6), δ [ppm]: 169.5, 157.3, 143.7, 138.9, 133.1, 126.4, 118.6, 61.7, 55.2, 27.8, 26.5, 13.2
IR (ATR, 4000 – 400 cm-1), ν [cm-1]: 3462, 3307, 3144, 2906, 1688, 1623, 1578, 1514, 1455, 1405, 1335, 1305, 1296, 1254, 1181, 1126, 1103, 996, 964, 842, 826, 763, 744, 687, 628, 609, 556

MS (ESI) m/z: 332.27 $[M+H]^{+}$.

N-{4-[1-[(carbamoylamino)imino]ethyl]phenyl}-2-(morpholine-4-yl)acetamide (34-8-1). Yield 19%, mp=231.1 – 231.8 °C.
^{1}H-NMR (400 MHz, DMSO-d6), δ [ppm]: 9.92 (s, 1H), 9.26 (s, 1H), 7.78 (d J = 8 Hz, 2H), 7.65 (d J = 8 Hz, 2H), 6.46 (s, 2H), 3.42 – 3.27 (m, 8H), 3.14 (s, 2H), 2.15 (s, 3H)
^{13}C-NMR (101 MHz, DMSO-d6), δ [ppm]: 168.2, 157.3, 143.7, 138.9, 133.2, 126.4, 118.8, 66.1, 62.0, 53.2, 13.1
IR (ATR, 4000 – 400 cm-1), ν [cm-1]: 3463, 3281, 3132, 2958, 2938, 2852, 2823, 1688, 1625, 1580, 1517, 1455, 1407, 1374, 1336, 1322, 1307, 1268, 1256, 1230, 1207, 1188, 1140, 1112, 1068, 1032, 1009, 988

MS (ESI) m/z: 320.21 $[M+H]^{+}$.

Examples of 1D and 2D NMR spectra of selected compounds

12-1

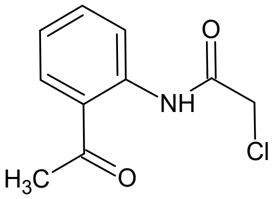

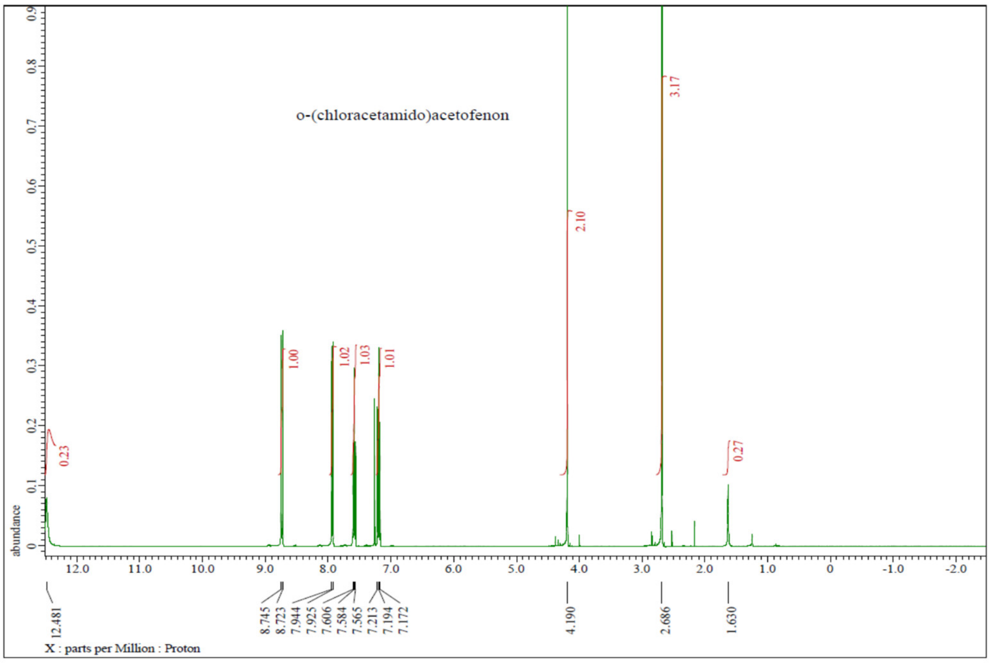

1H- NMR at 400 MHz

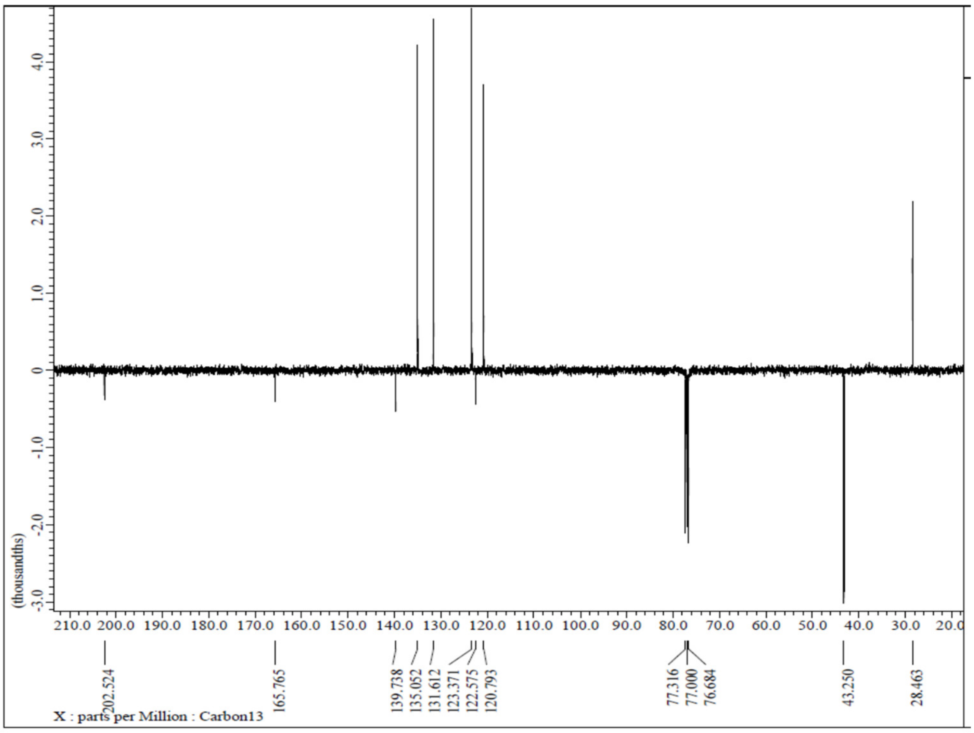

13C-apt at 100 MHz

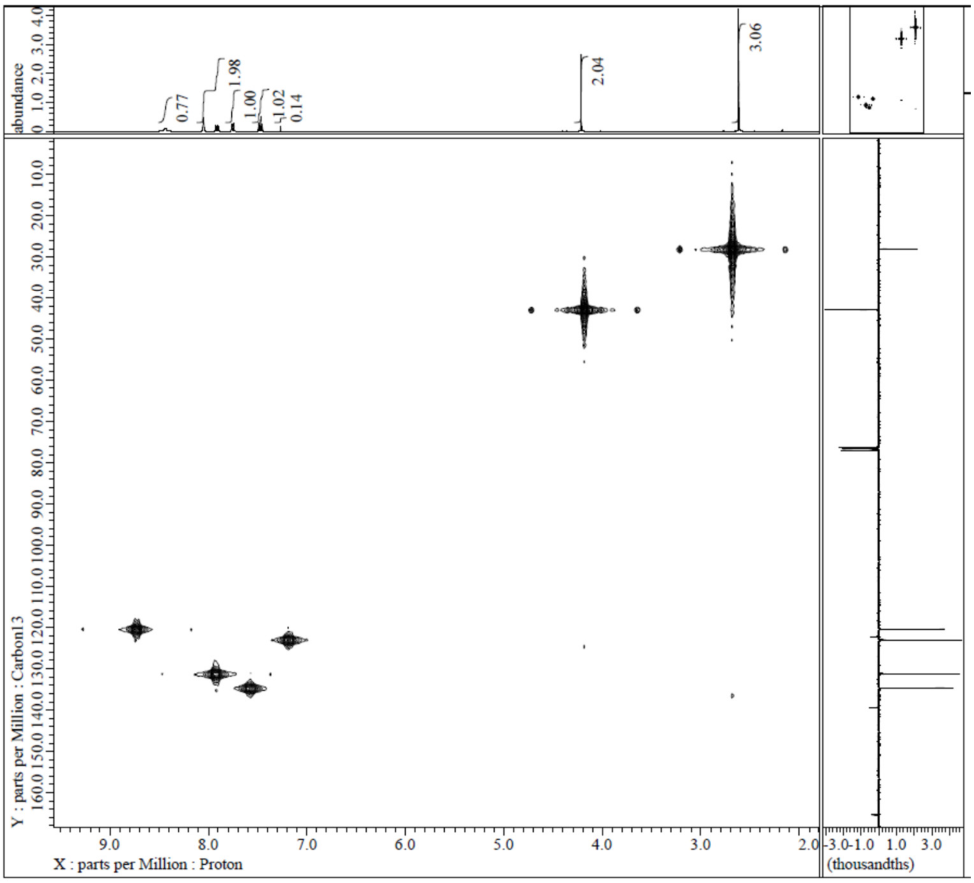

HMQC (2D; C-H correlation)

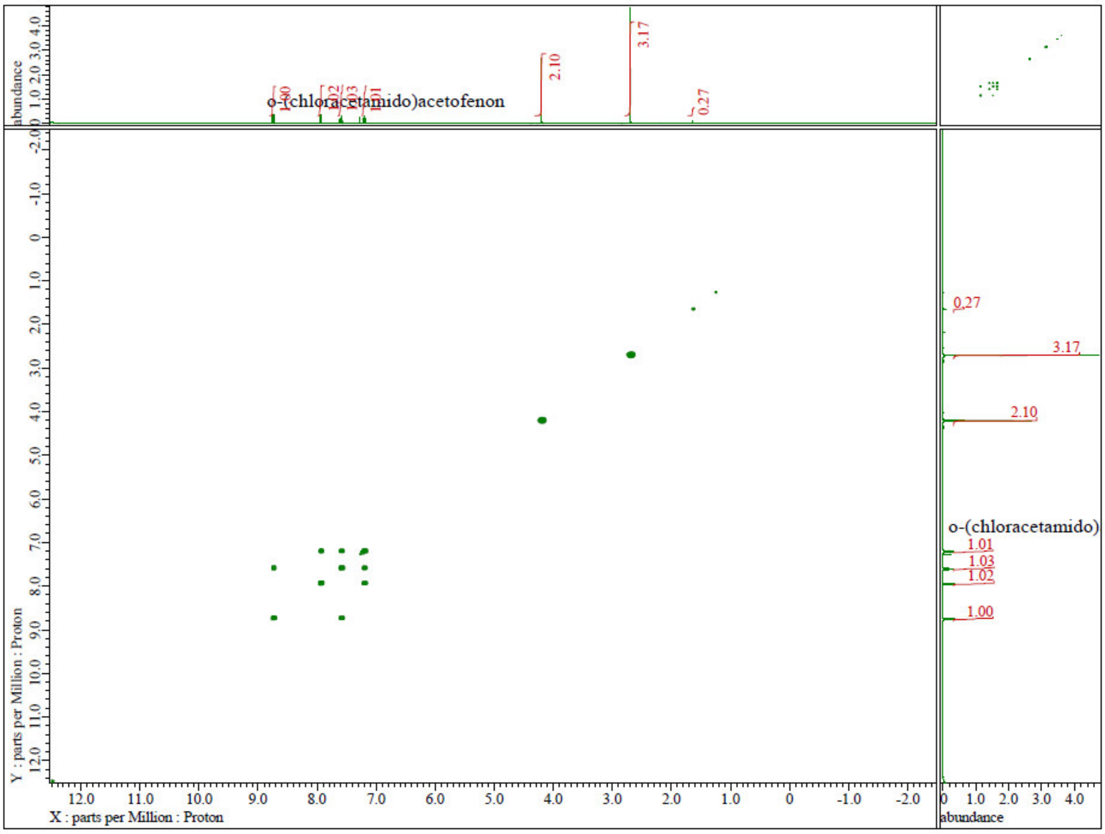

H-H cosy (2D)

13-1

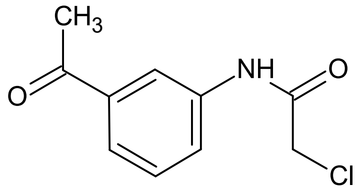

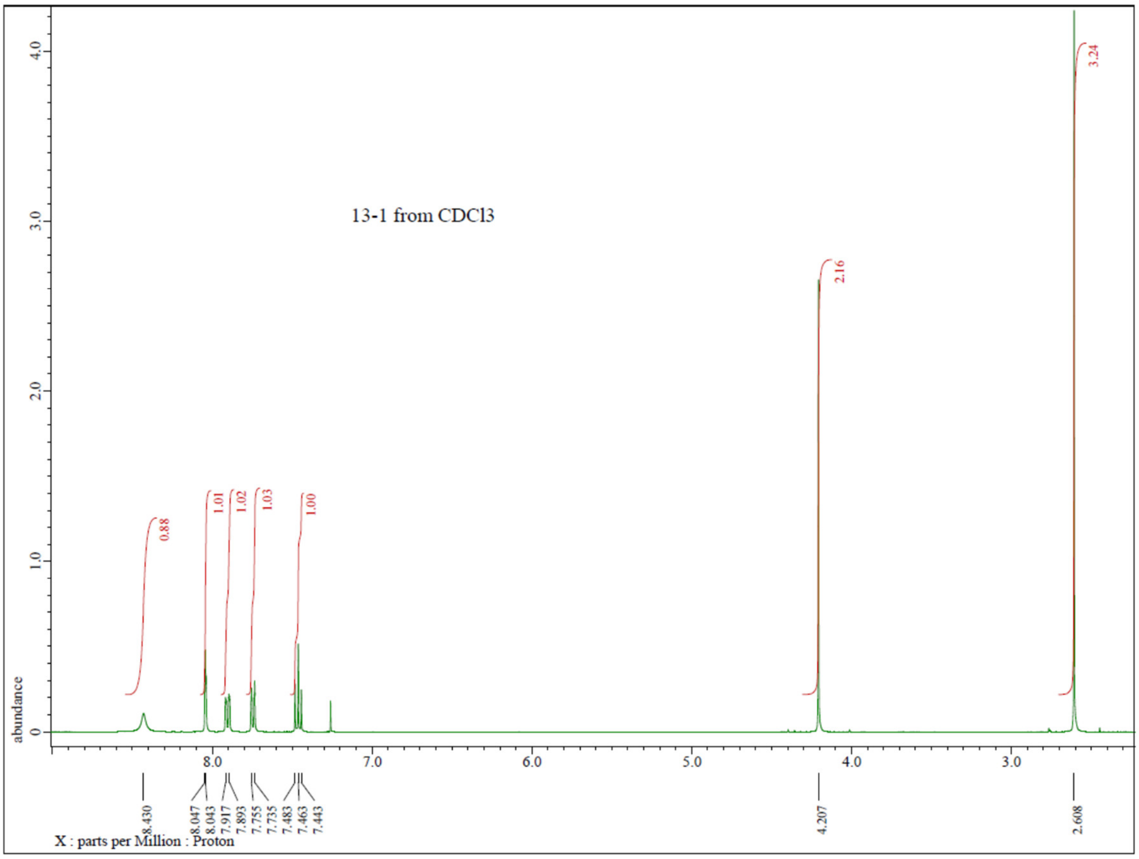

1H-NMR at 400 MHz

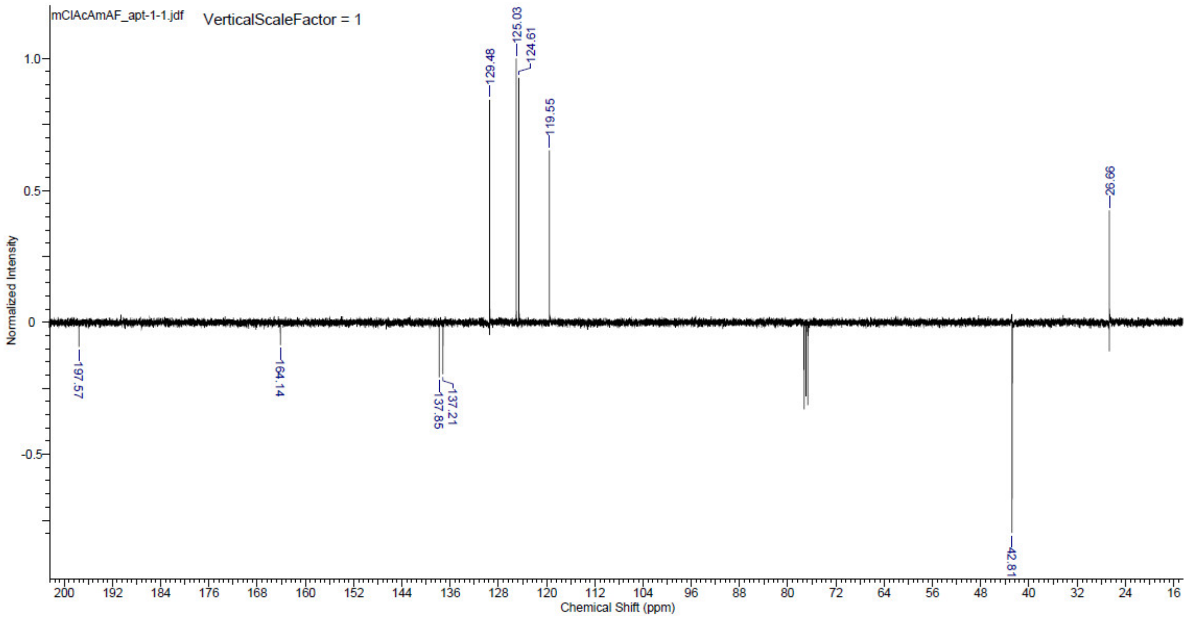

13C-apt at 100 MHz

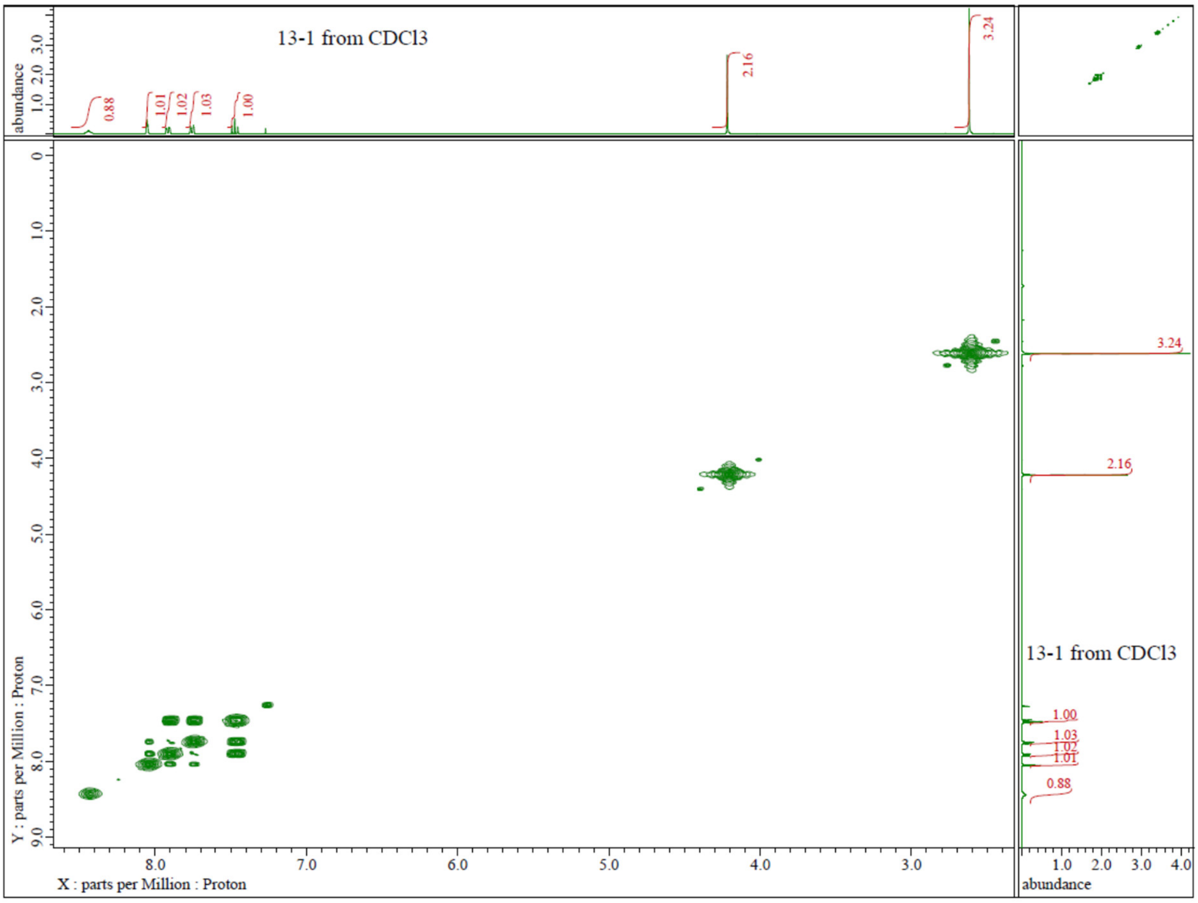
13-1 from CDCl3
abundance
0.88
1.01
1.02
1.03
1.00
2.16
3.24
Y : parts per Million : Proton
X : parts per Million : Proton
13-1 from CDCl3
abundance

H-H cosy (2D)

14-1

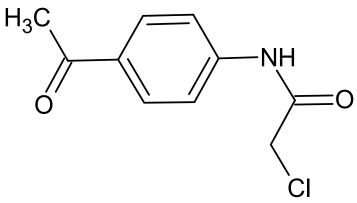

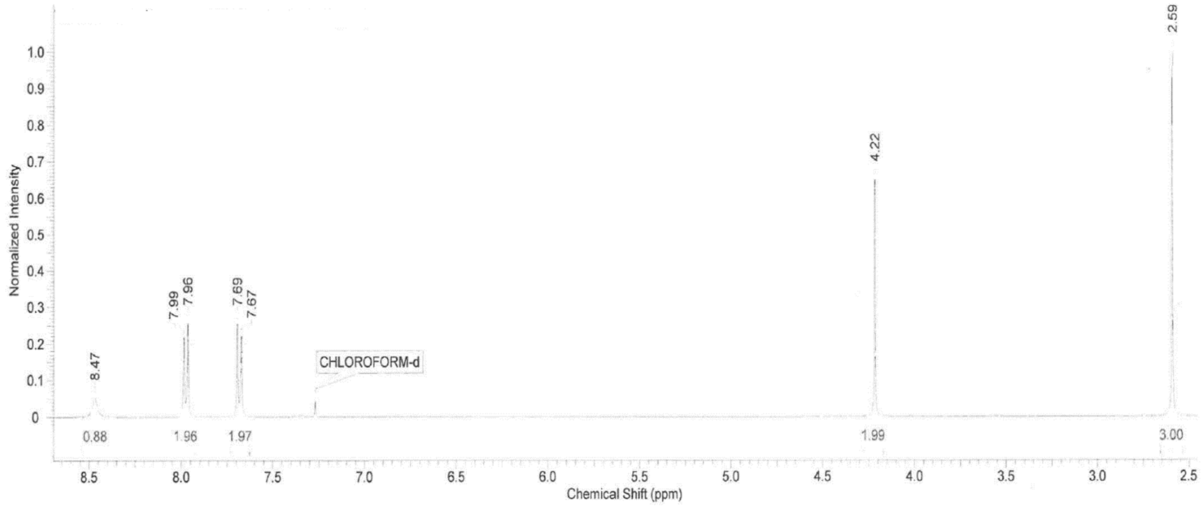

1H-NMR at 400 MHz

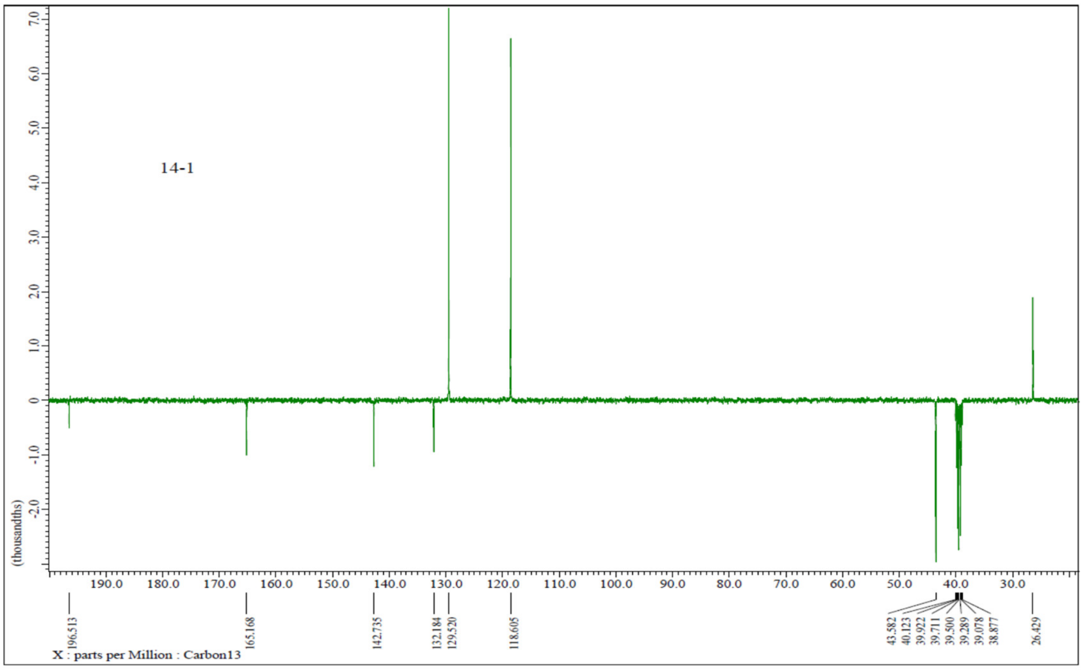

13C-apt at 100 MHz

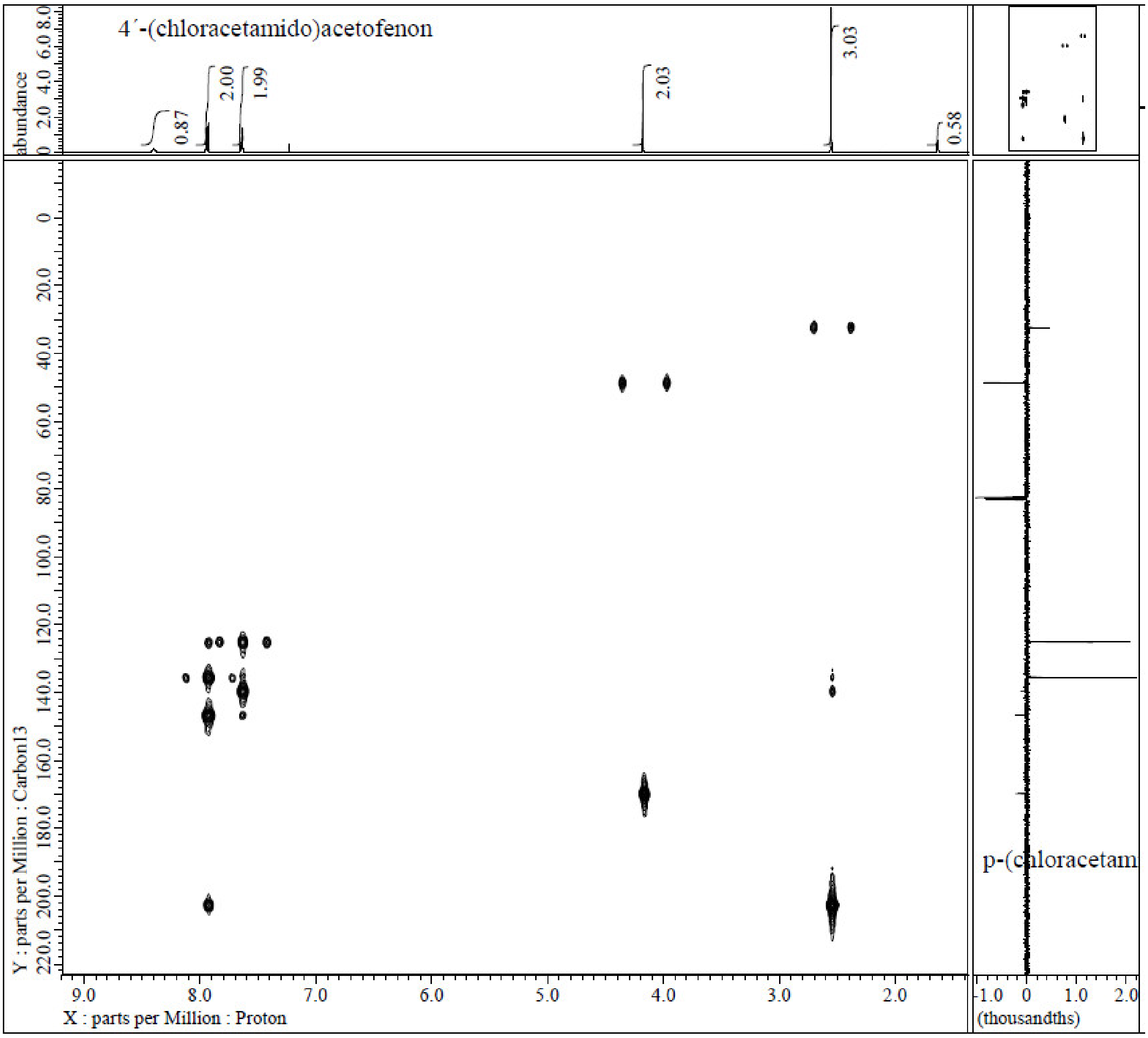

4´-(chloracetamido)acetofenon
abundance
0.87
2.00
1.99
2.03
3.03
0.58
Y : parts per Million : Carbon13
X : parts per Million : Proton
(thousandths)
p-(chloracetam

HMBC

14-2-1

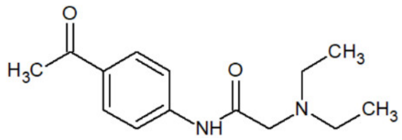

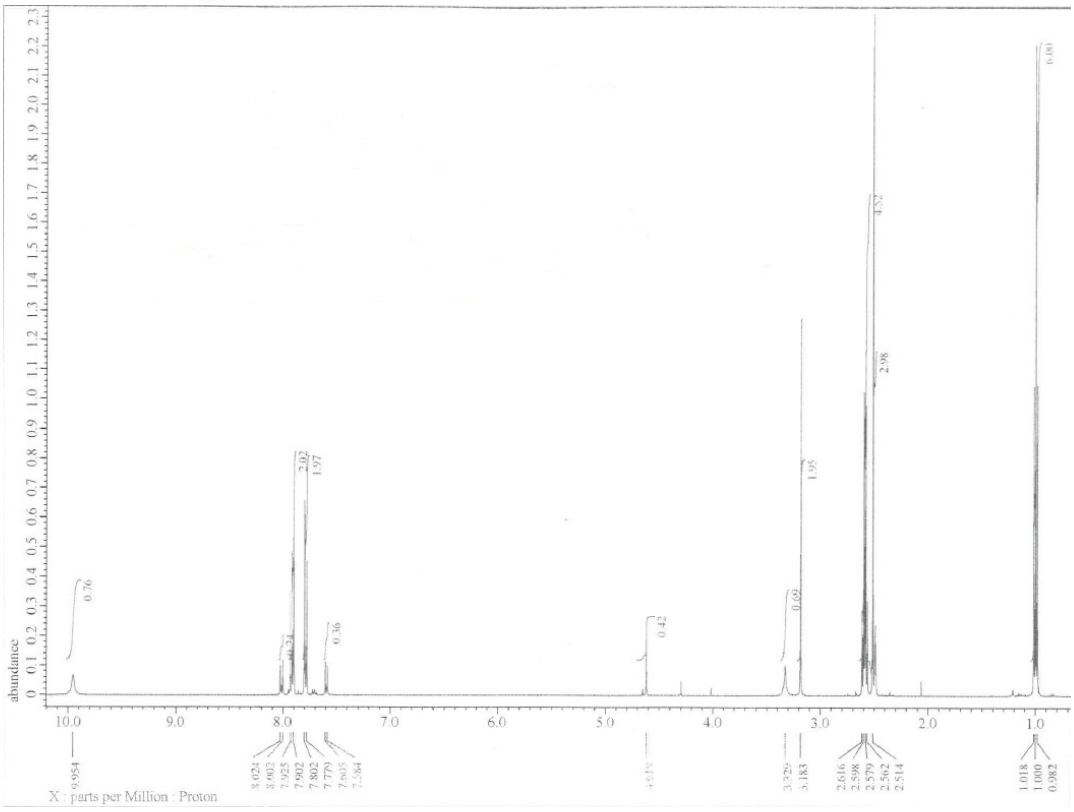

1H-NMR at 400 MHz

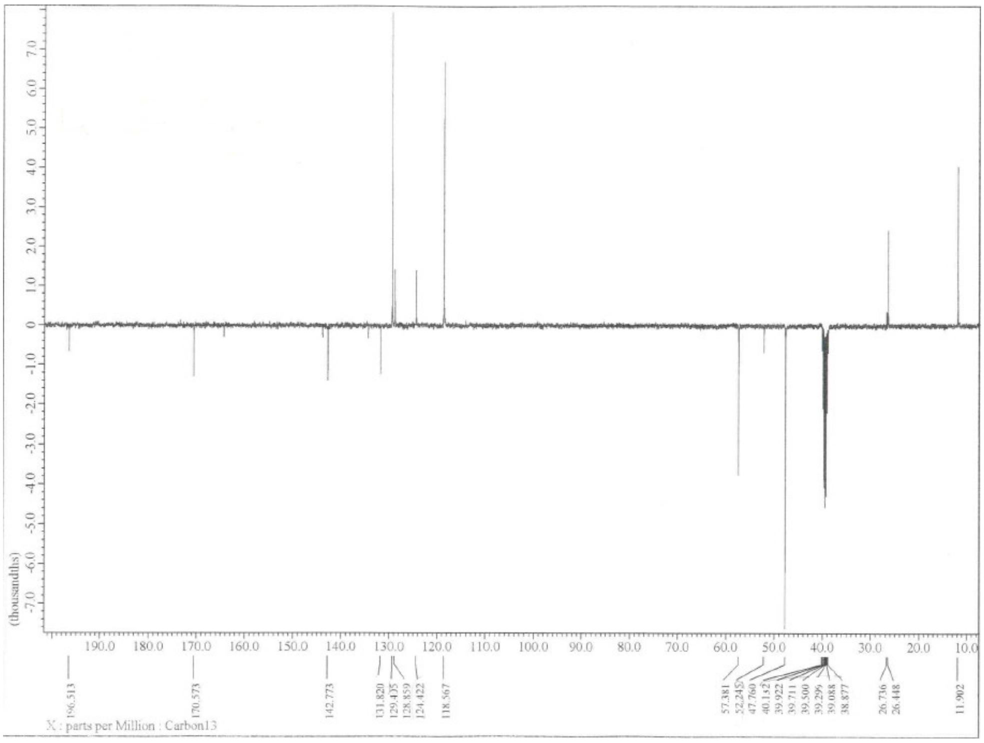

13C-apt at 100 MHz

14-6-1

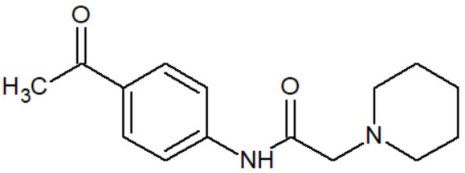

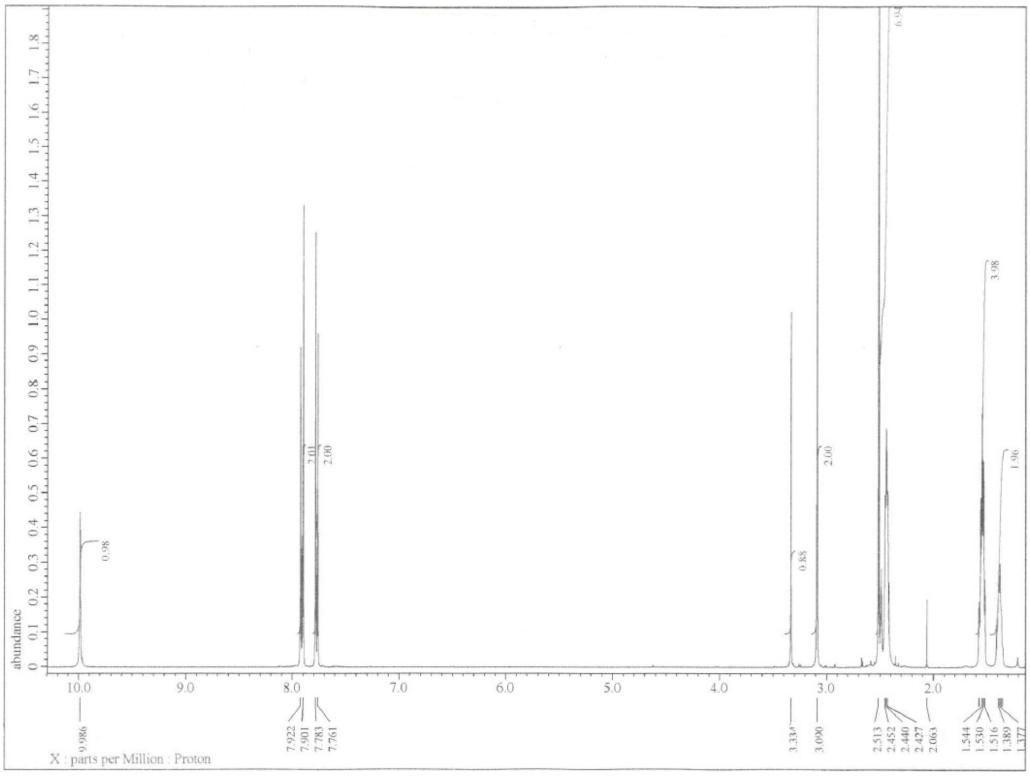

1H-NMR at 400 MHz

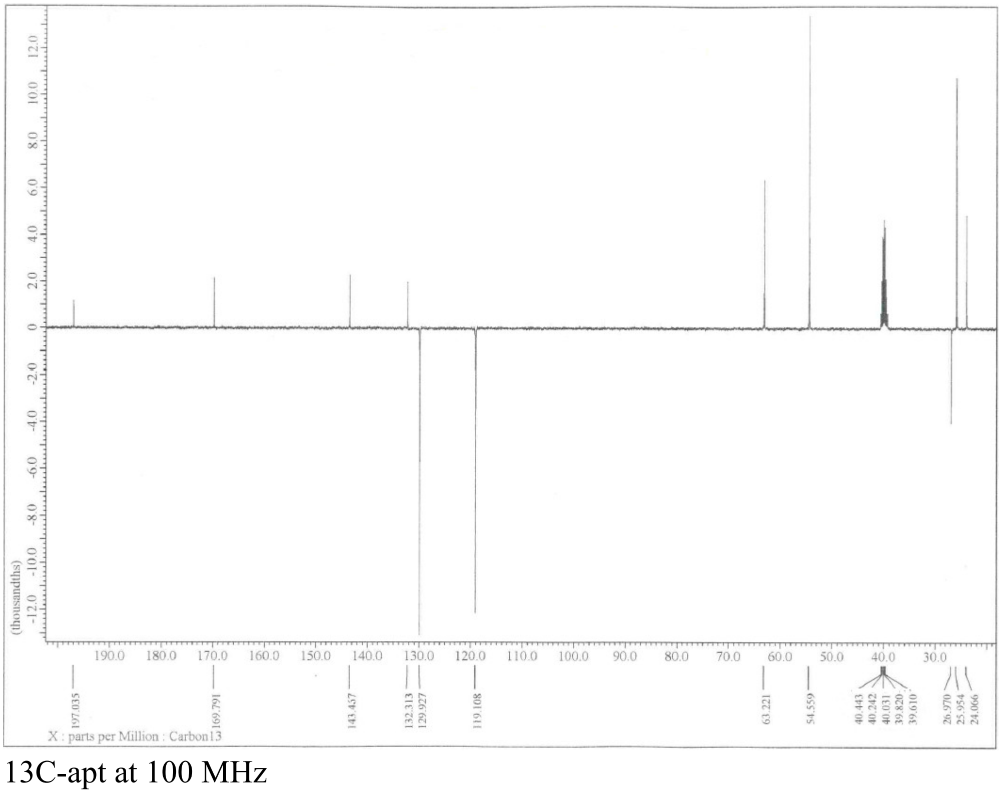

13C-apt at 100 MHz

14-8-1

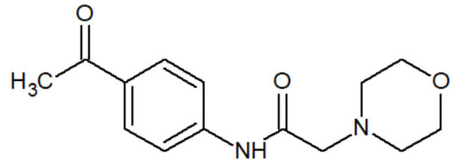

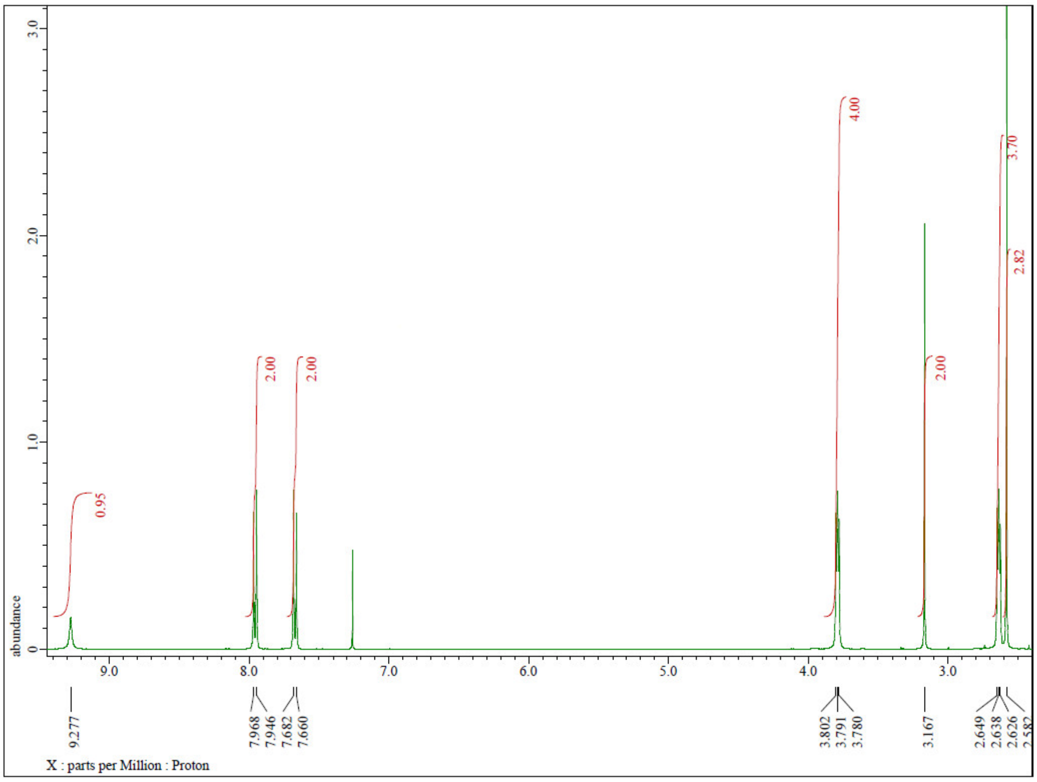

1H-NMR at 400 MHz

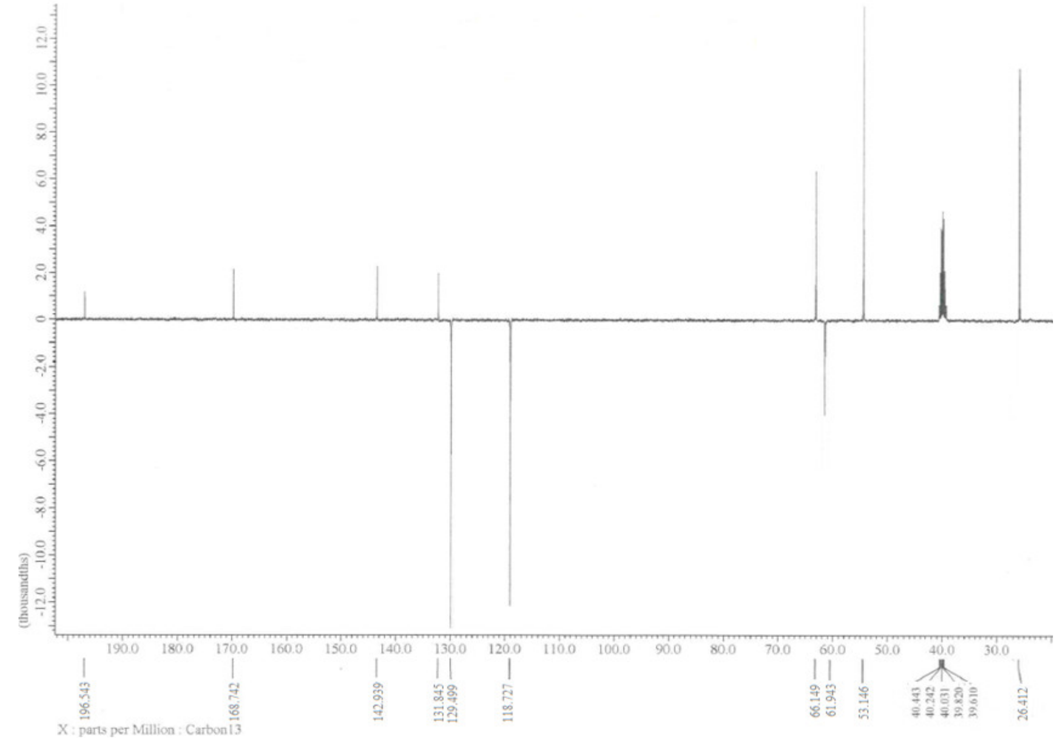

13-C apt at 100 MHz

14-10-1

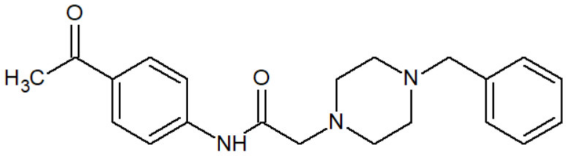

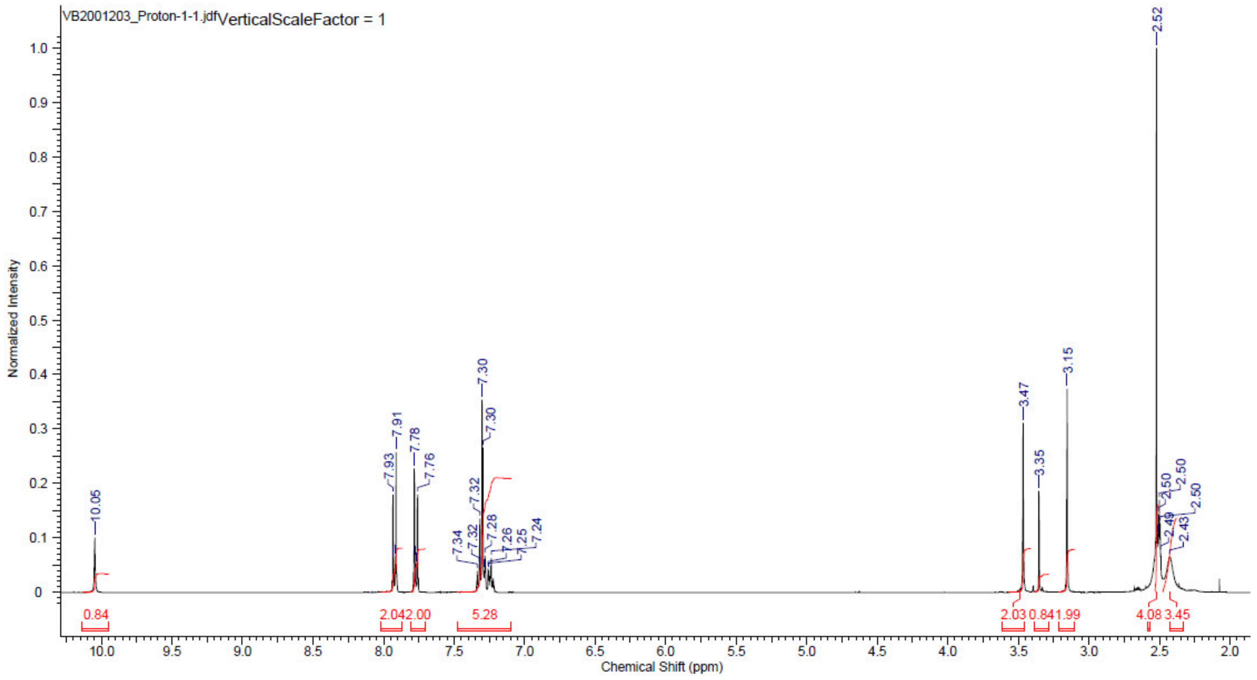

1H-NMR at 400 MHz

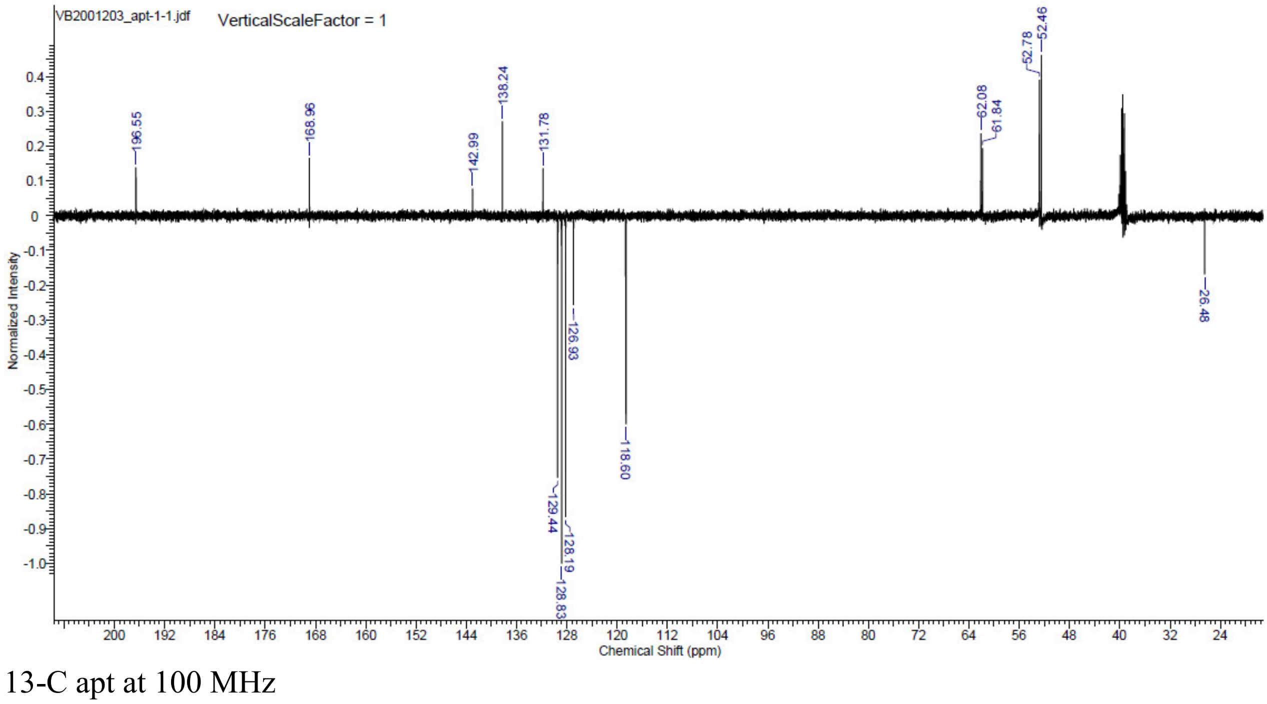

13-C apt at 100 MHz

22-5-1

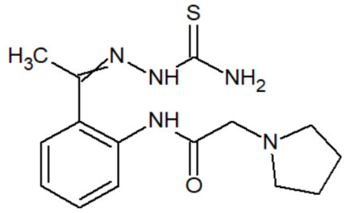

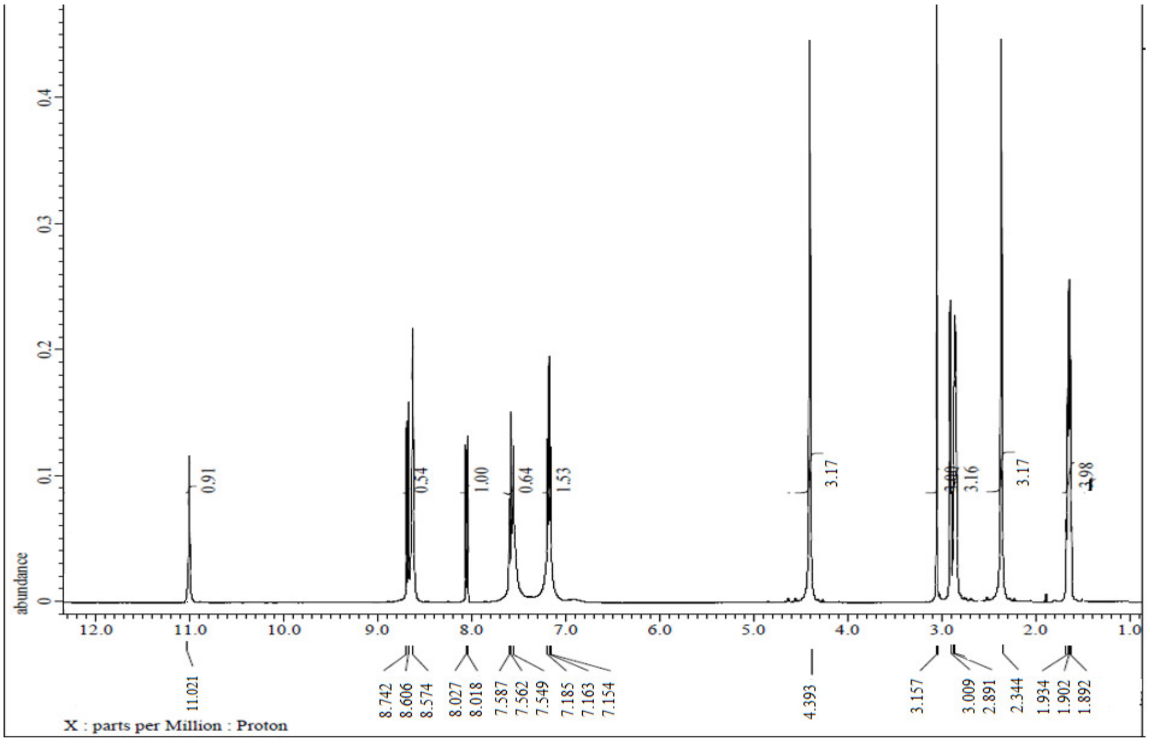

1H-NMR at 400 MHz

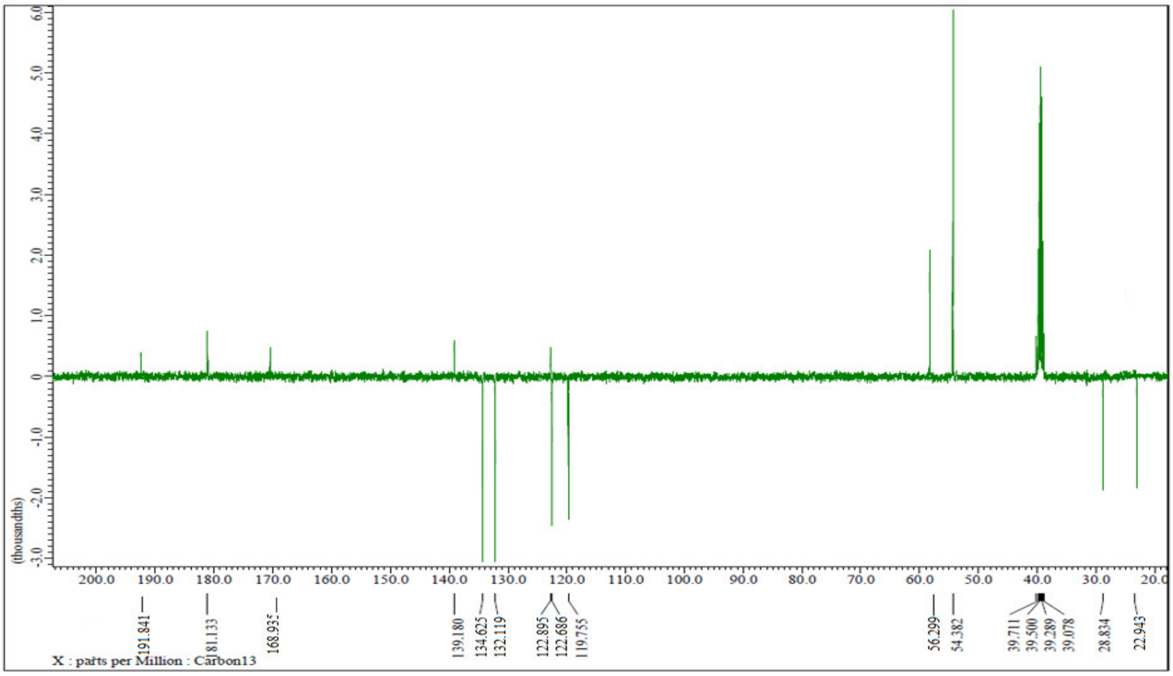

13-C apt at 100 MHz

22-6-1

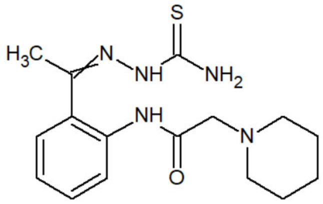

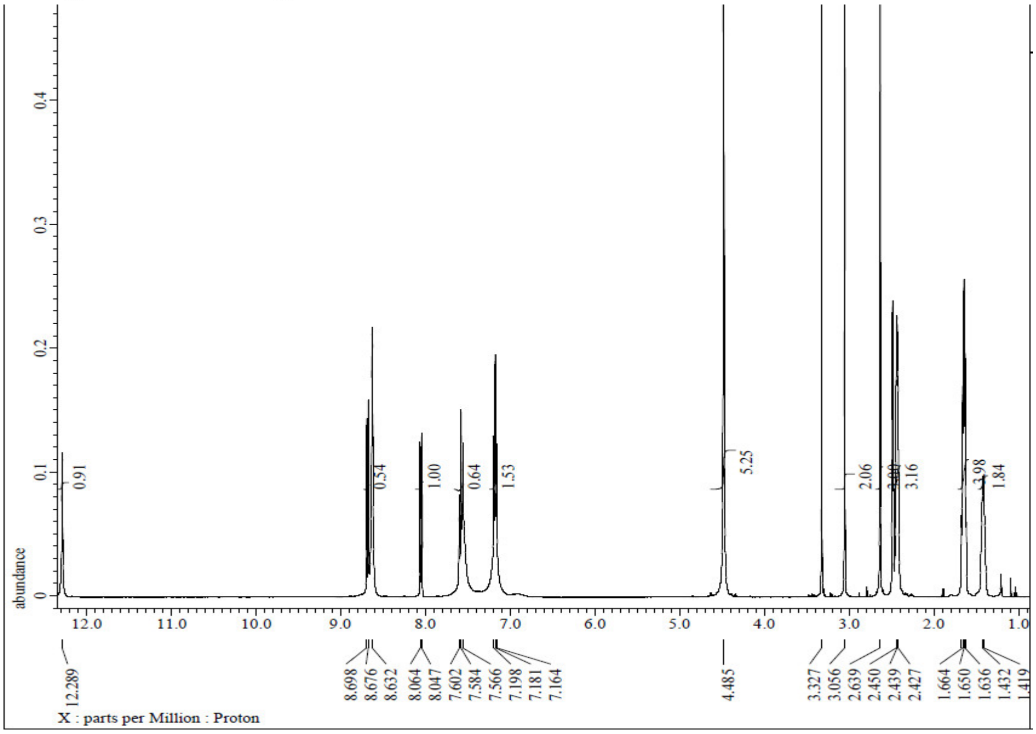

1H-NMR at 400 MHz

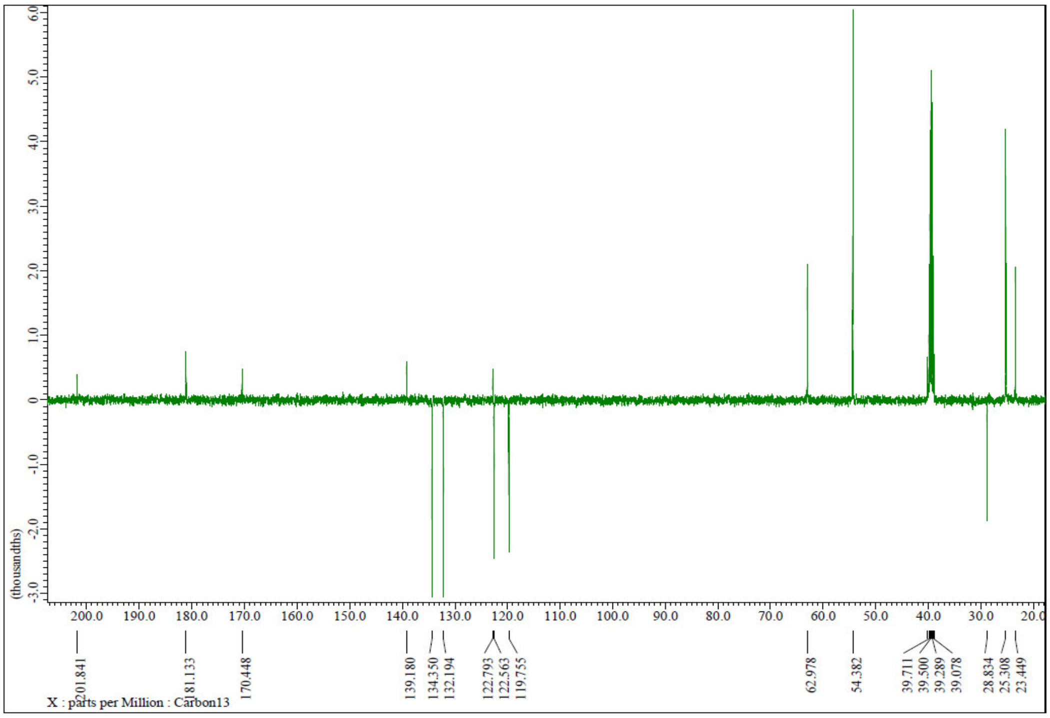

13-C apt at 100 MHz

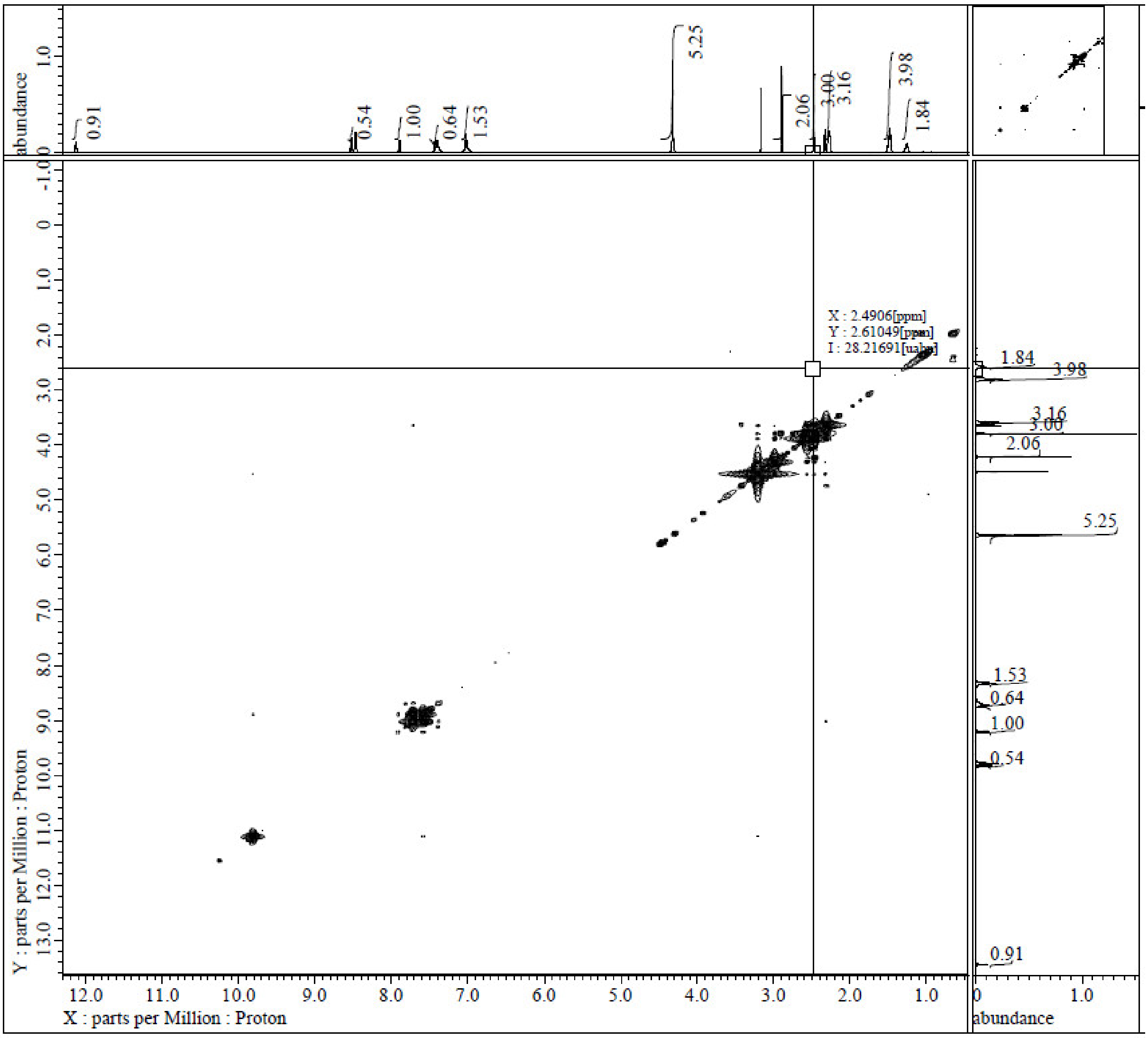
abundance
0.91
0.54
1.00
0.64
1.53
5.25
2.06
3.00
3.16
3.98
1.84
X : 2.4906[ppm]
Y : 2.61049[ppm]
I : 28.21691[uabn]
Y : parts per Million : Proton
X : parts per Million : Proton
abundance

H-H cosy (2D)

22-7-1

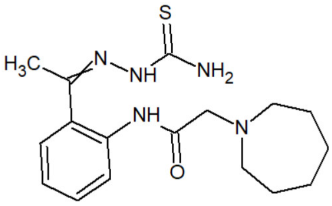

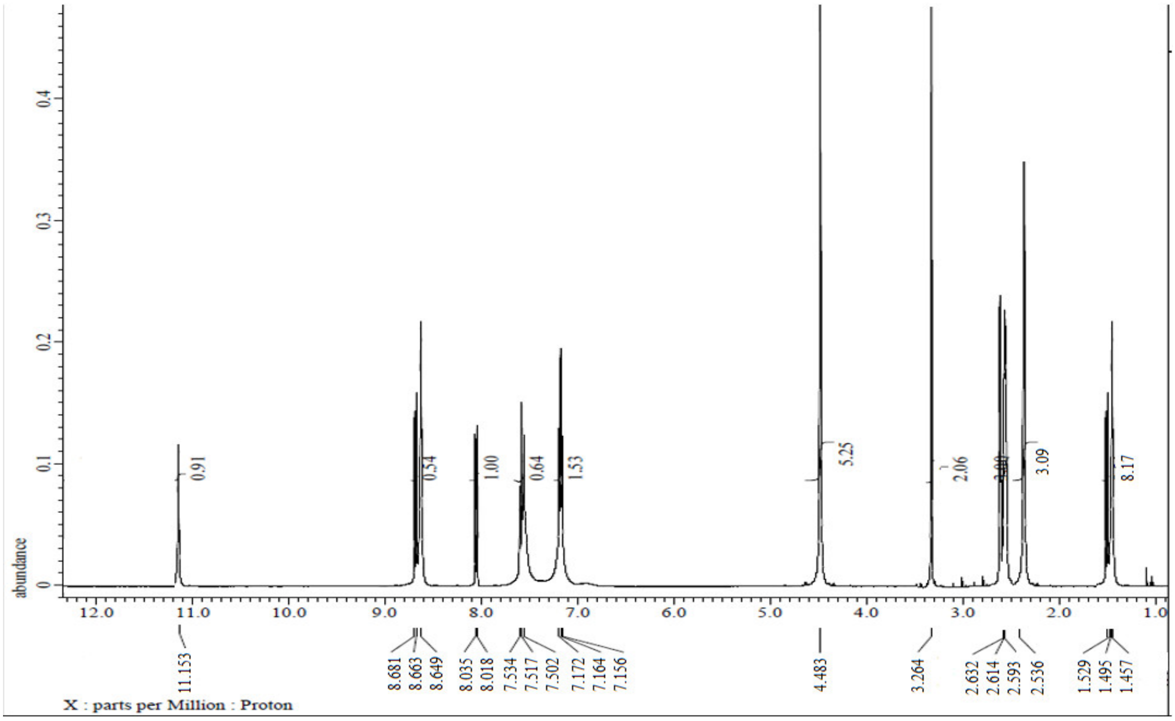

1H-NMR at 400 MHz

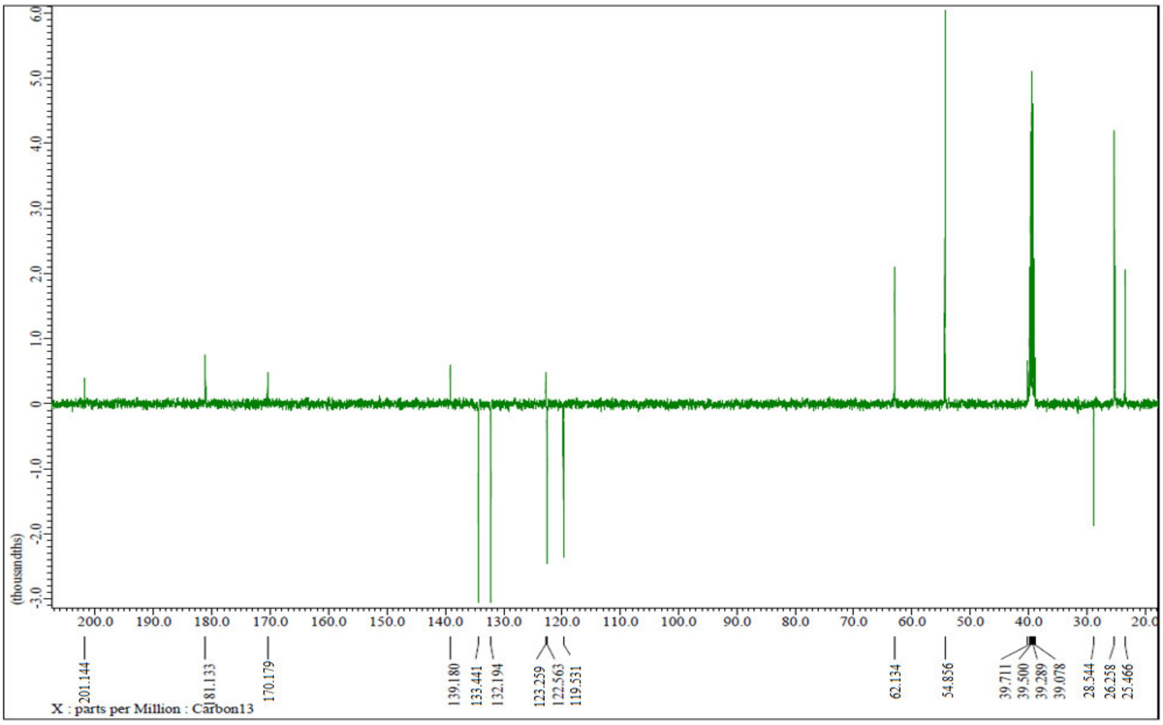

13-C apt at 100 MHz

23-2-1

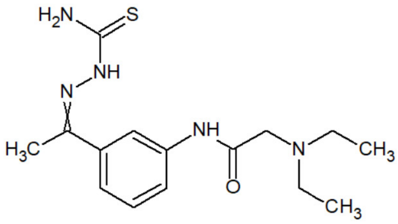

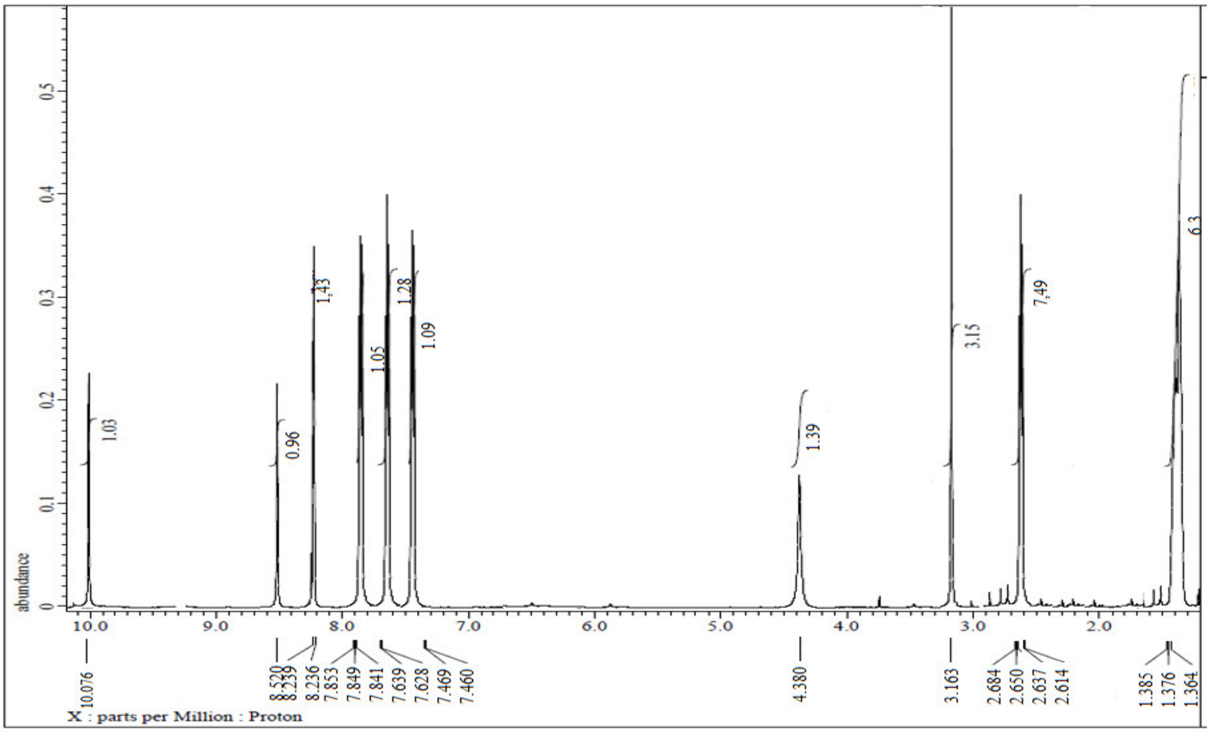

1H-NMR at 400 MHz

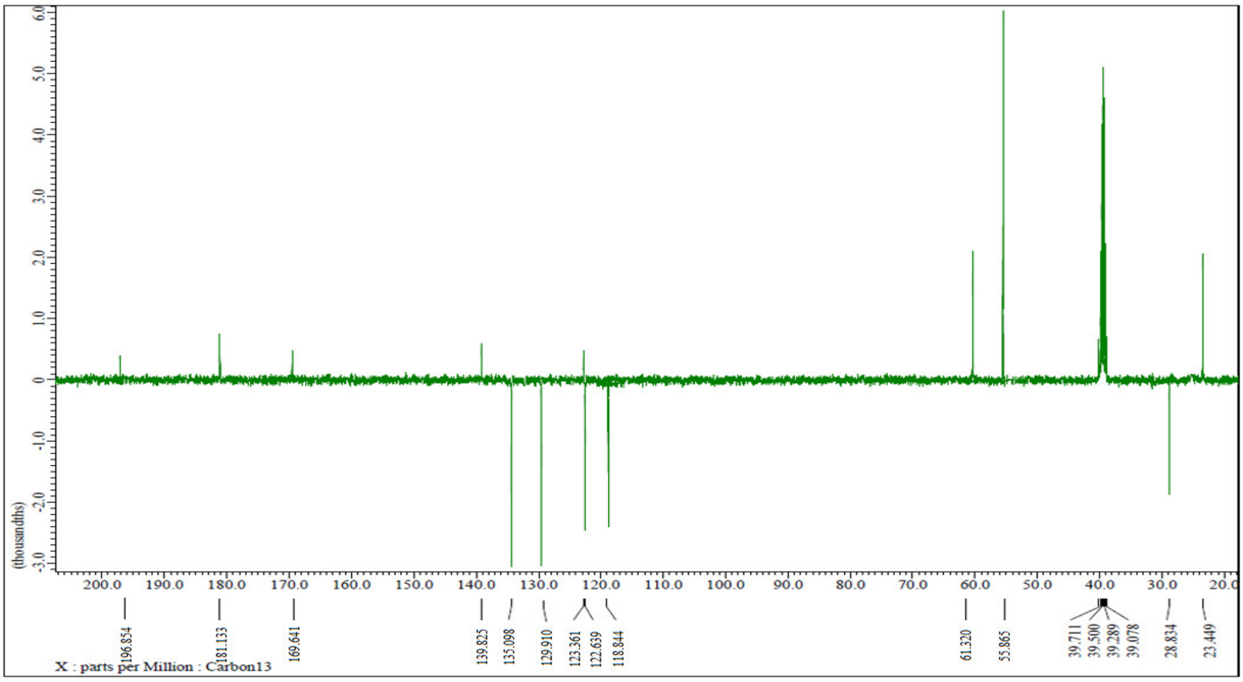

13-C apt at 100 MHz

23-3-1

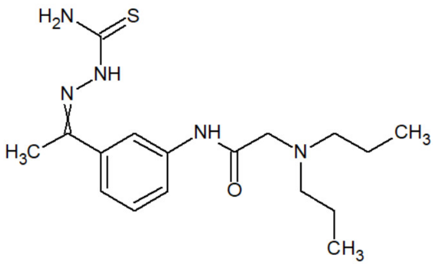

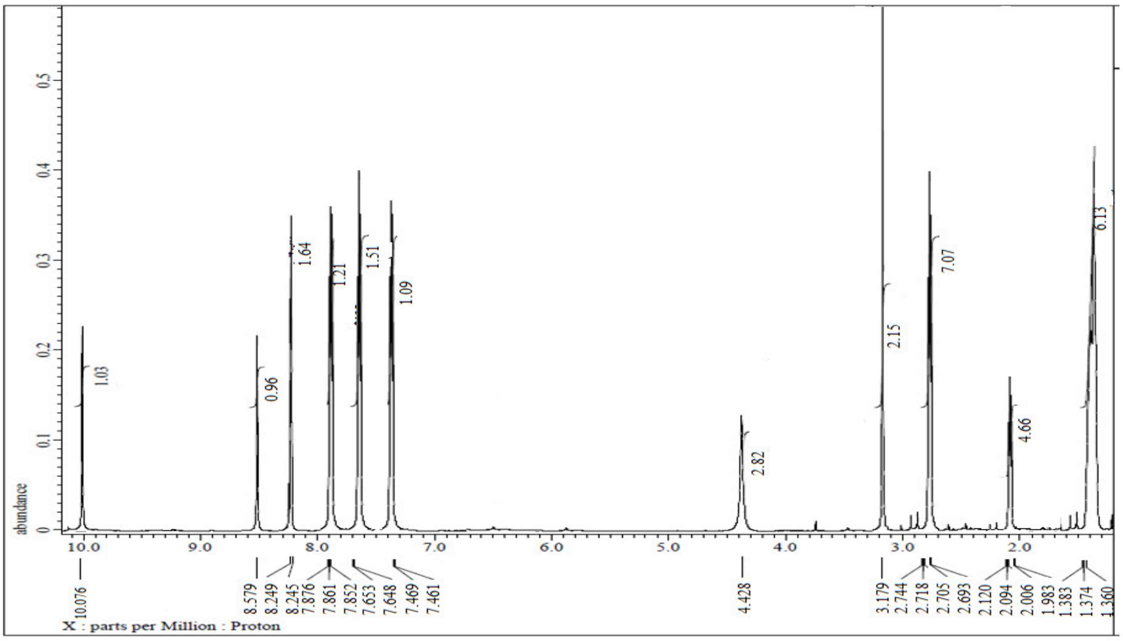

1H-NMR at 400 MHz

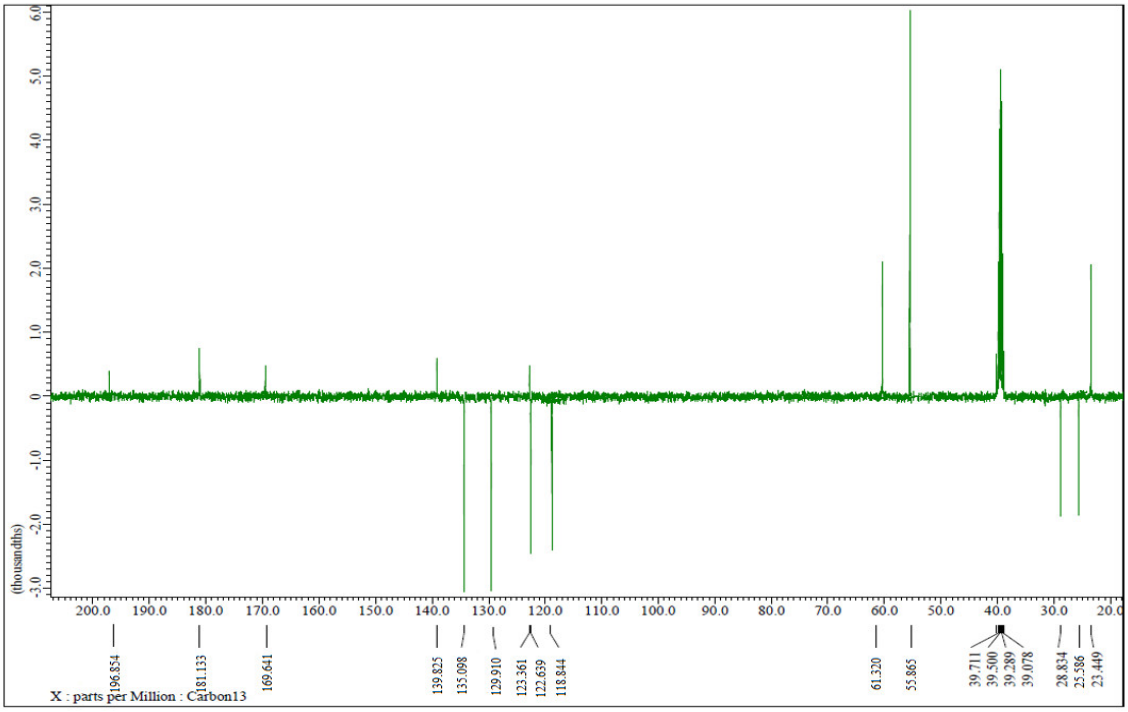

13-C apt at 100 MHz

23-5-1

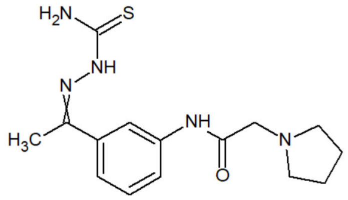

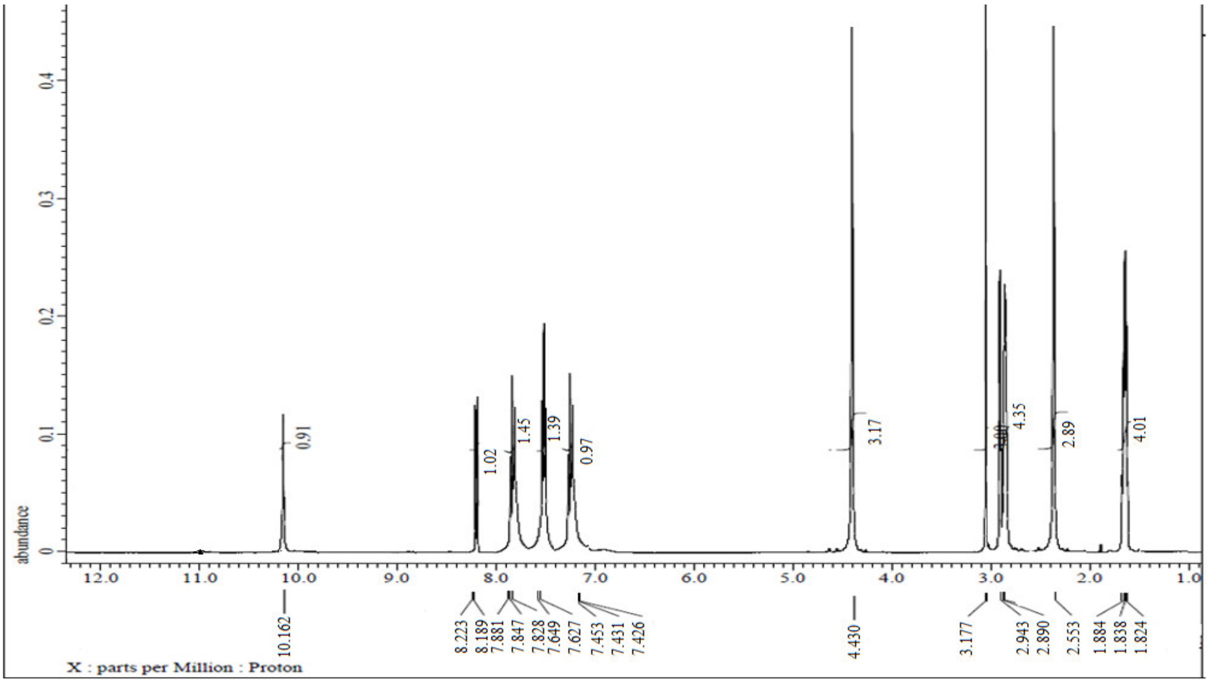

1H-NMR at 400 MHz

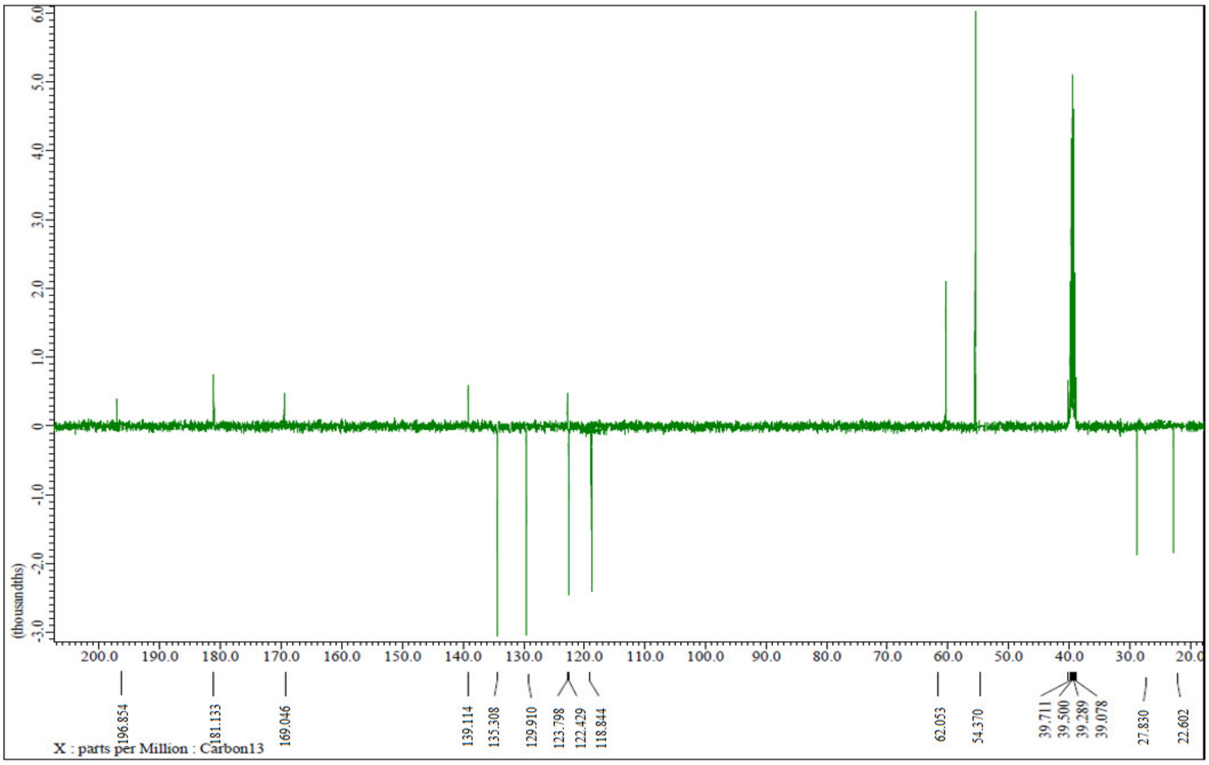

13-C apt at 100 MHz

23-6-1

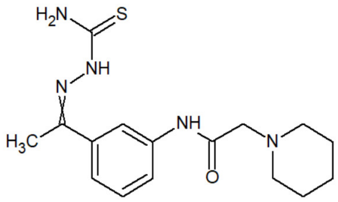

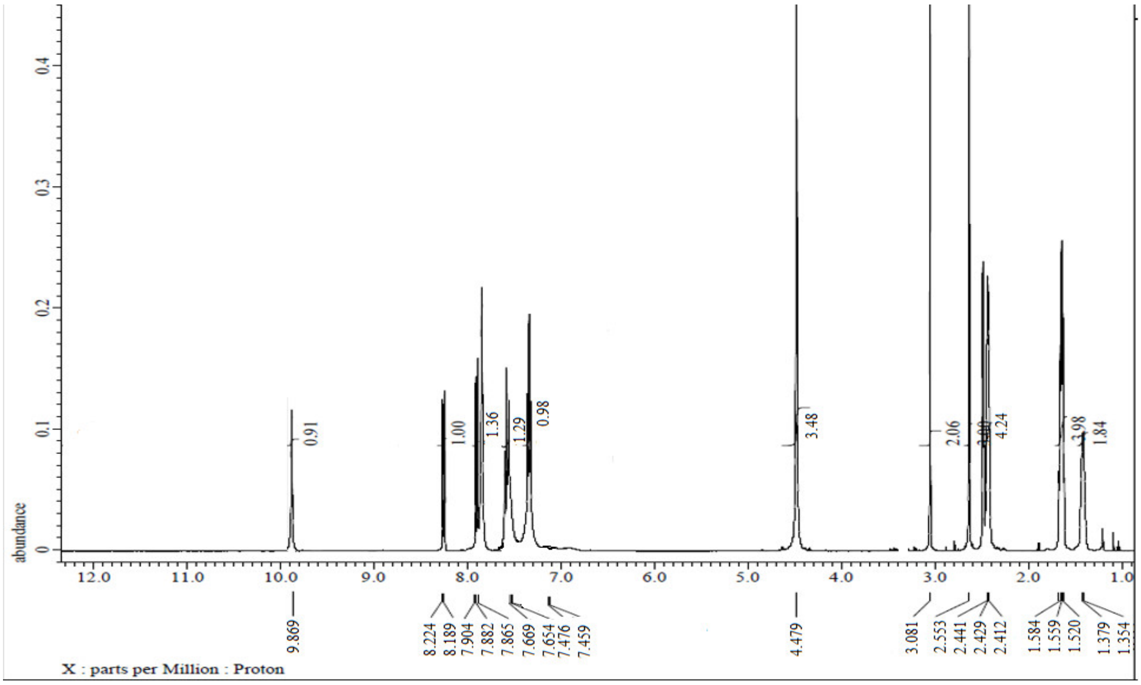

1H-NMR at 400 MHz

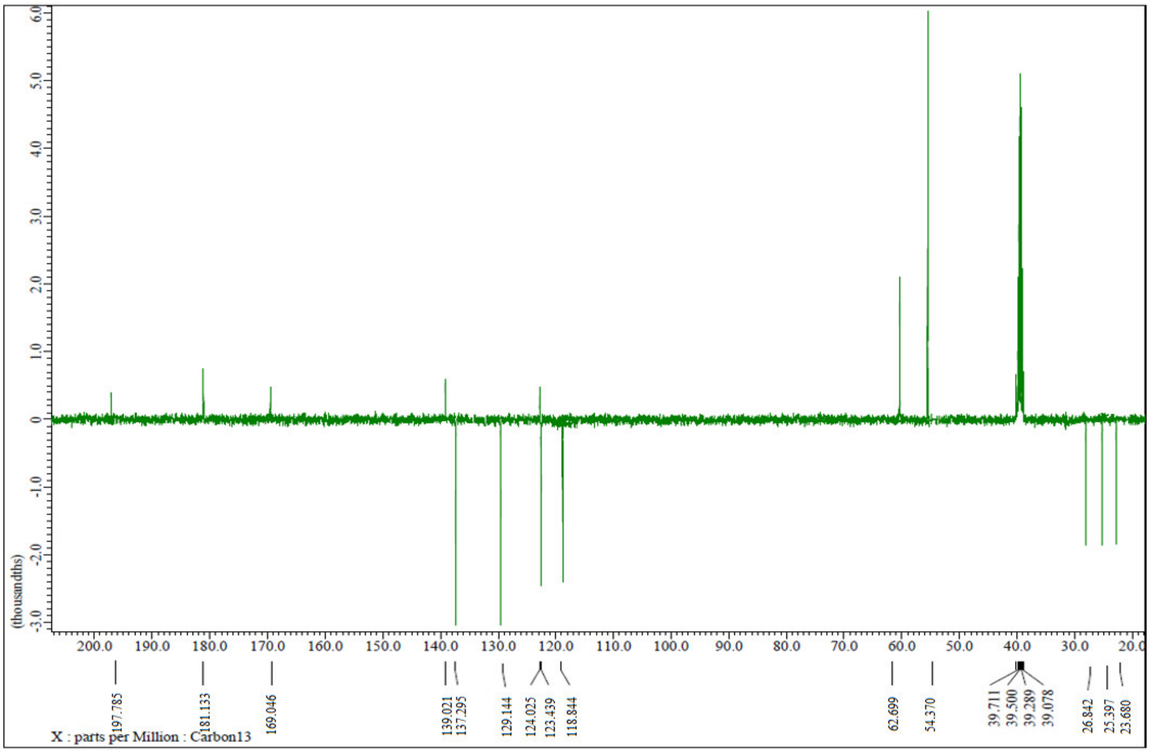

13-C apt at 100 MHz

23-8-1

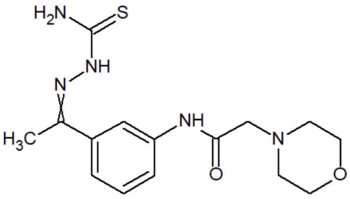

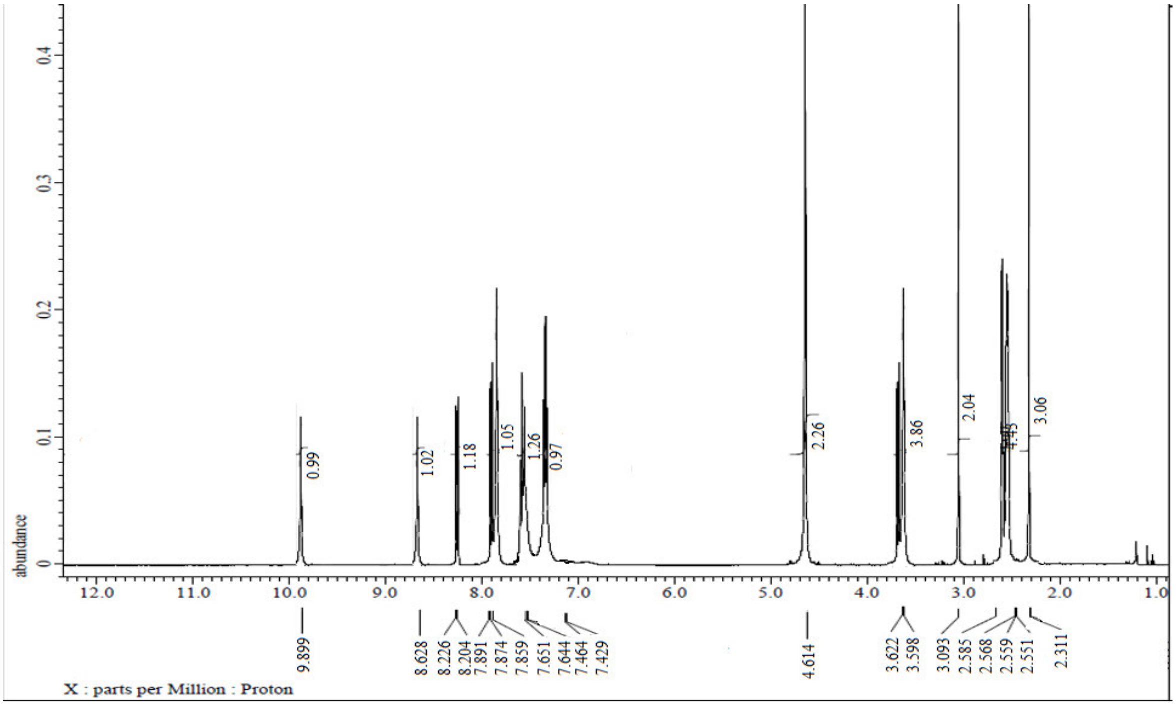

1H-NMR at 400 MHz

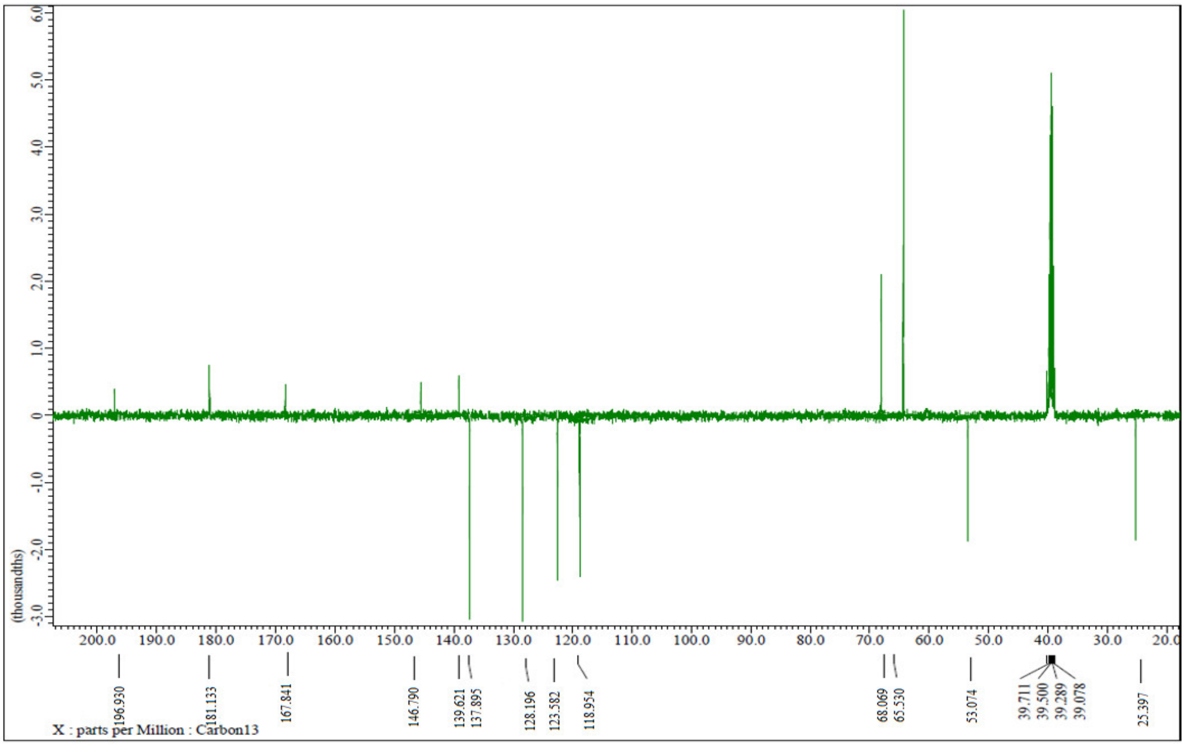

13-C apt at 100 MHz

23-11-1

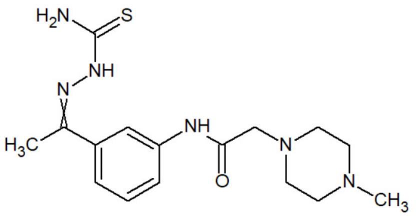

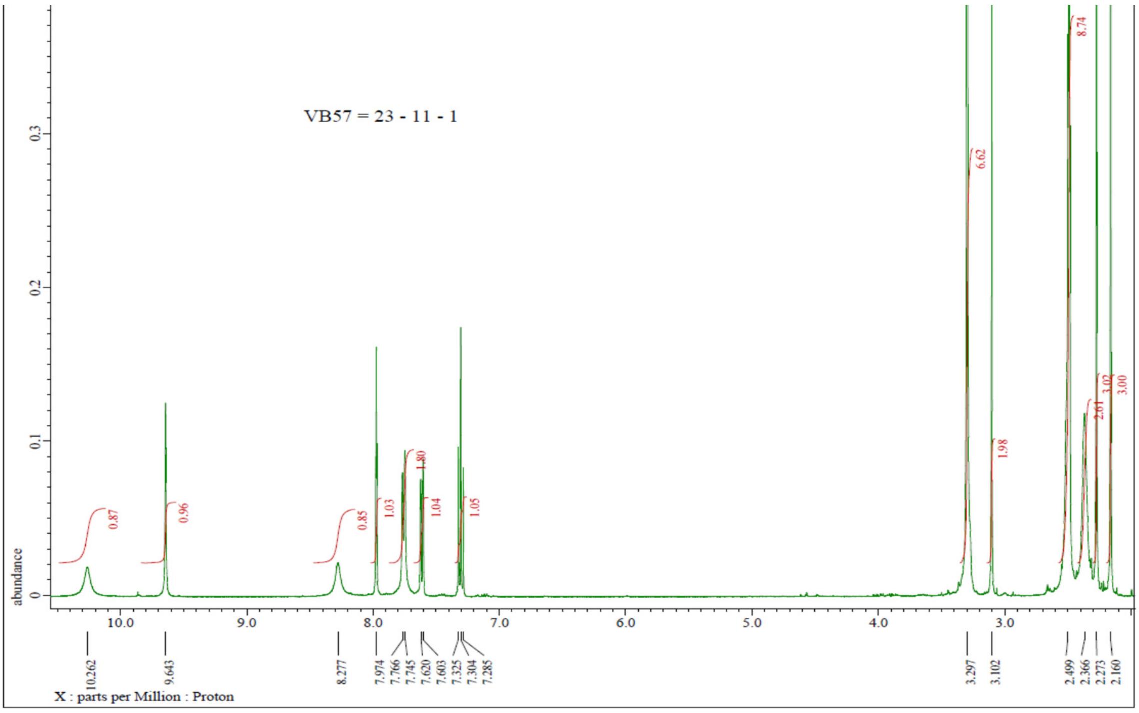

1H-NMR at 400 MHz

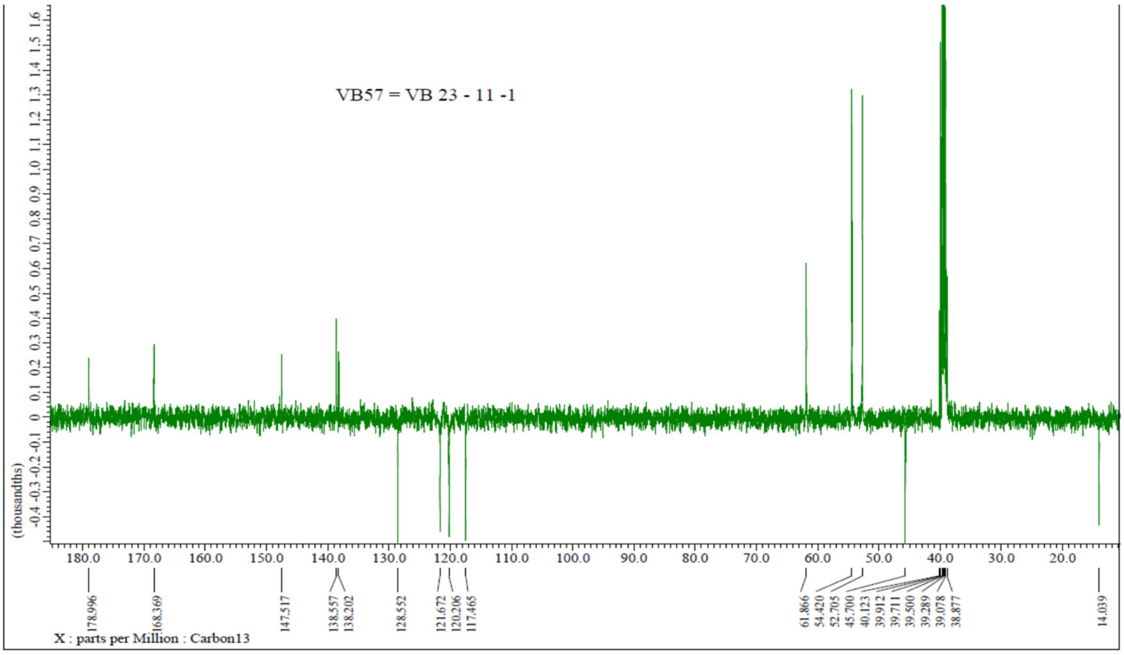

13-C apt at 100 MHz

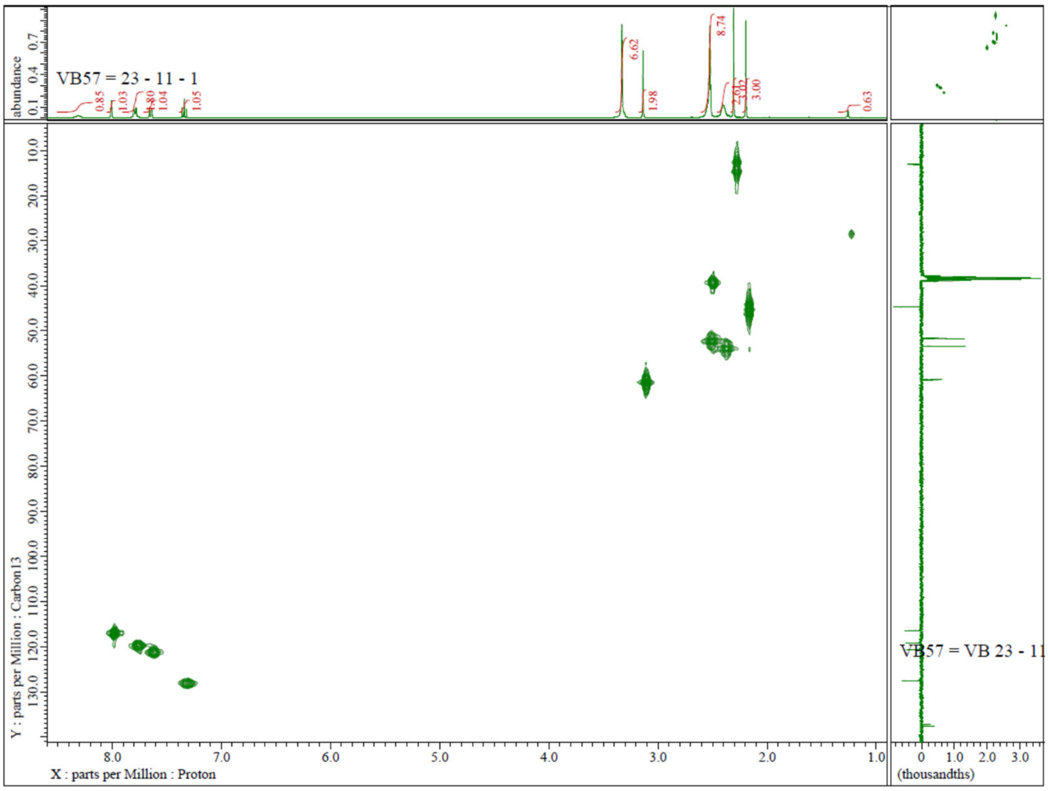

HMQC (2D C-H spectrum)

24-3-1

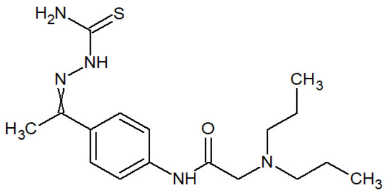

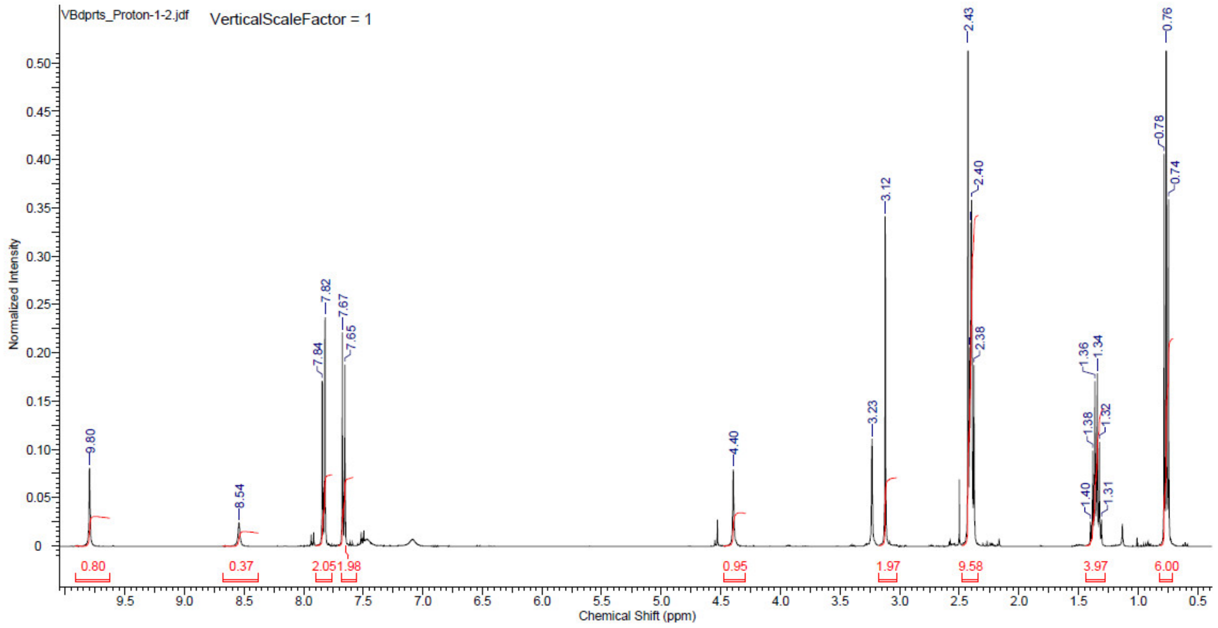

1-H NMR at 400 MHz

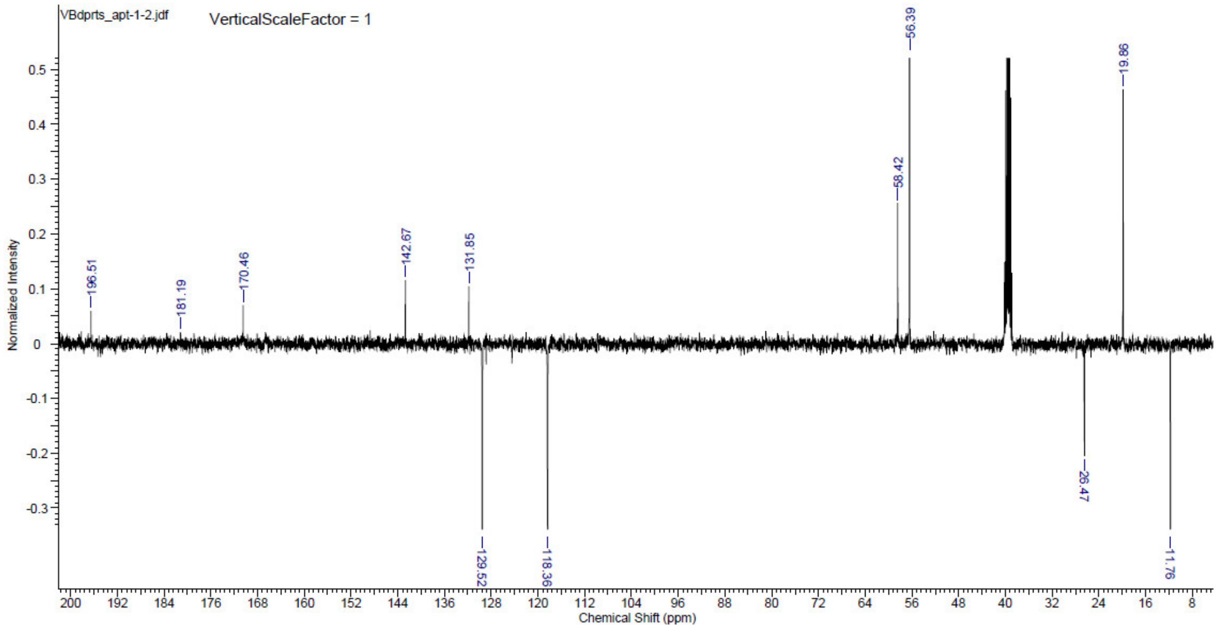

13C-apt at 100 MHz

24-4-1

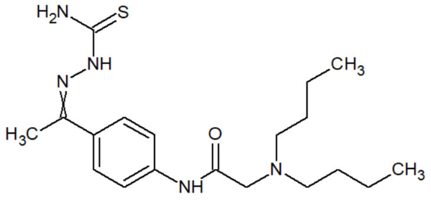

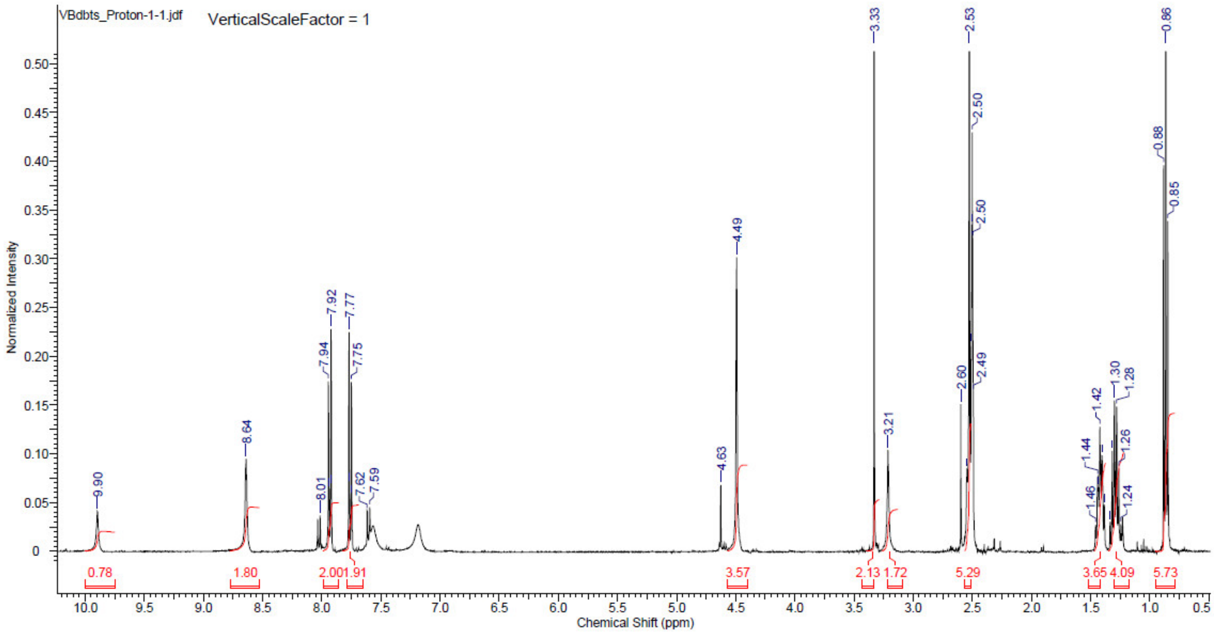

1-H NMR at 400 MHz

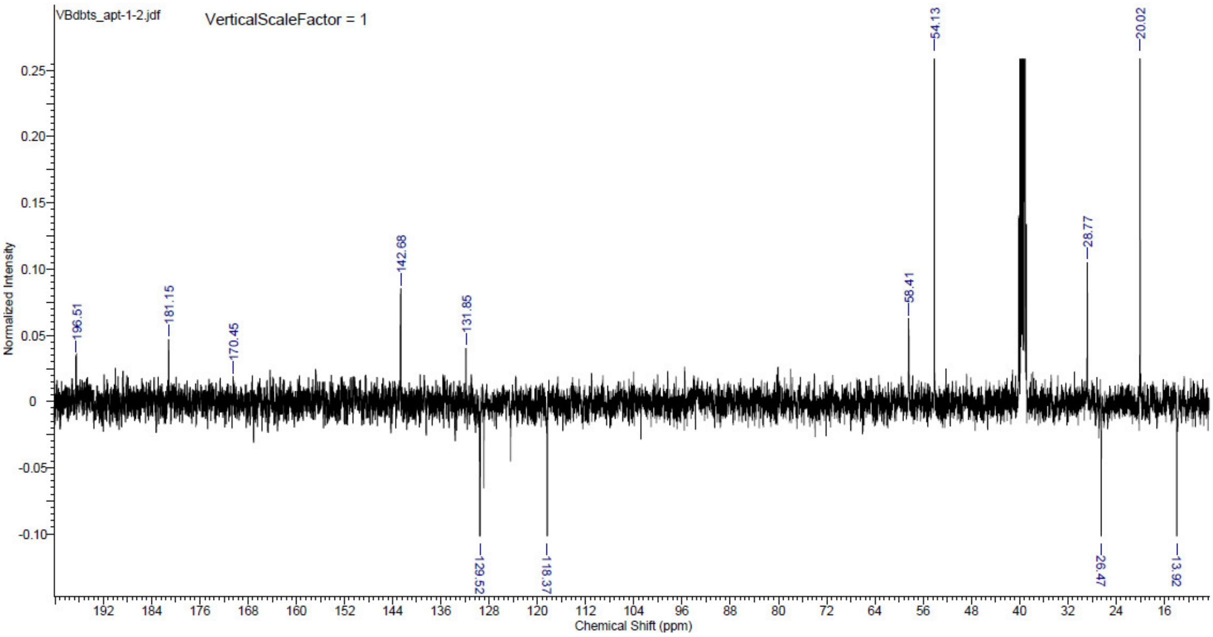

13C-apt at 100 MHz

24-5-1

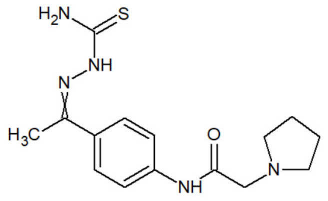

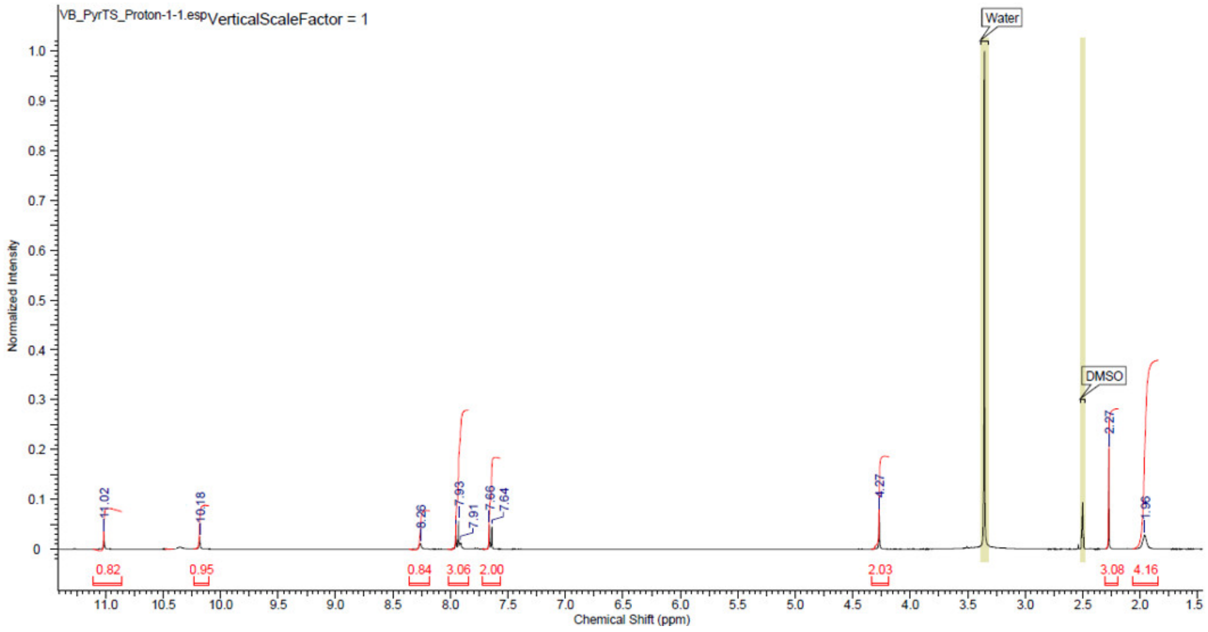

1-H NMR at 400 MHz

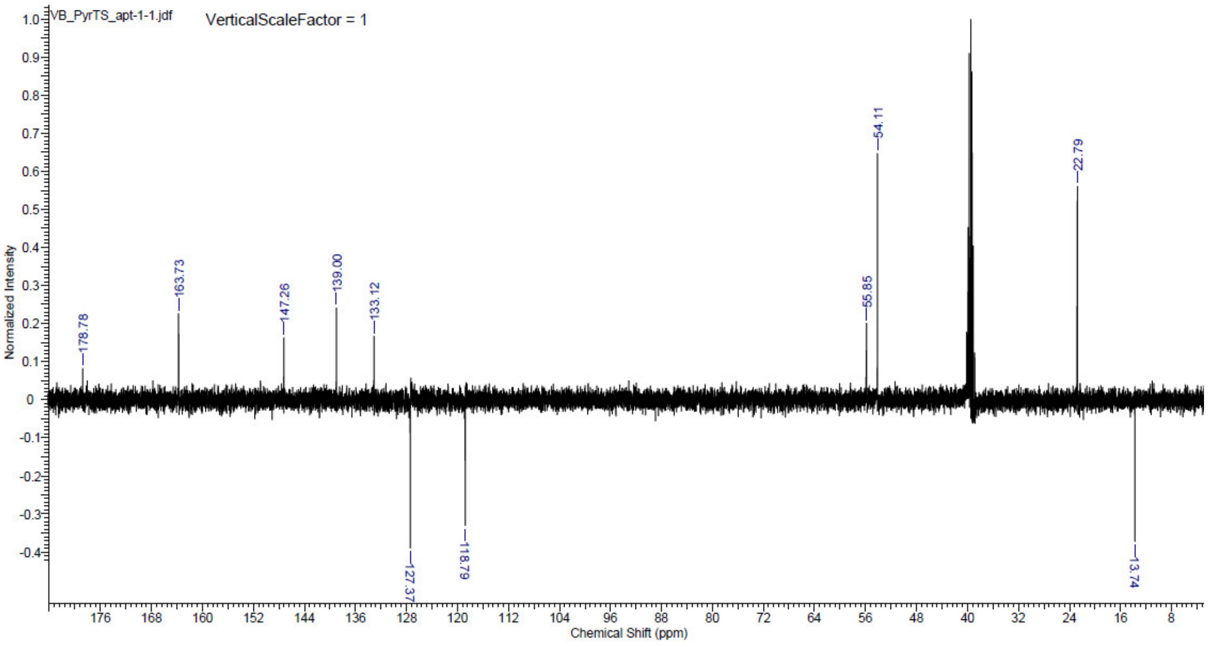

13C-apt at 100 MHz

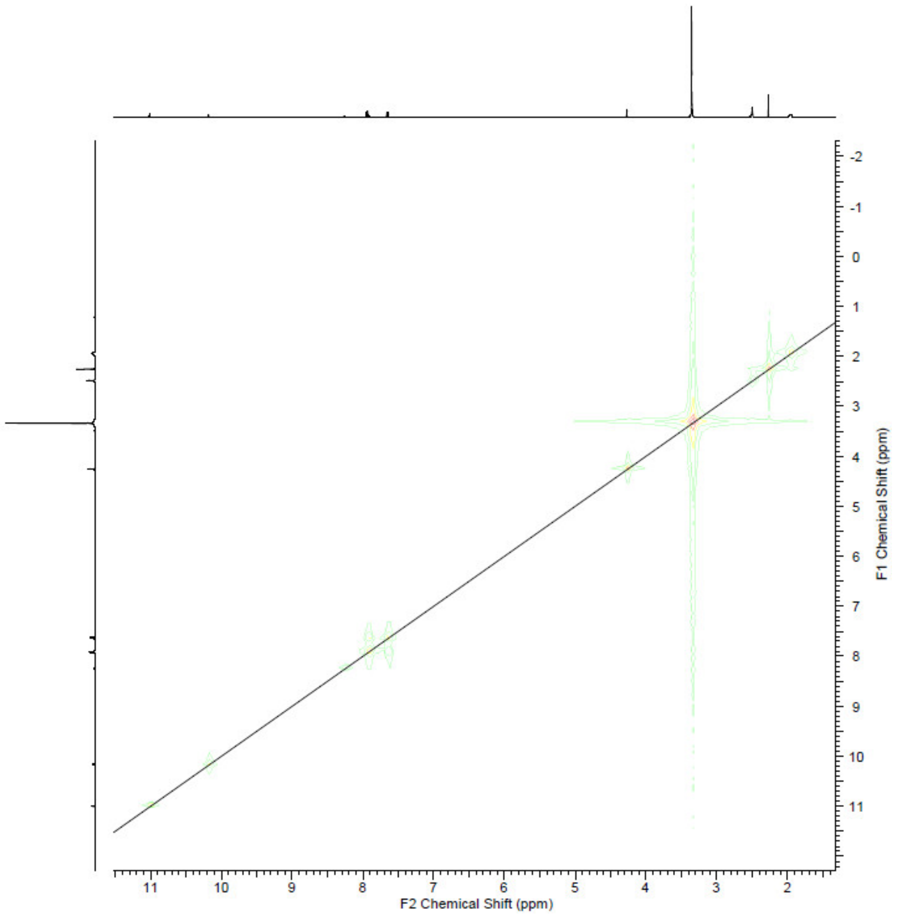

H-H cosy (2D)

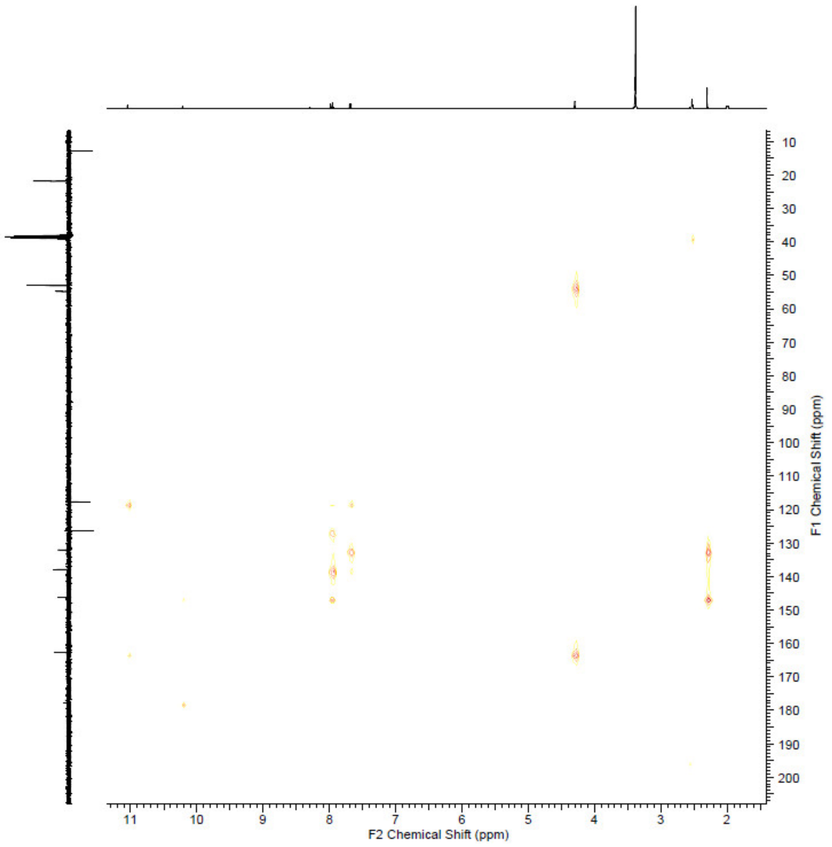

HMBC

24-6-1

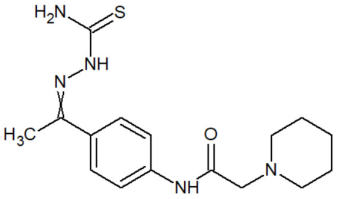

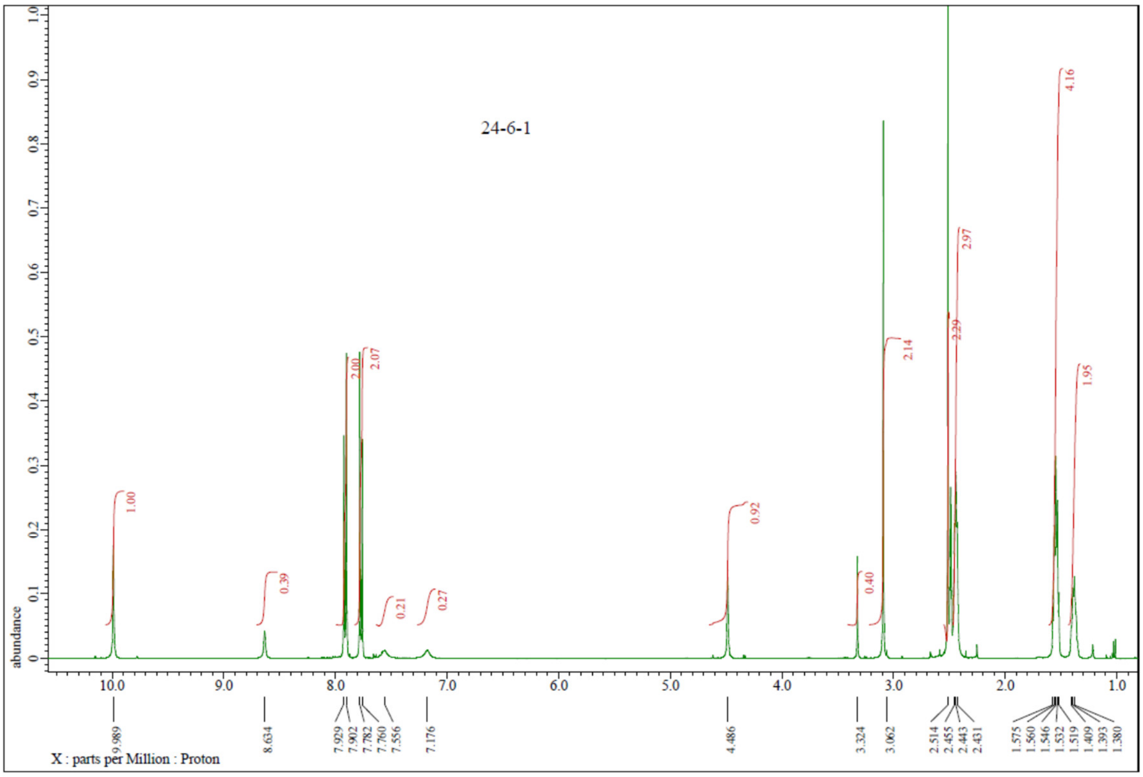

1-H NMR at 400 MHz

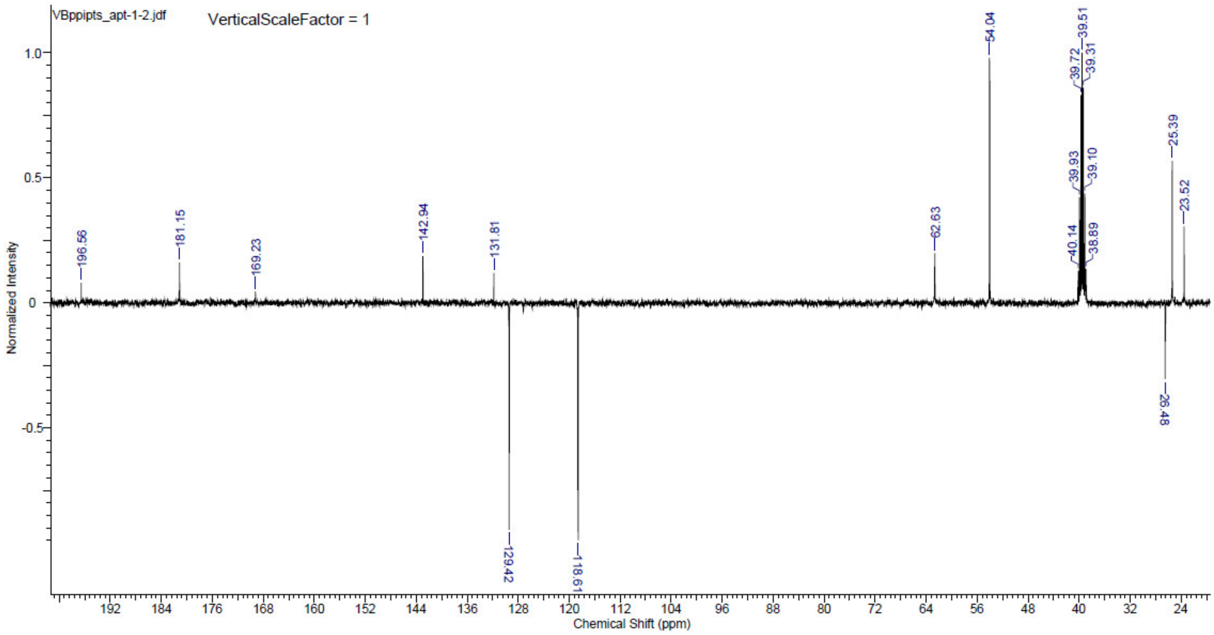

13C-apt at 100 MHz

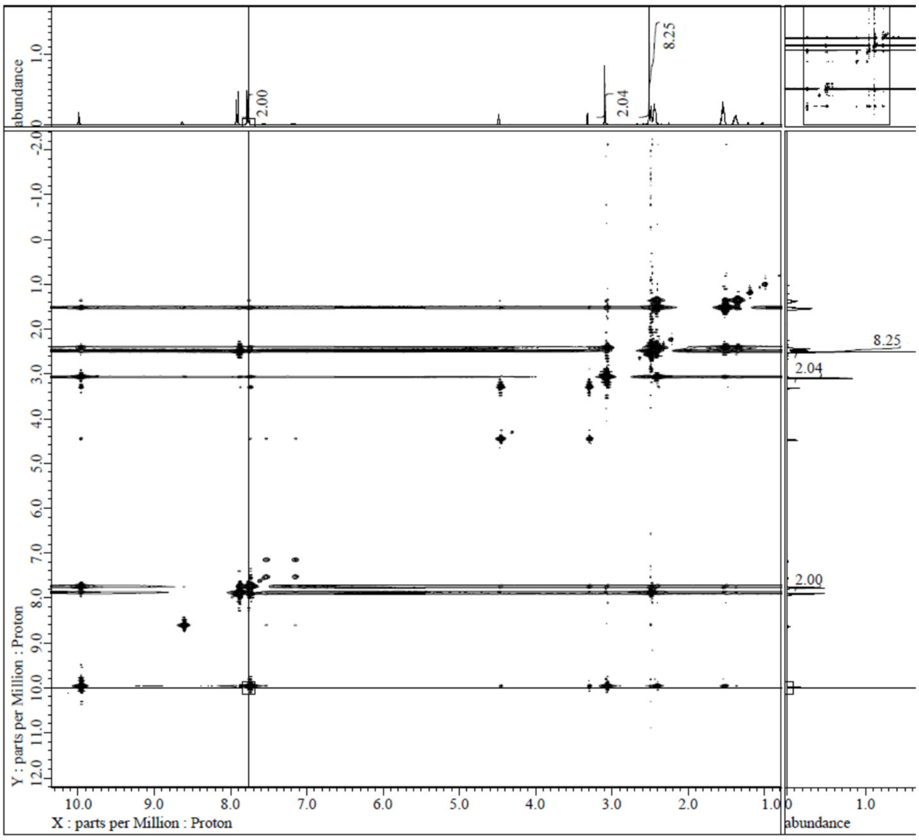

H-H NOESY for determination of *E/Z* geometry: approx. equimolar ratio

24-7-1

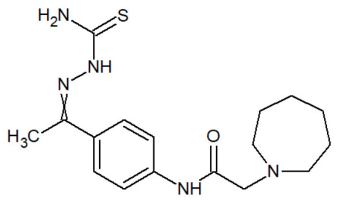

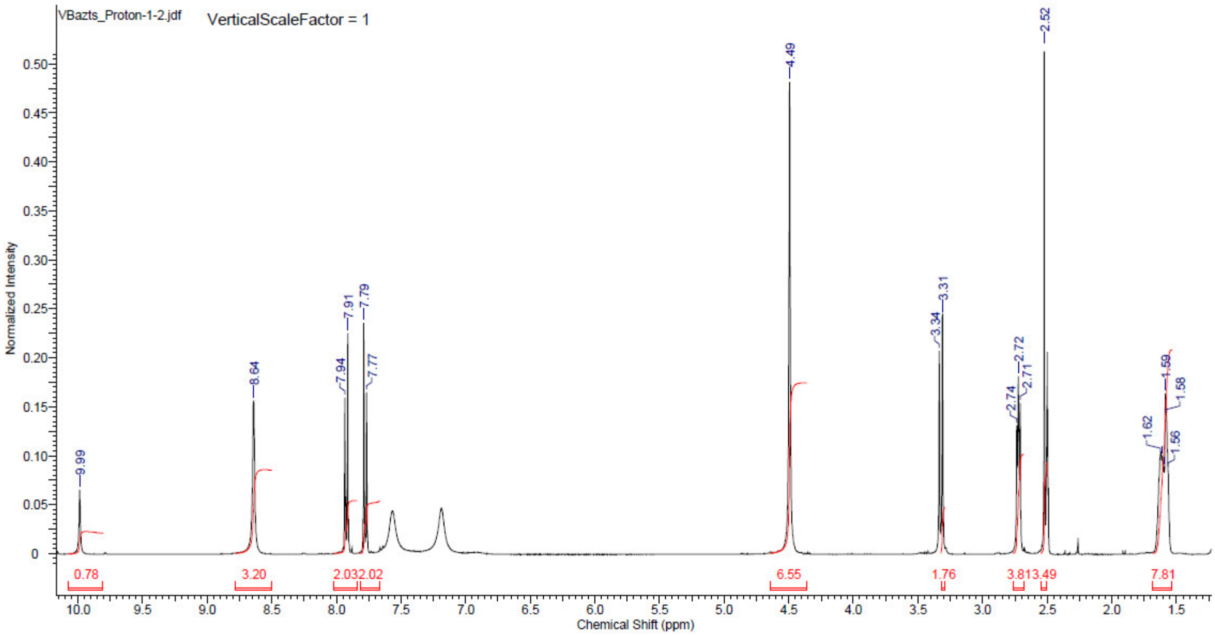

1-H NMR at 400 MHz

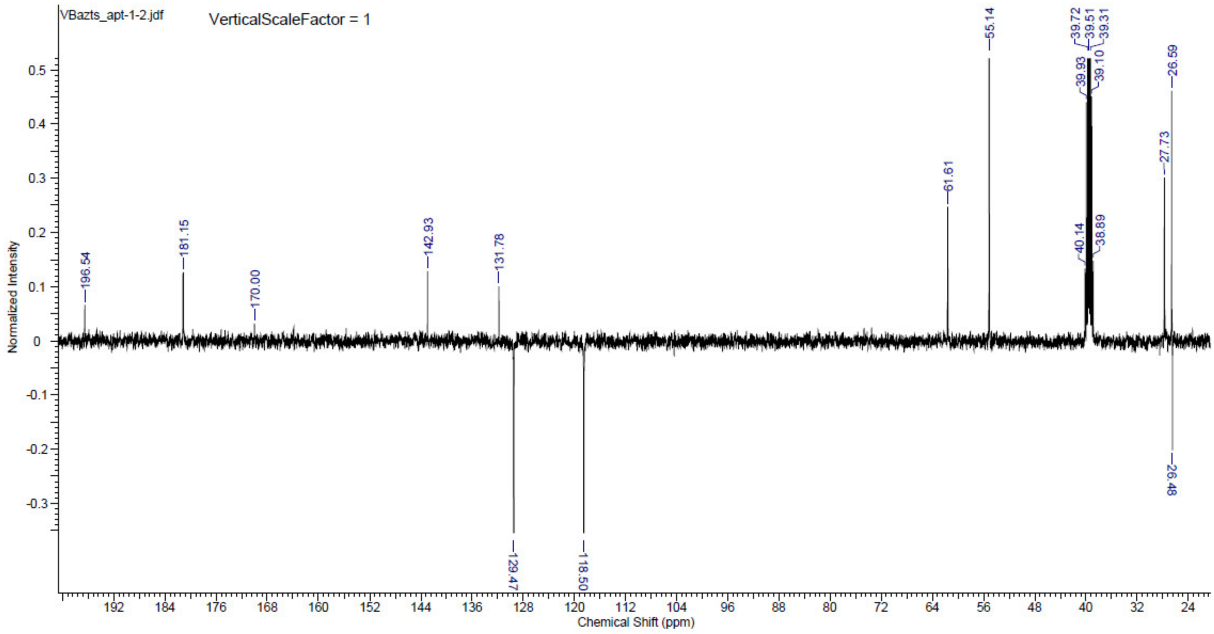

13C-apt at 100 MHz

24-8-1

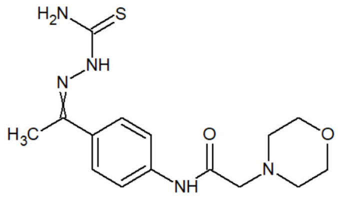

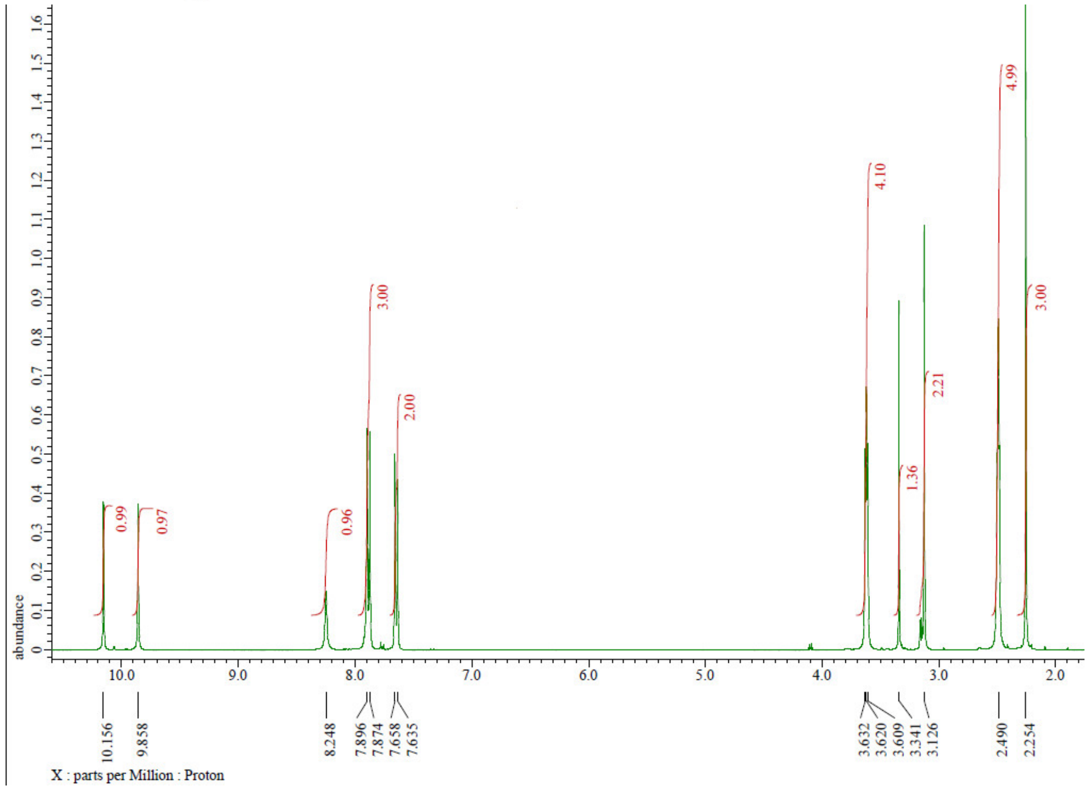

1-H NMR at 400 MHz

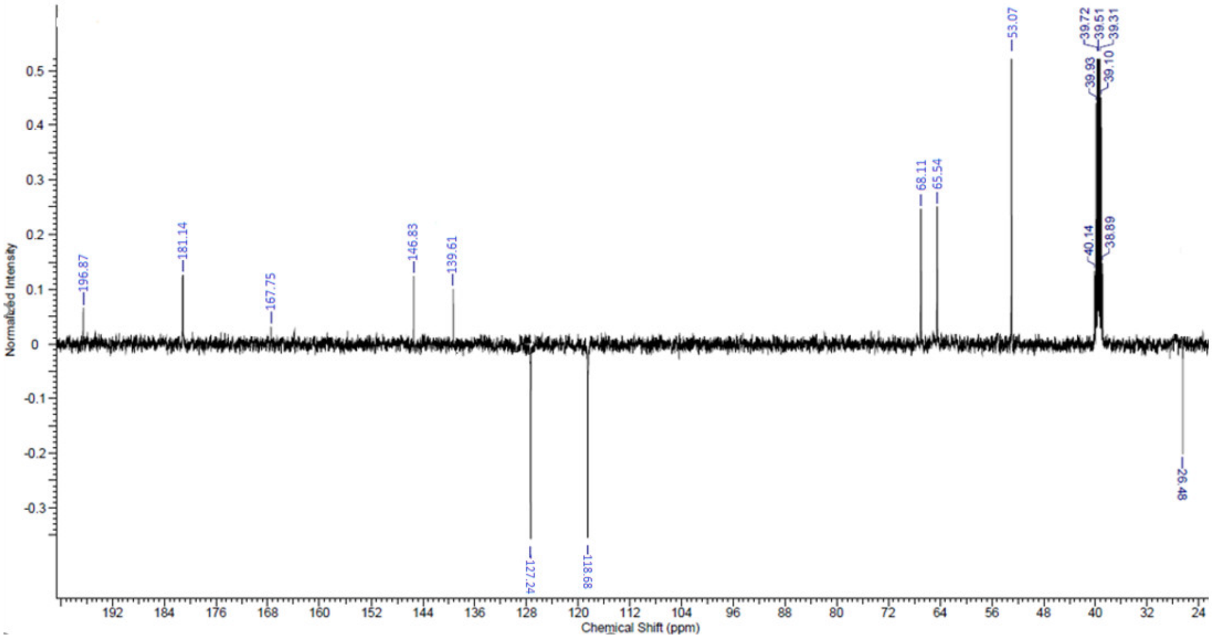

13C-apt at 100 MHz

24-9-1

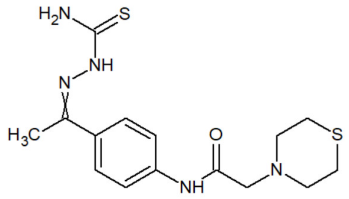

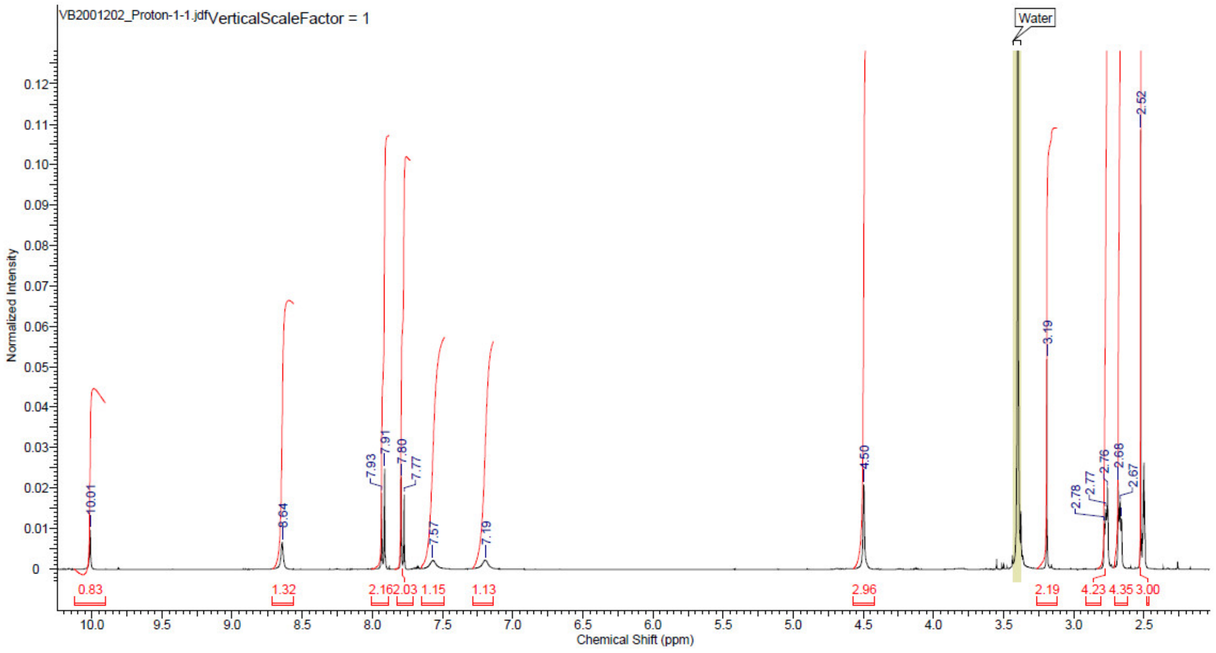

1-H NMR at 400 MHz

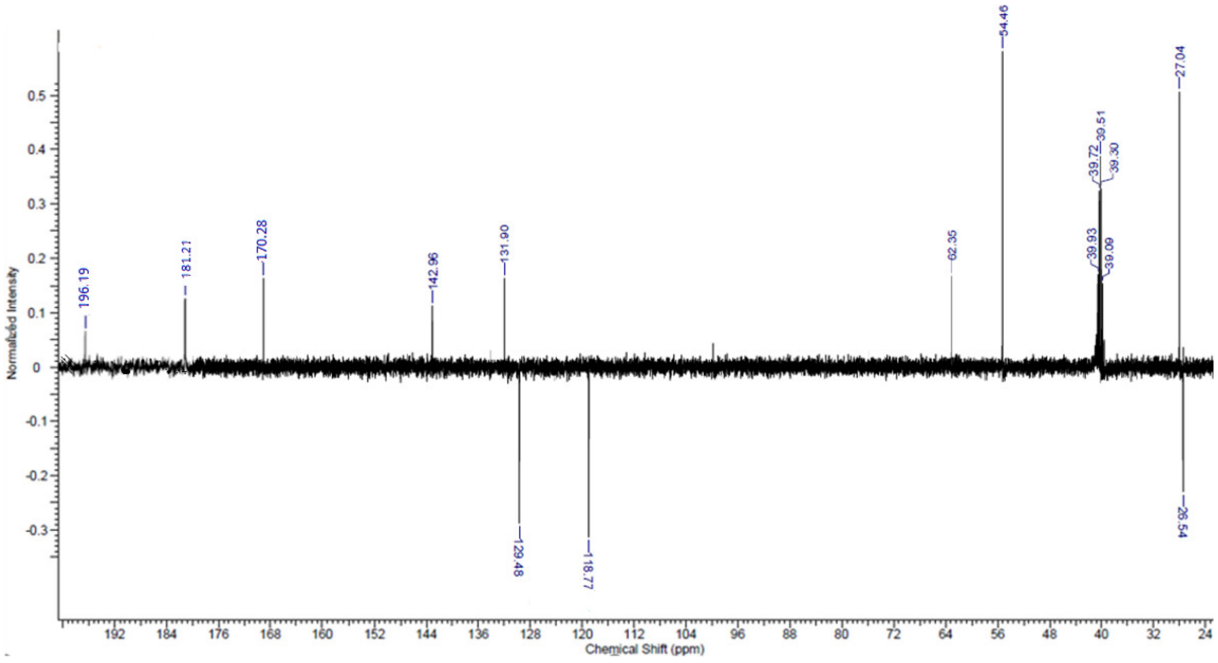

13C-apt at 100 MHz

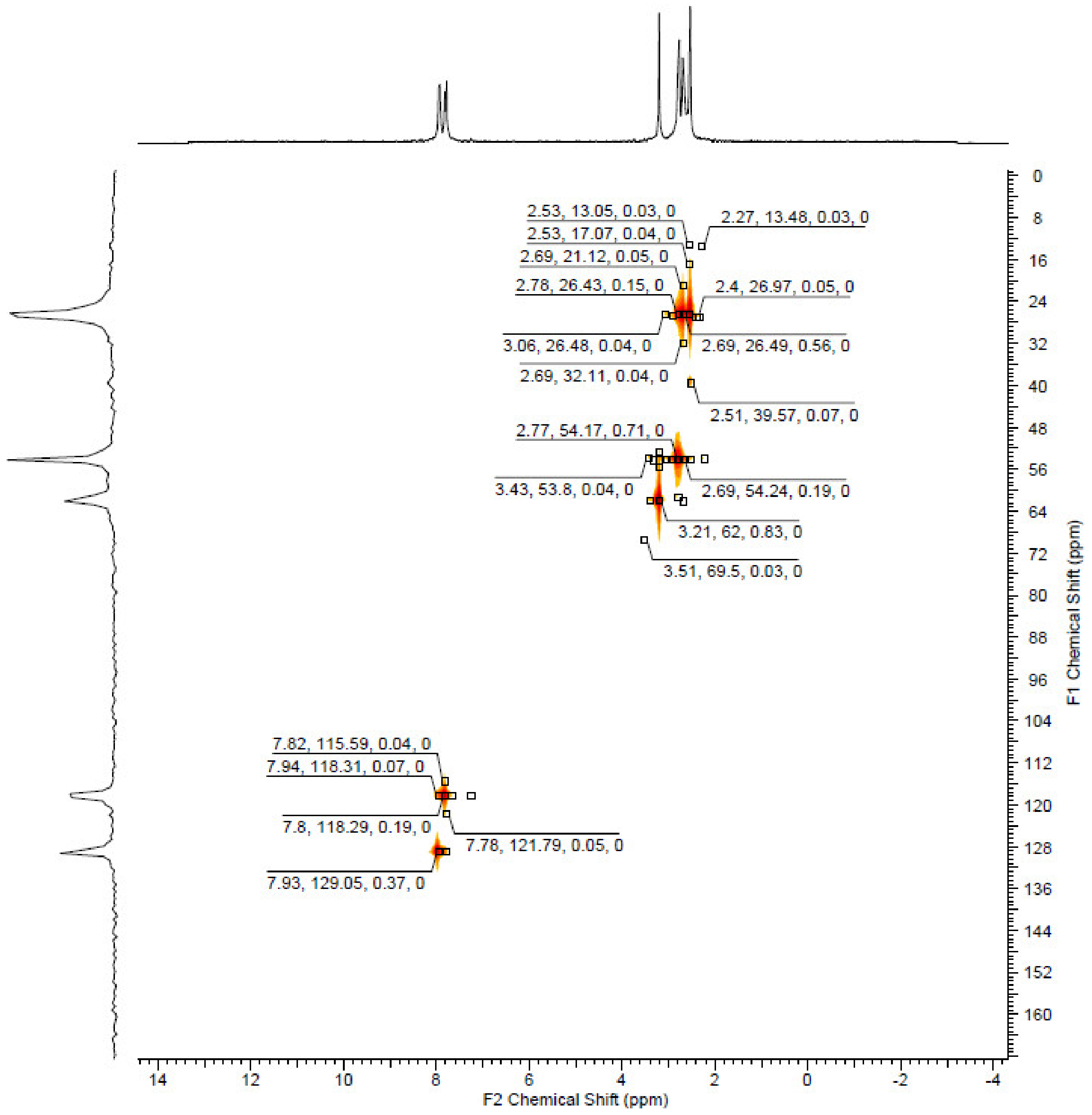

2.53, 13.05, 0.03, 0
2.27, 13.48, 0.03, 0
2.53, 17.07, 0.04, 0
2.69, 21.12, 0.05, 0
2.78, 26.43, 0.15, 0
2.4, 26.97, 0.05, 0
3.06, 26.48, 0.04, 0
2.69, 26.49, 0.56, 0
2.69, 32.11, 0.04, 0
2.51, 39.57, 0.07, 0
2.77, 54.17, 0.71, 0
3.43, 53.8, 0.04, 0
2.69, 54.24, 0.19, 0
3.21, 62, 0.83, 0
3.51, 69.5, 0.03, 0
7.82, 115.59, 0.04, 0
7.94, 118.31, 0.07, 0
7.8, 118.29, 0.19, 0
7.78, 121.79, 0.05, 0
7.93, 129.05, 0.37, 0
F1 Chemical Shift (ppm)
F2 Chemical Shift (ppm)

HMQC

24-2-2

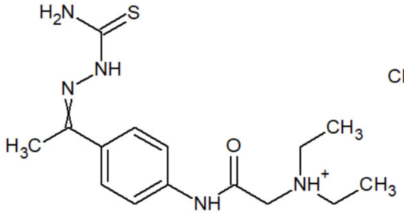

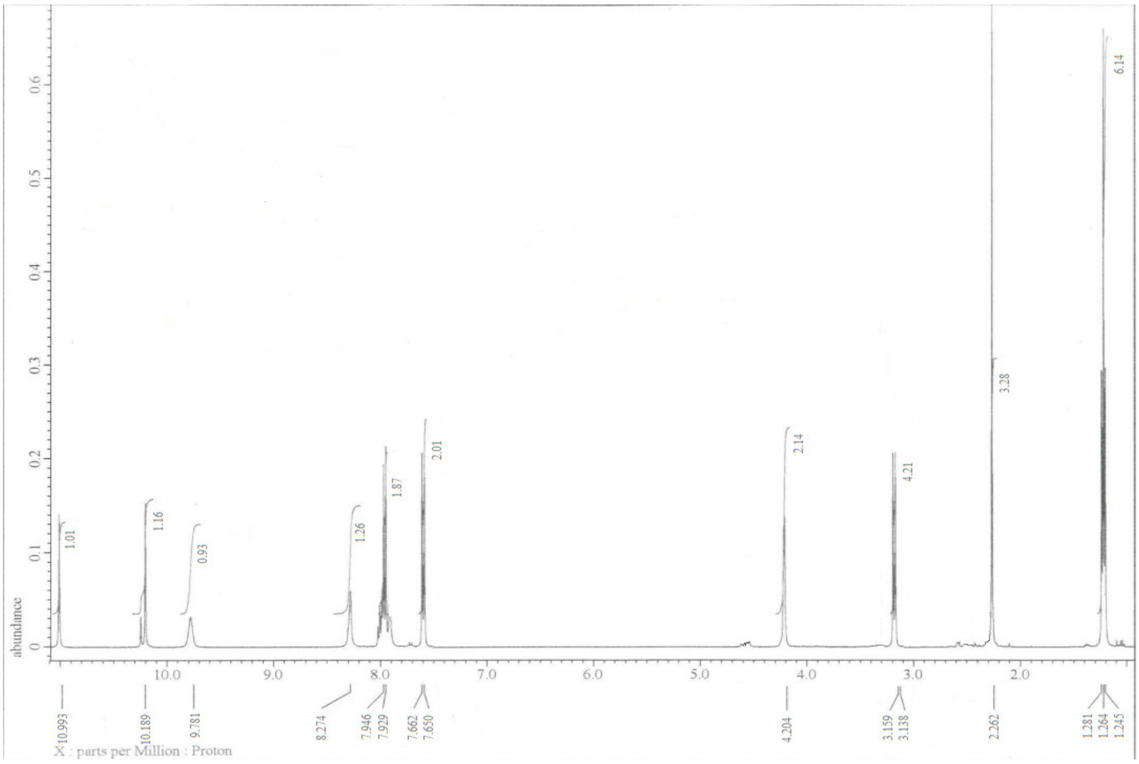

1-H NMR at 400 MHz

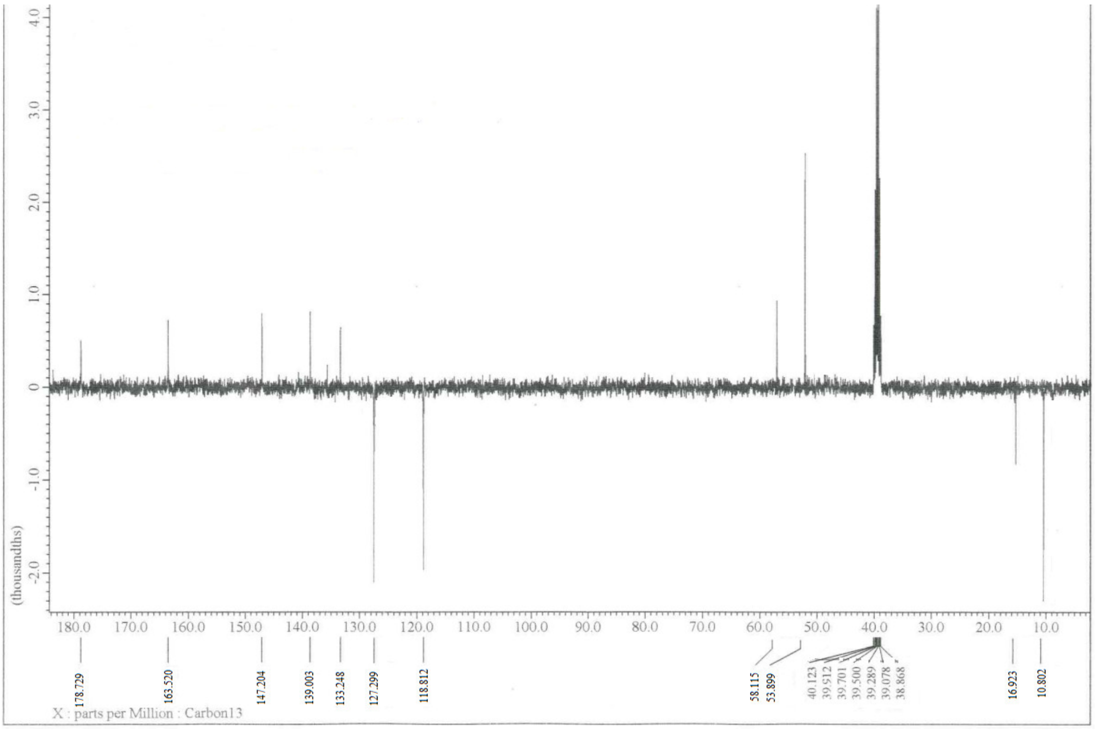

13C-apt at 100 MHz

24-11-2

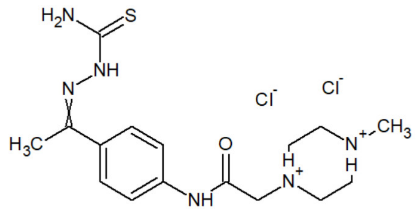

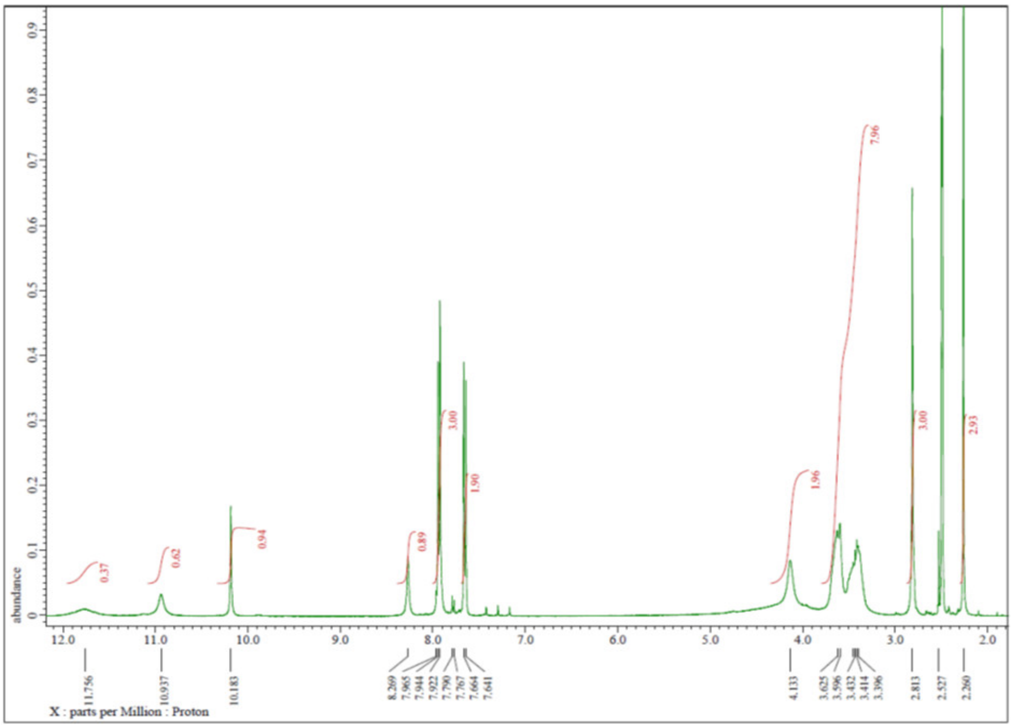

1H-NMR at 400 MHz

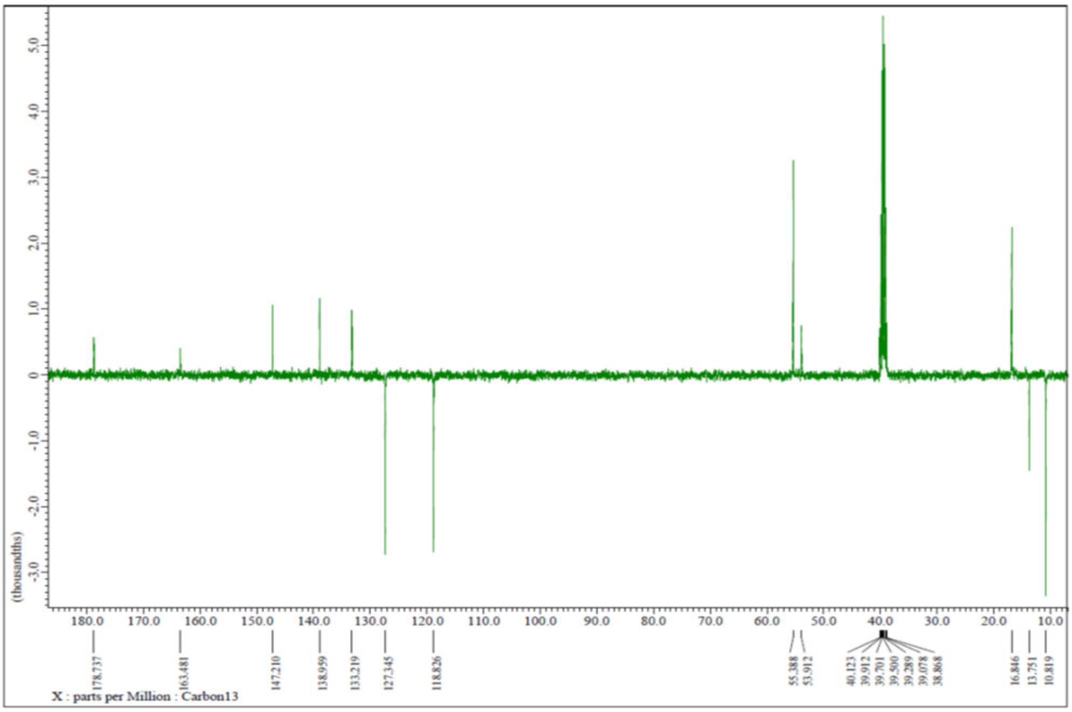

13C-apt at 100 MHz

23-7-3

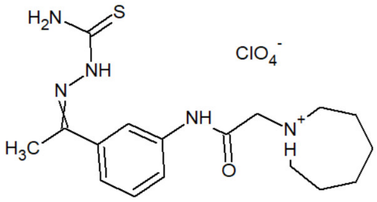

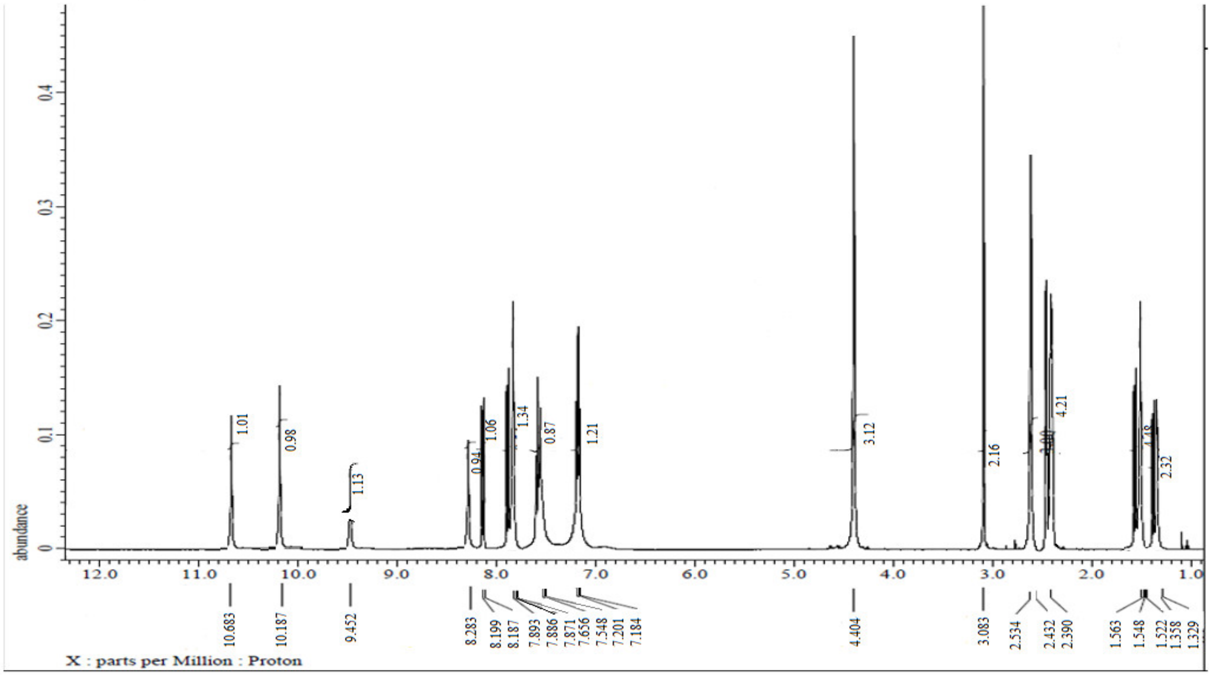

1H-NMR at 400 MHz

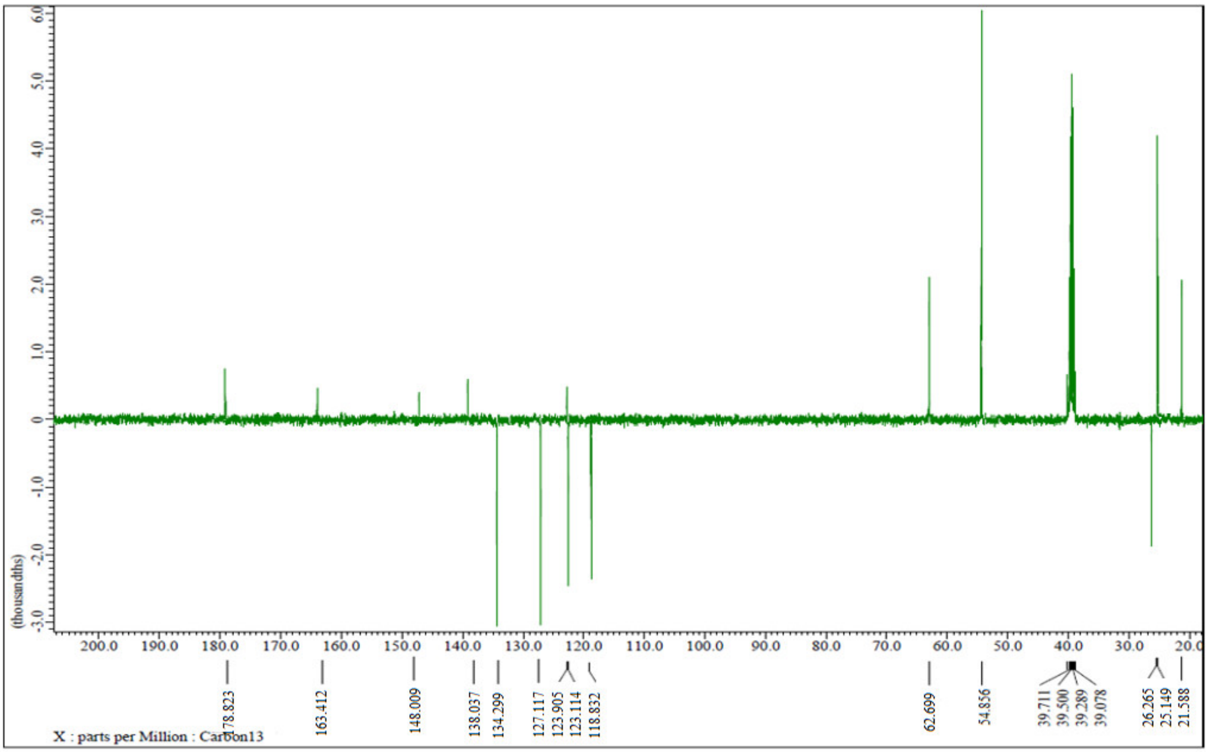

13C-apt at 100 MHz

24-2-3

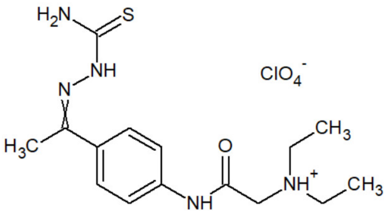

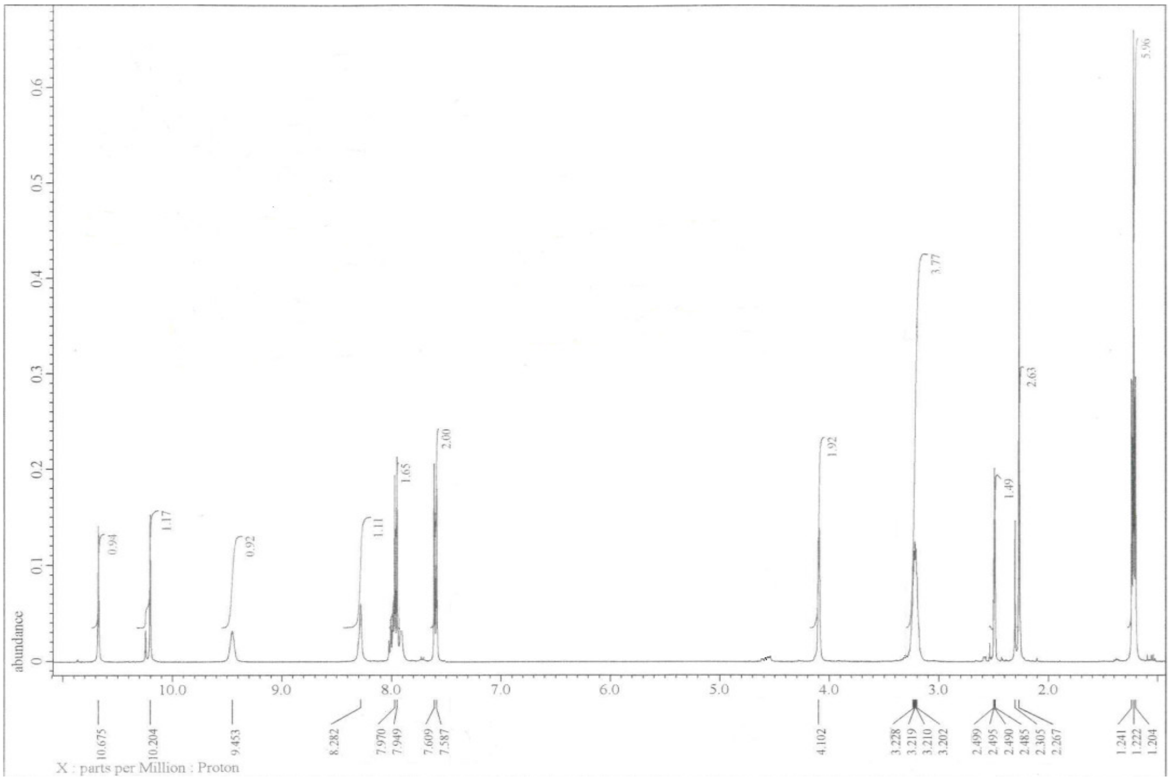

1H-NMR at 400 MHz

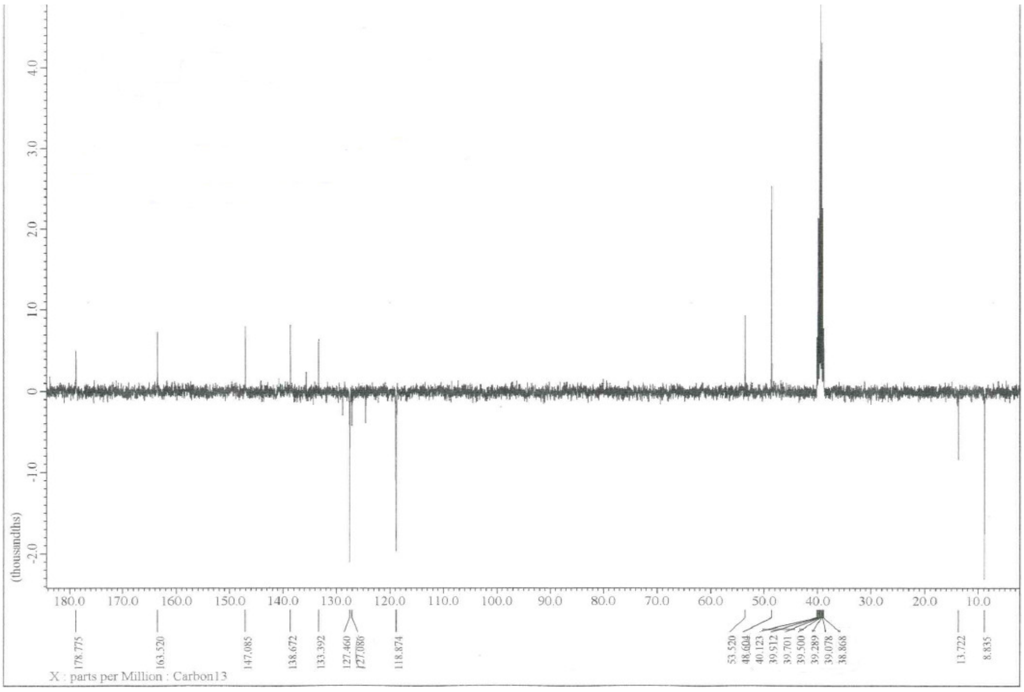

13C-apt at 100 MHz

24-5-3

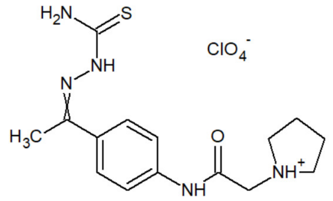

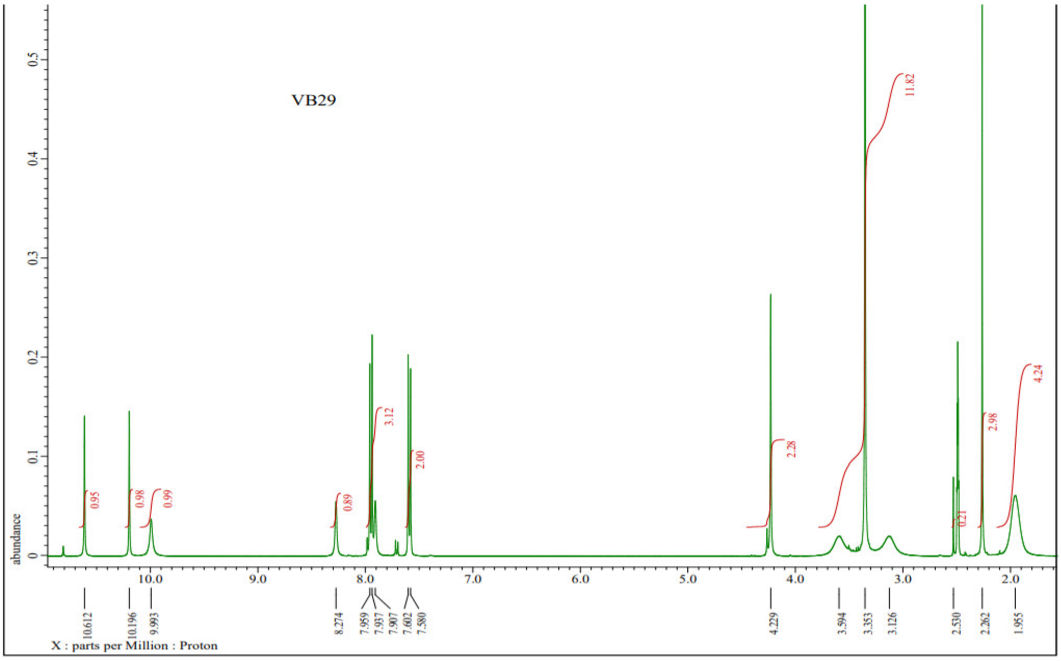

1H-NMR at 400 MHz

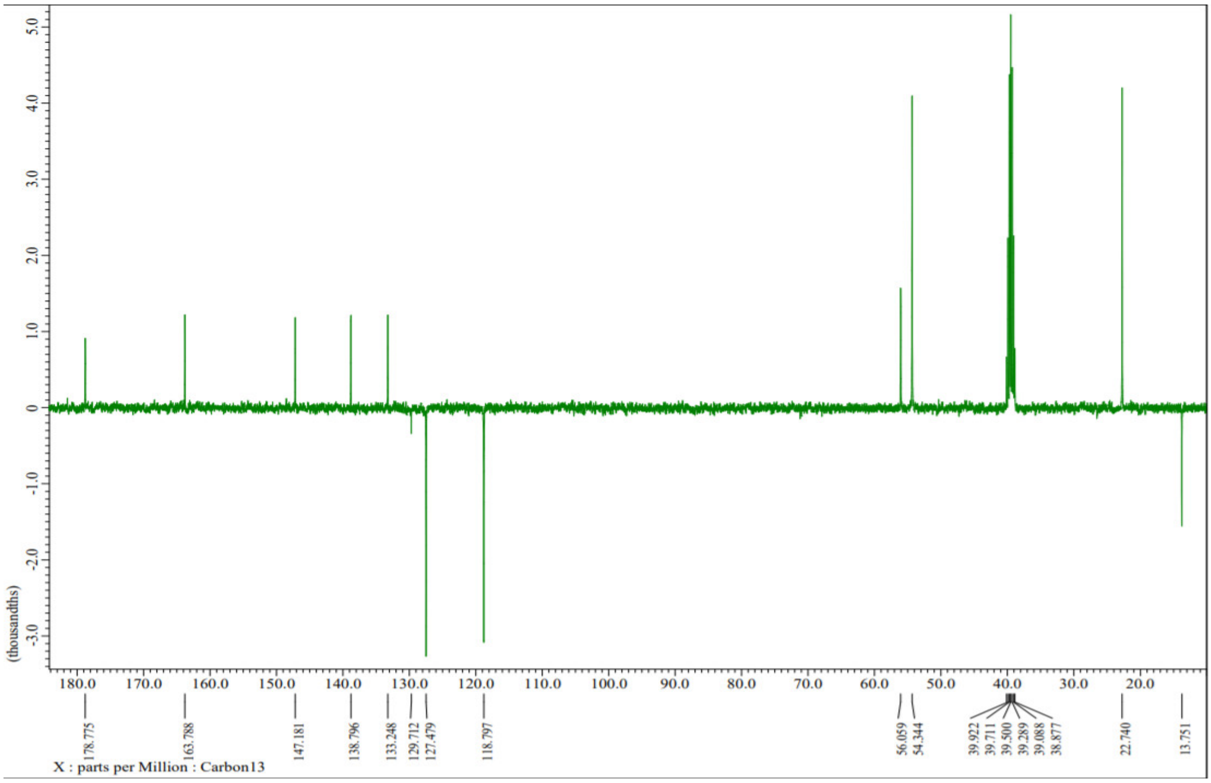

13C-apt at 100 MHz

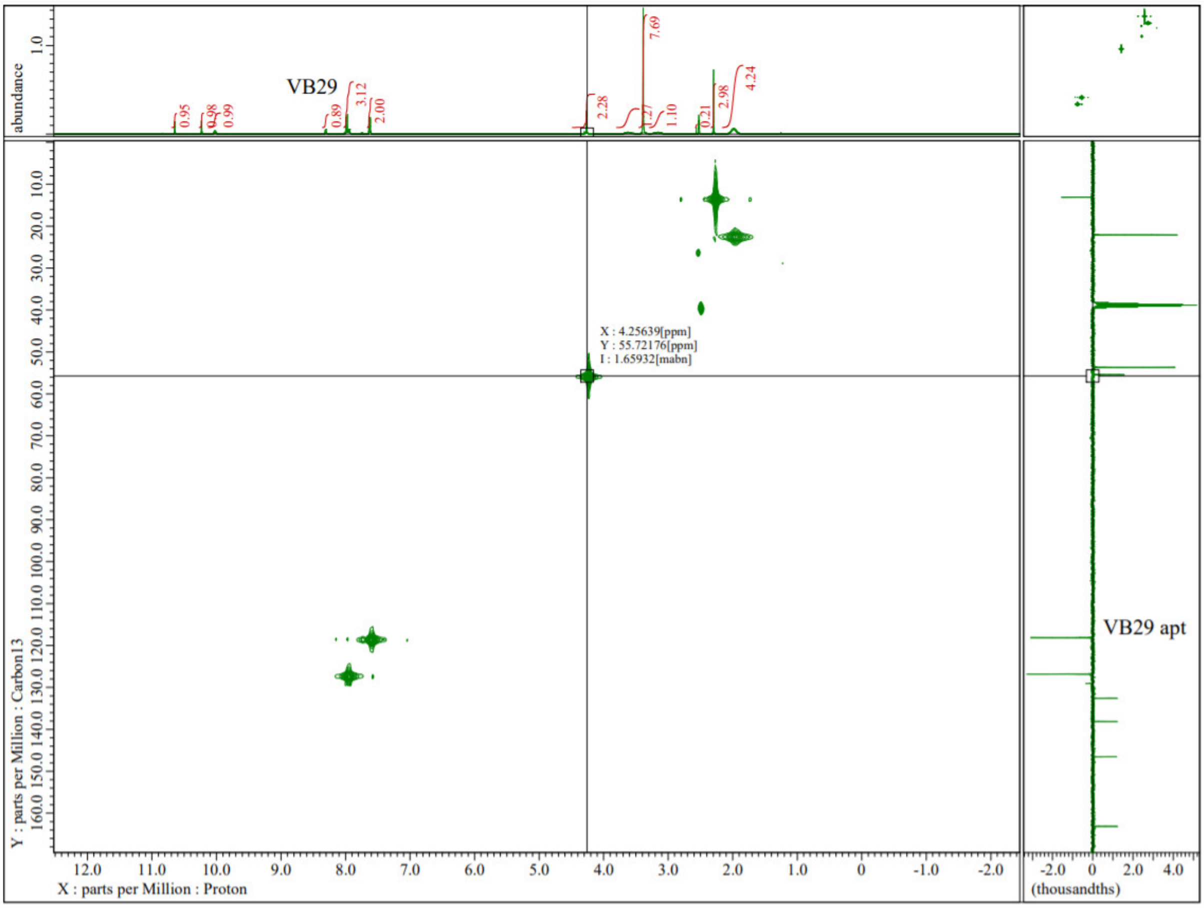

HMQC (2D; C-H direct correlation)

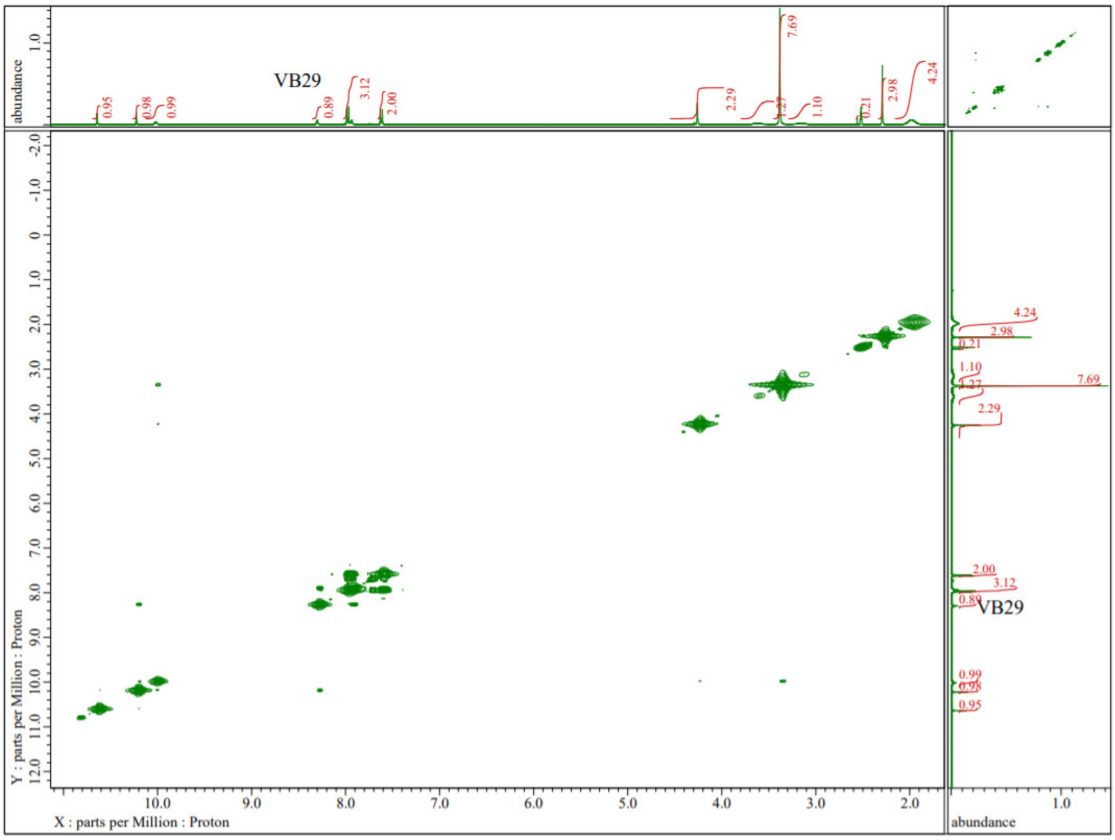

COSY

24-6-3

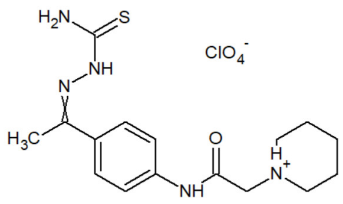

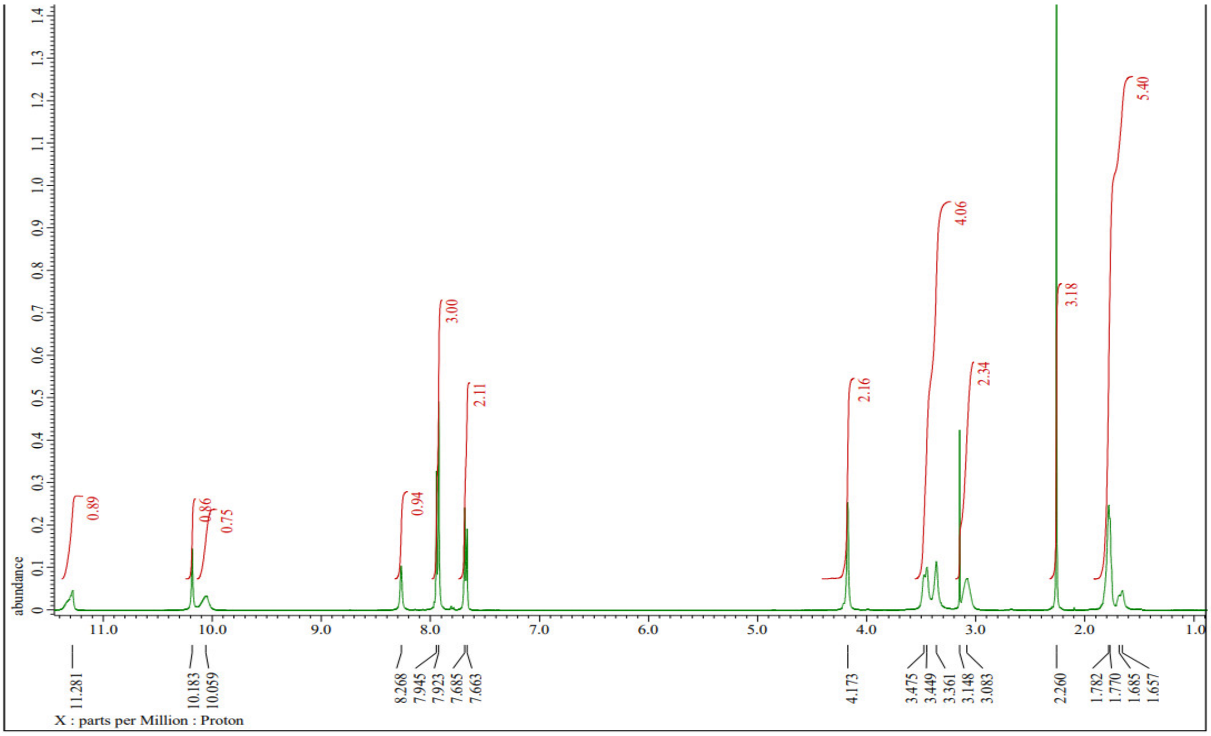

1H-NMR at 400 MHz

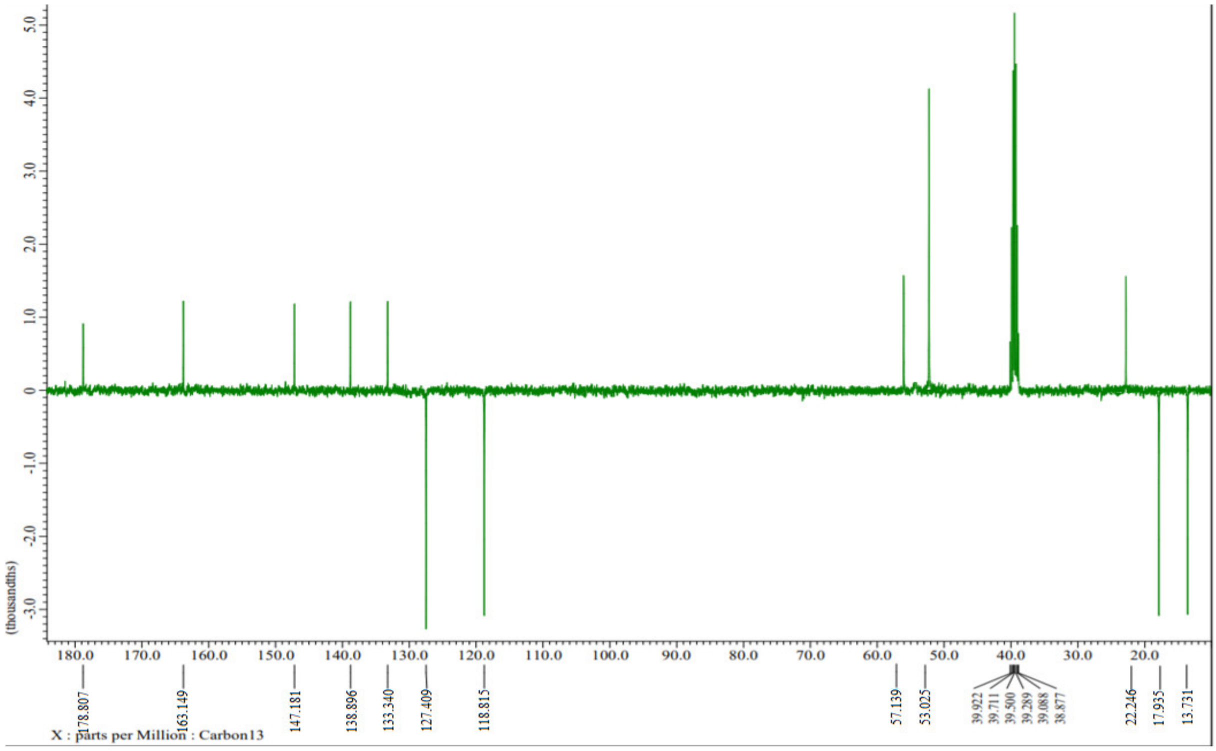

13C-apt at 100 MHz

24-10-3

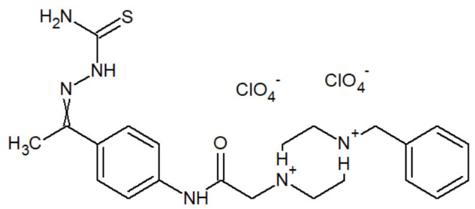

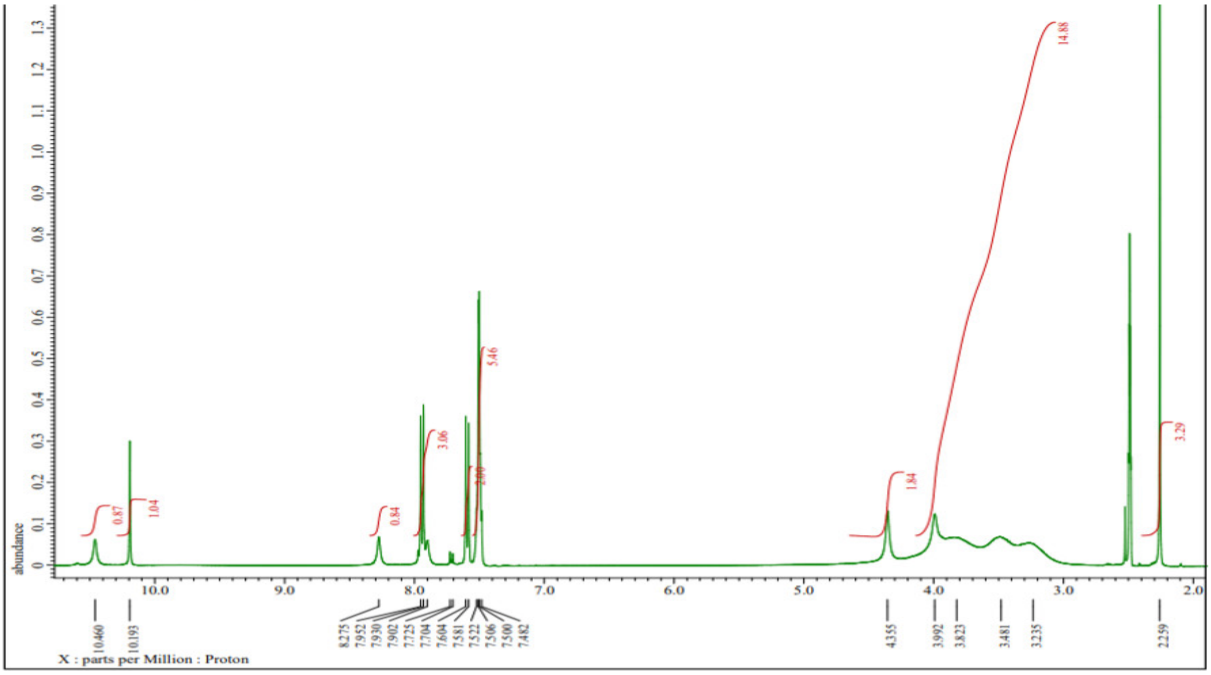

1H-NMR at 400 MHz

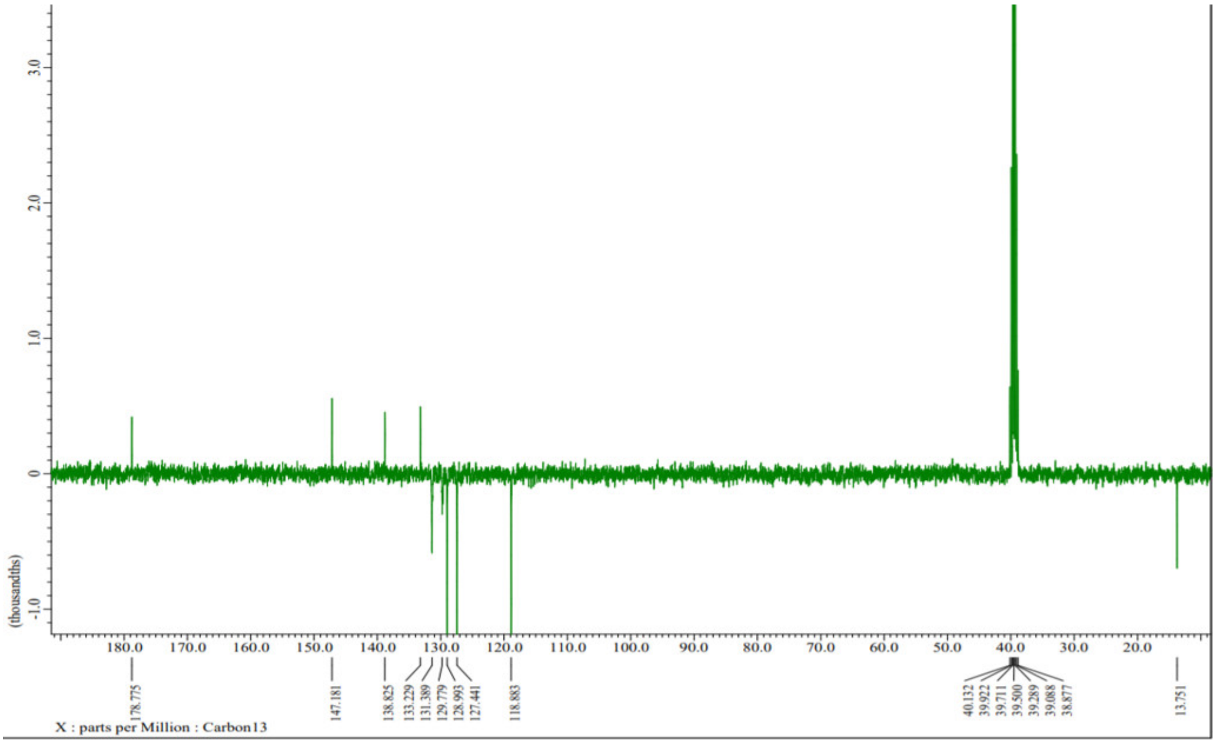

13C-apt at 100 MHz

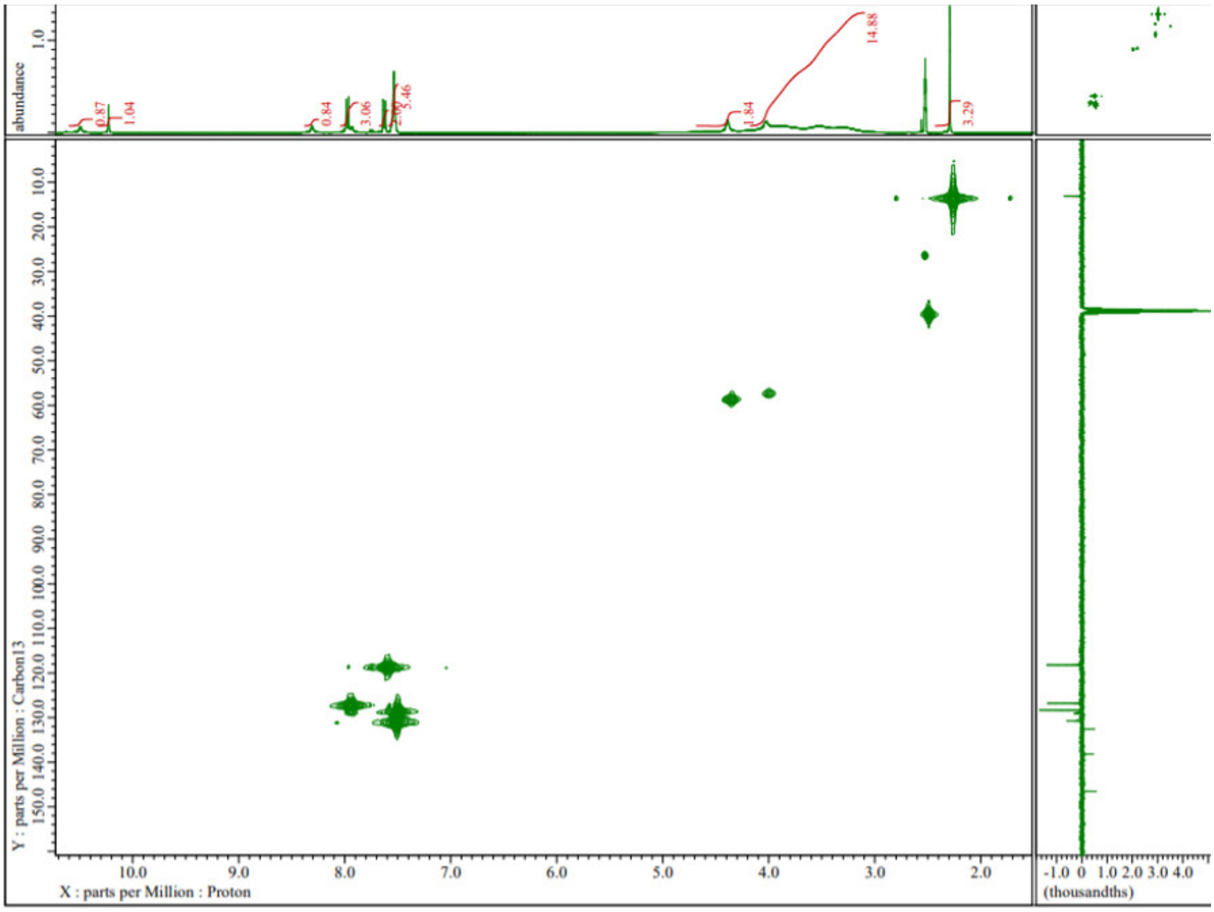
abundance
Y : parts per Million : Carbon13
X : parts per Million : Proton
(thousandths)

HMQC

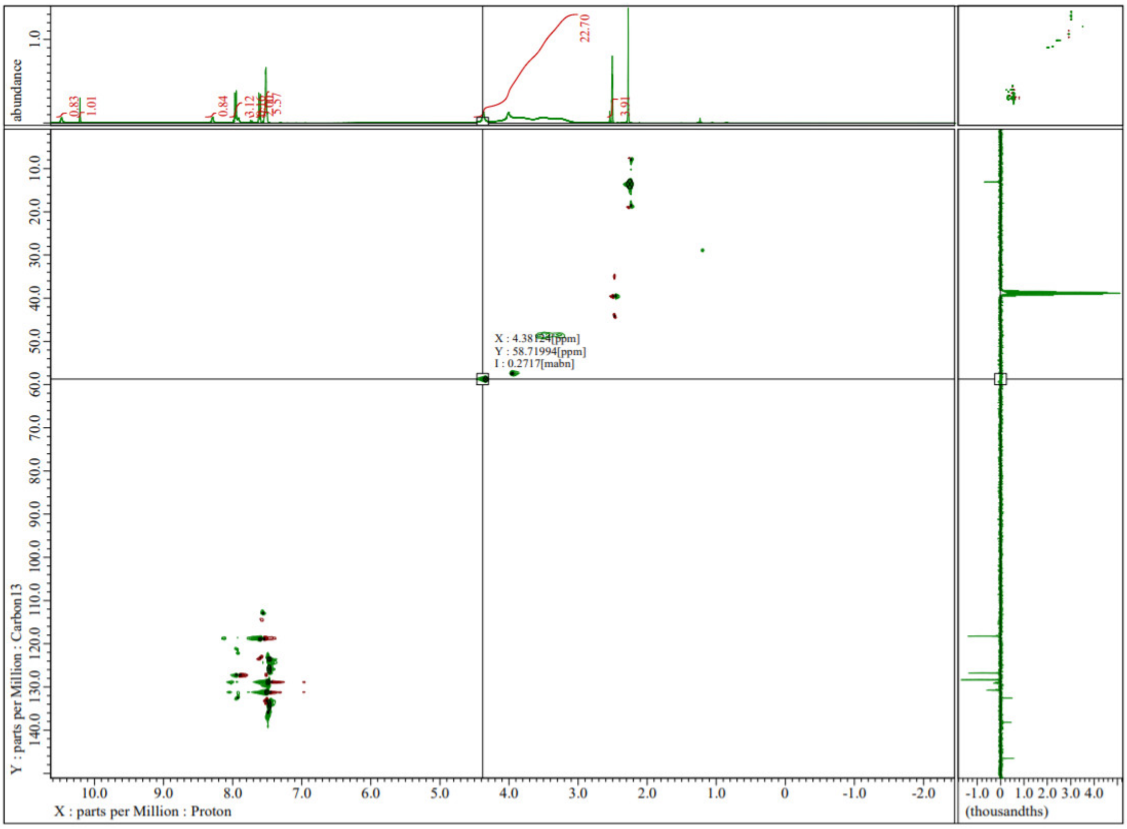
abundance
X : 4.38124[ppm]
Y : 58.71994[ppm]
I : 0.2717[mabn]
Y : parts per Million : Carbon13
X : parts per Million : Proton
(thousandths)

HSQC

24-11-3

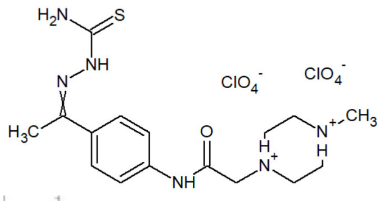

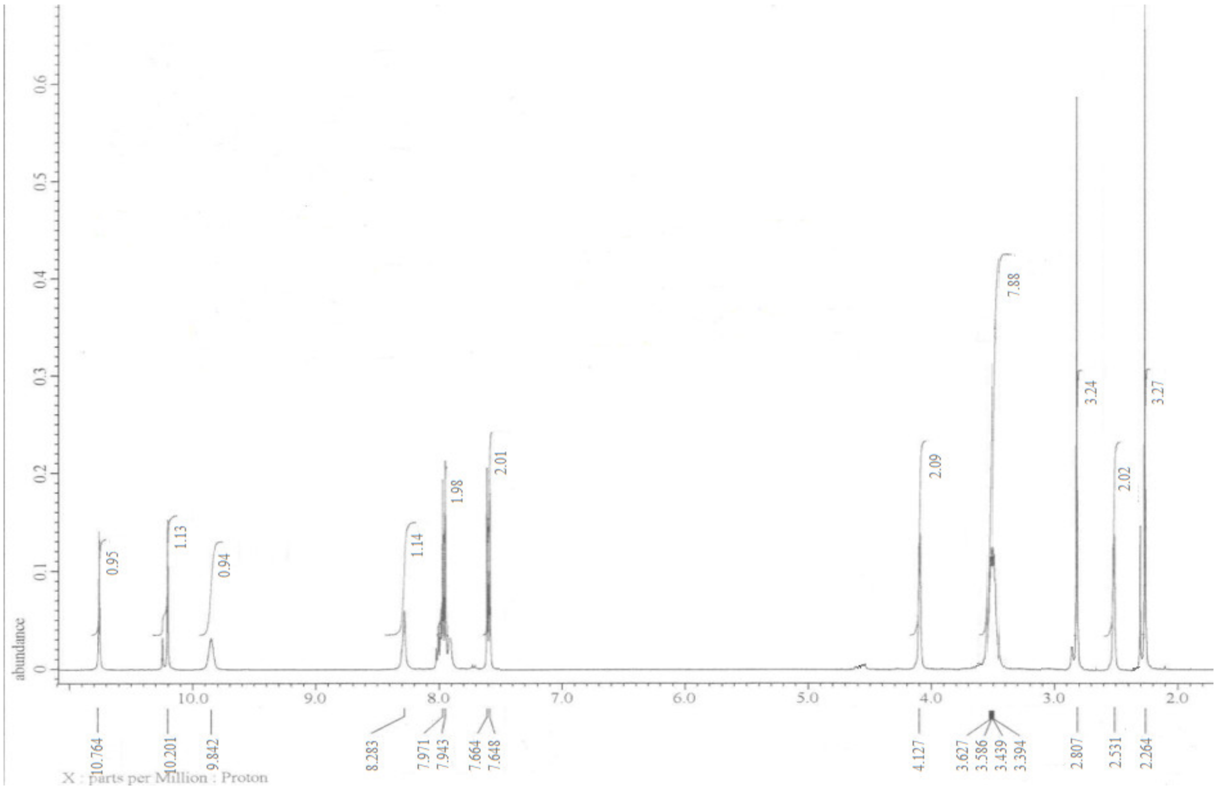

1H-NMR at 400 MHz

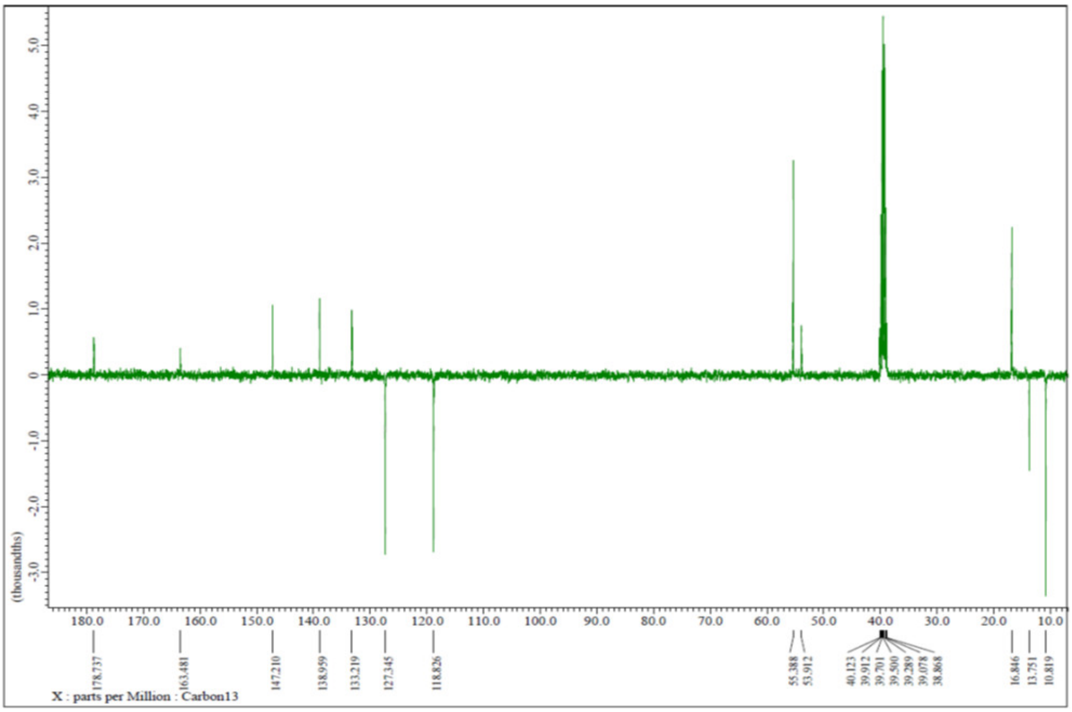

13C-apt at 100 MHz

34-2-1

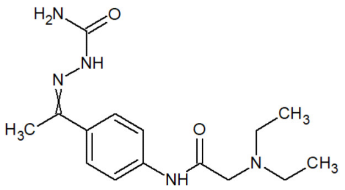

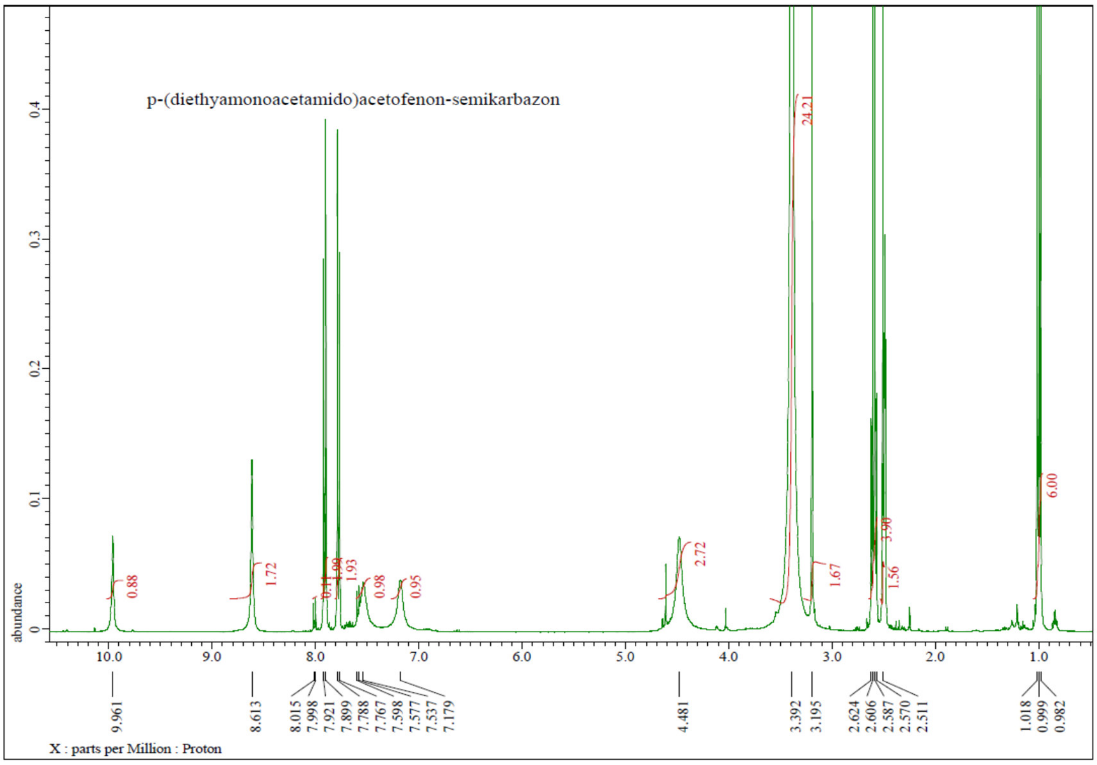

1H-NMR at 400 MHz

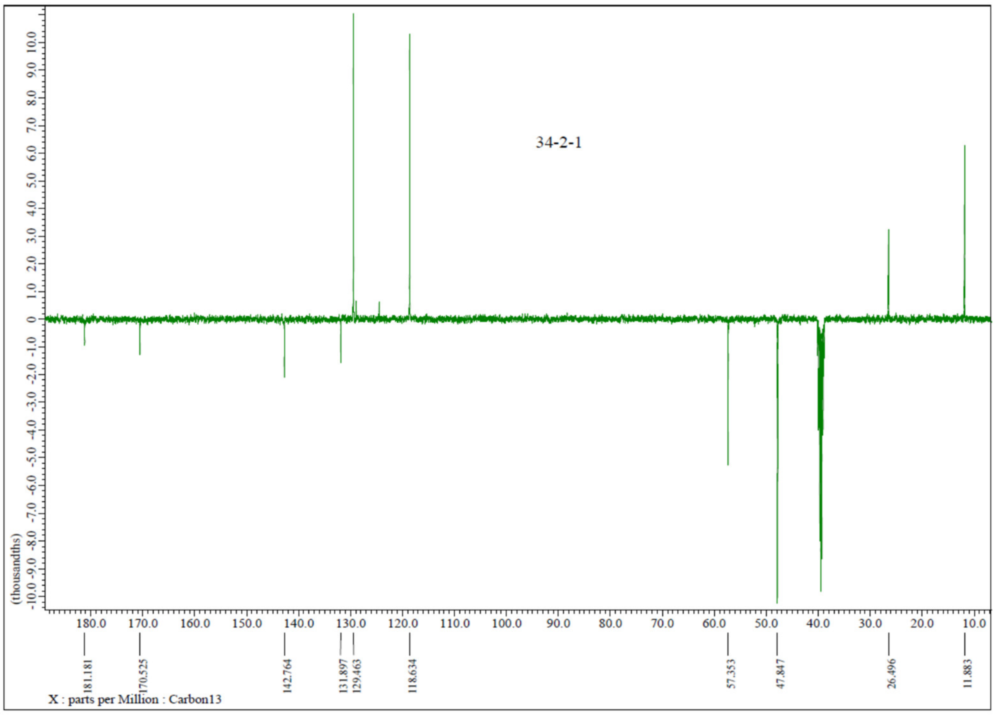

13C-apt at 100 MHz

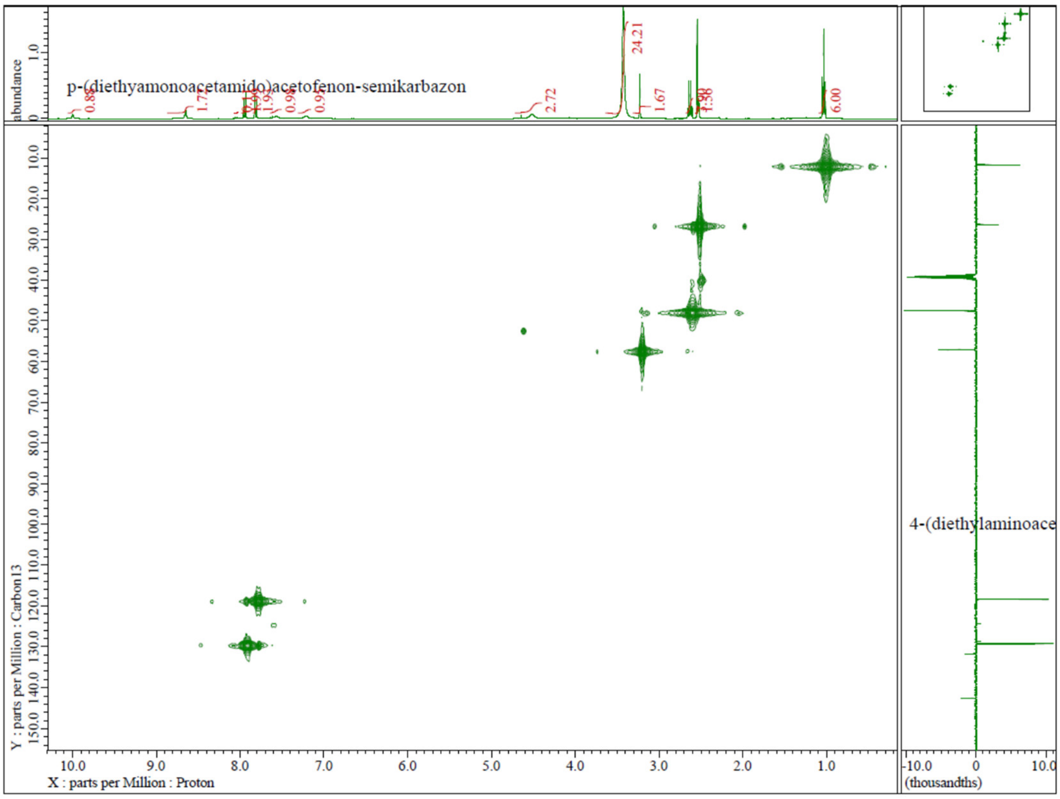

HMQC (2D; C-H direct correlation)

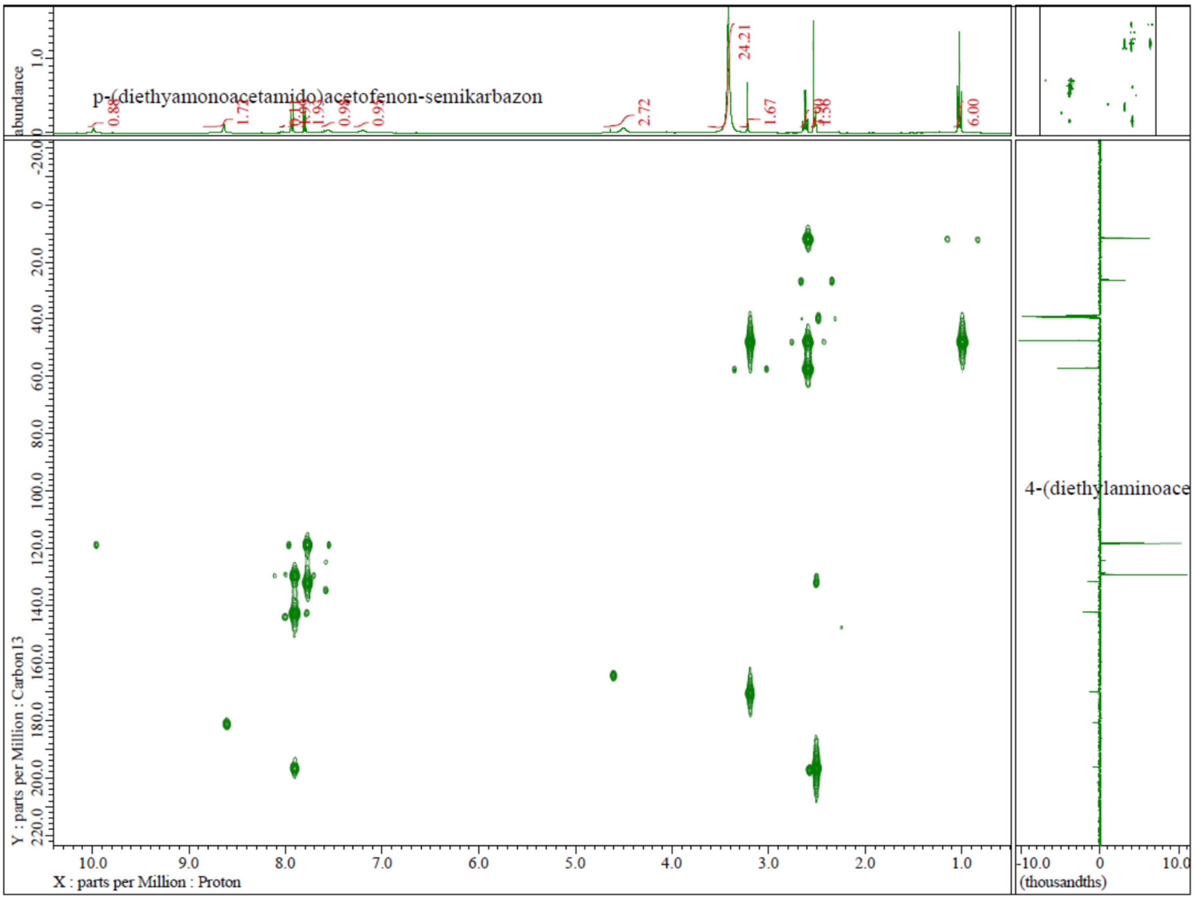

HMBC (2D; longer-range C-H correlation)

34-6-1

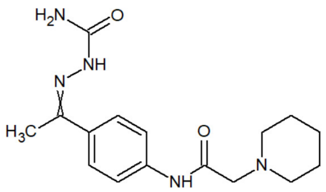

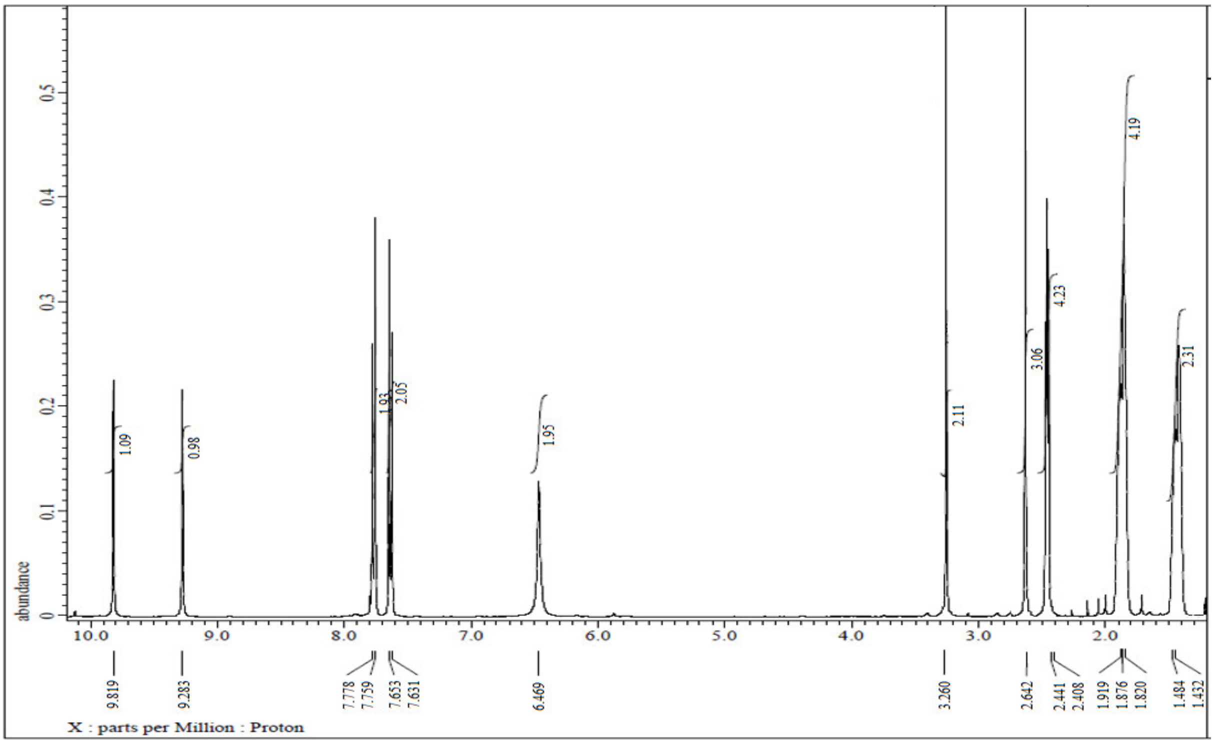

1H-NMR at 400 MHz

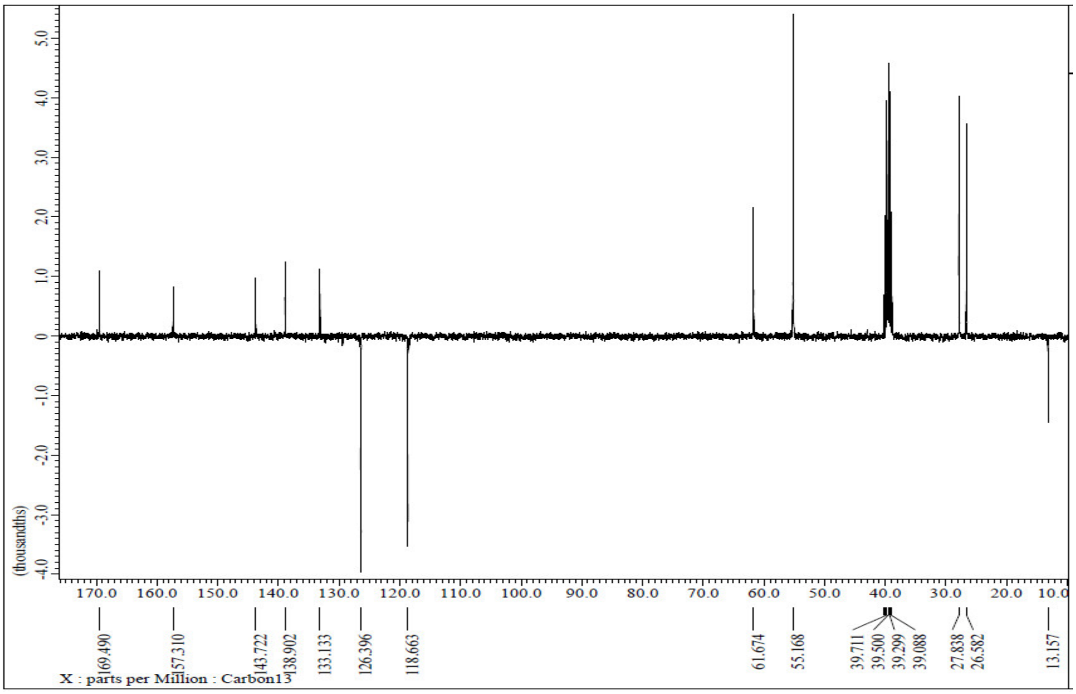

13C-apt at 100 MHz

34-7-1

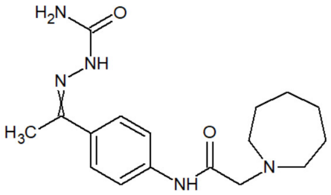

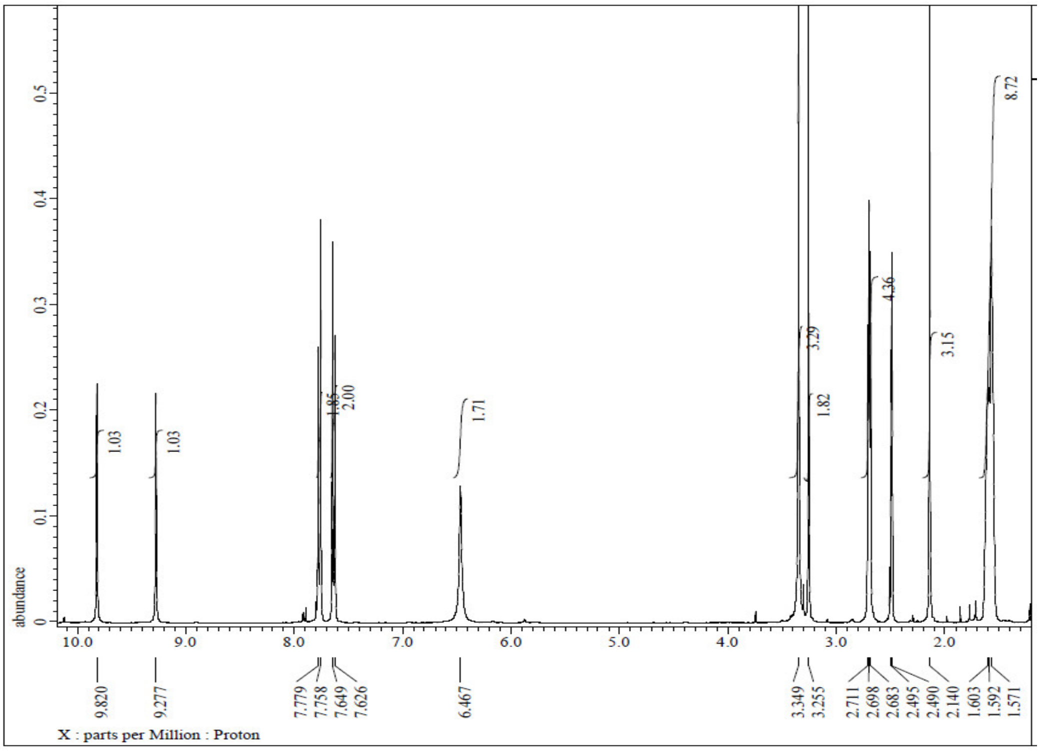

1H-NMR at 400 MHz

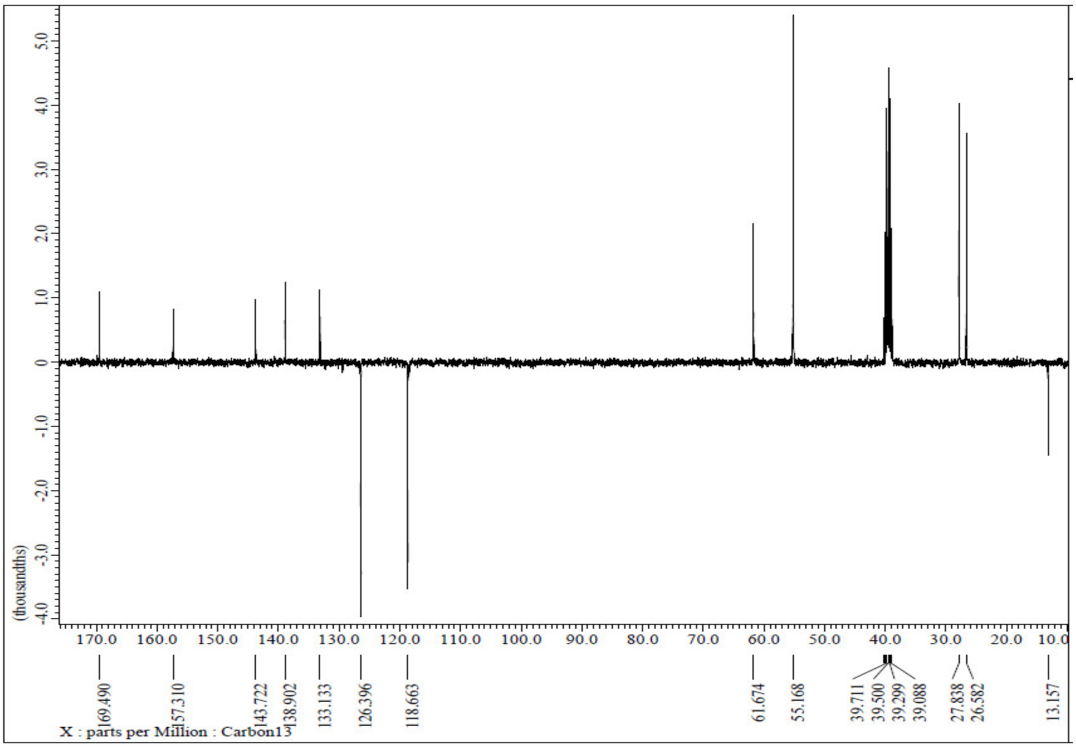

13C-apt at 100 MHz

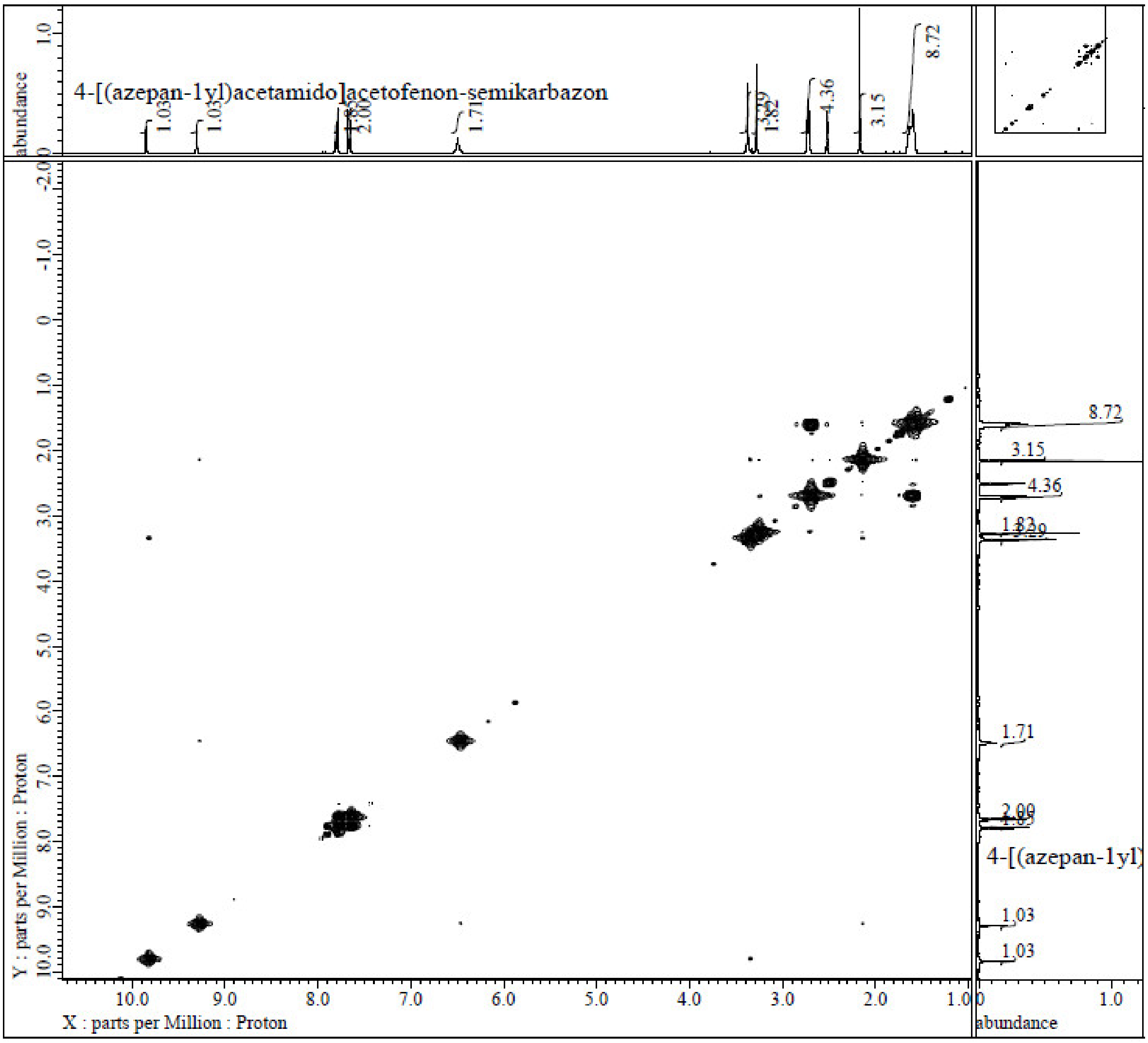
4-[(azepan-1yl)acetamido]acetofenon-semikarbazon
abundance
X : parts per Million : Proton
Y : parts per Million : Proton
8.72
3.15
4.36
1.71
1.03
1.03
2.00

COSY

34-8-1

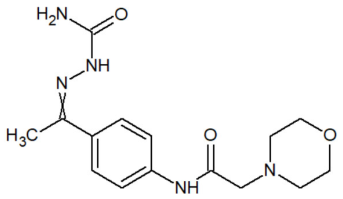

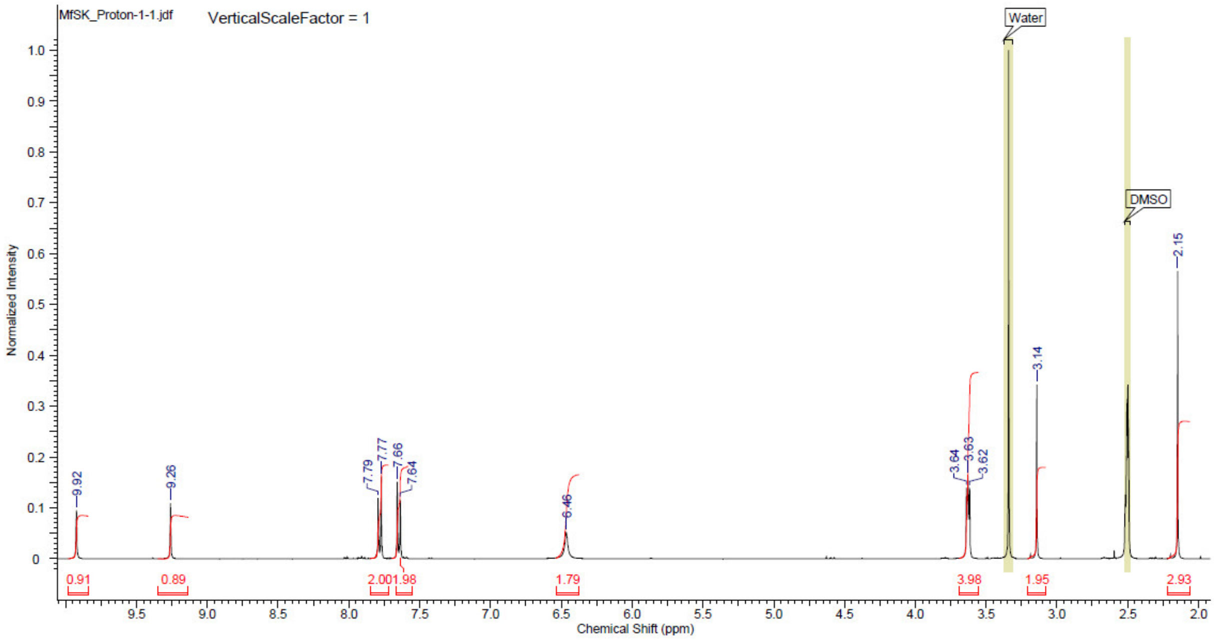

1H-NMR at 400 MHz

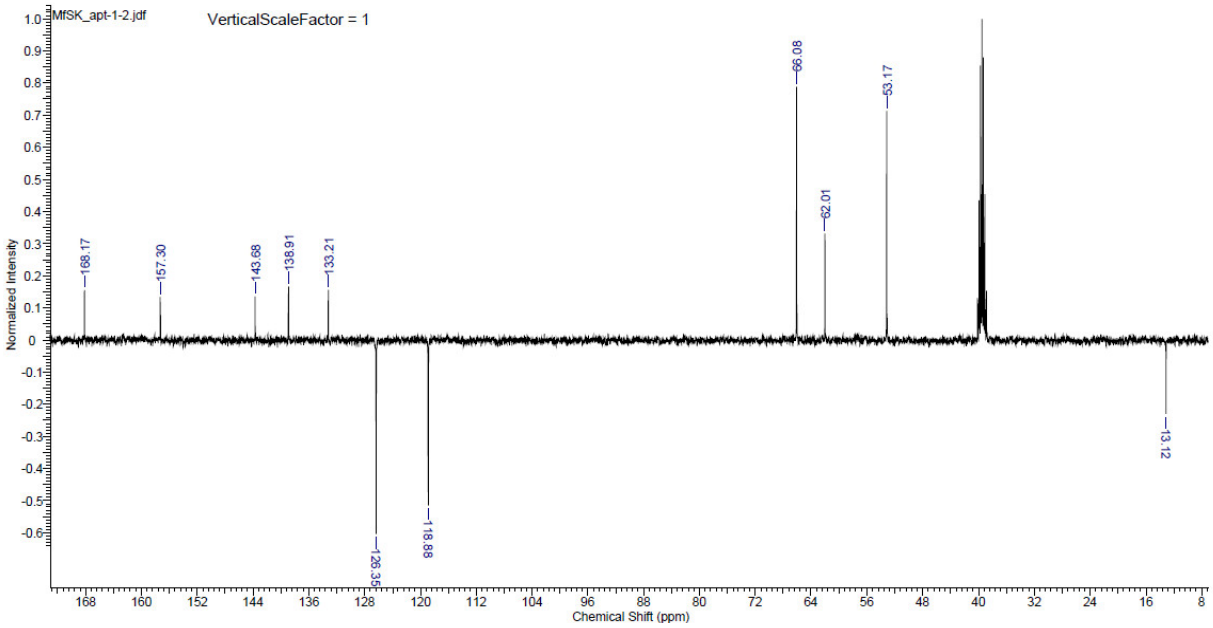

13C-apt at 100 MHz

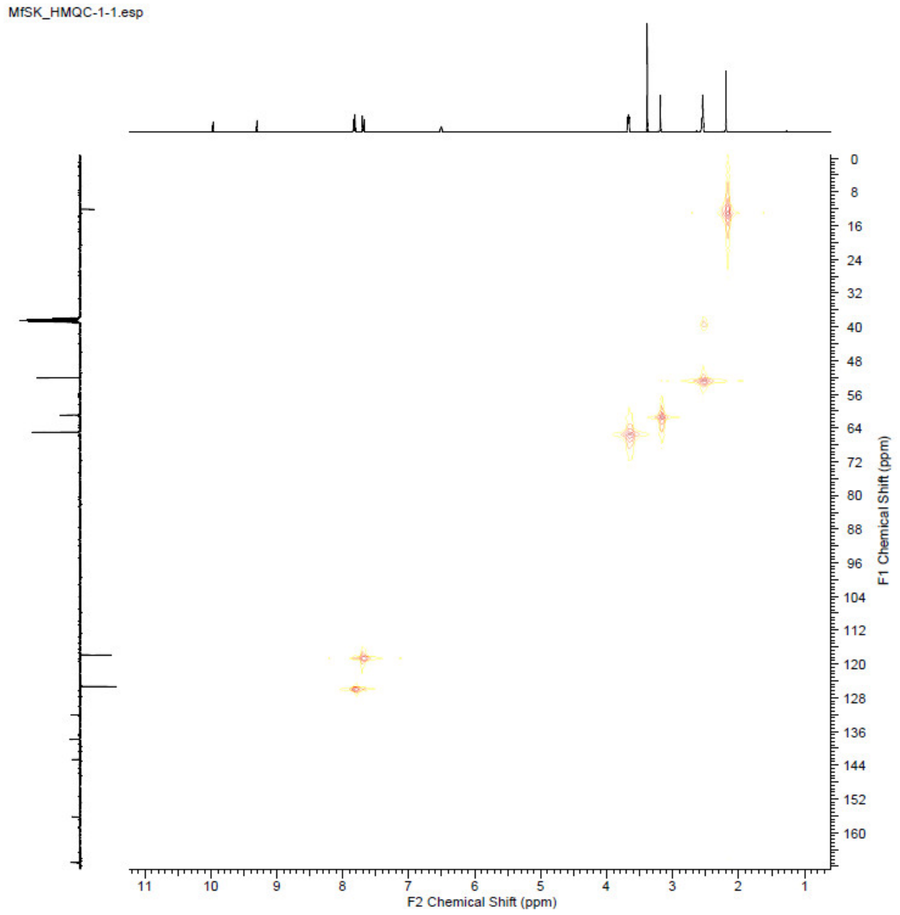
MfSK_HMQC-1-1.esp
F2 Chemical Shift (ppm)
F1 Chemical Shift (ppm)

HMQC

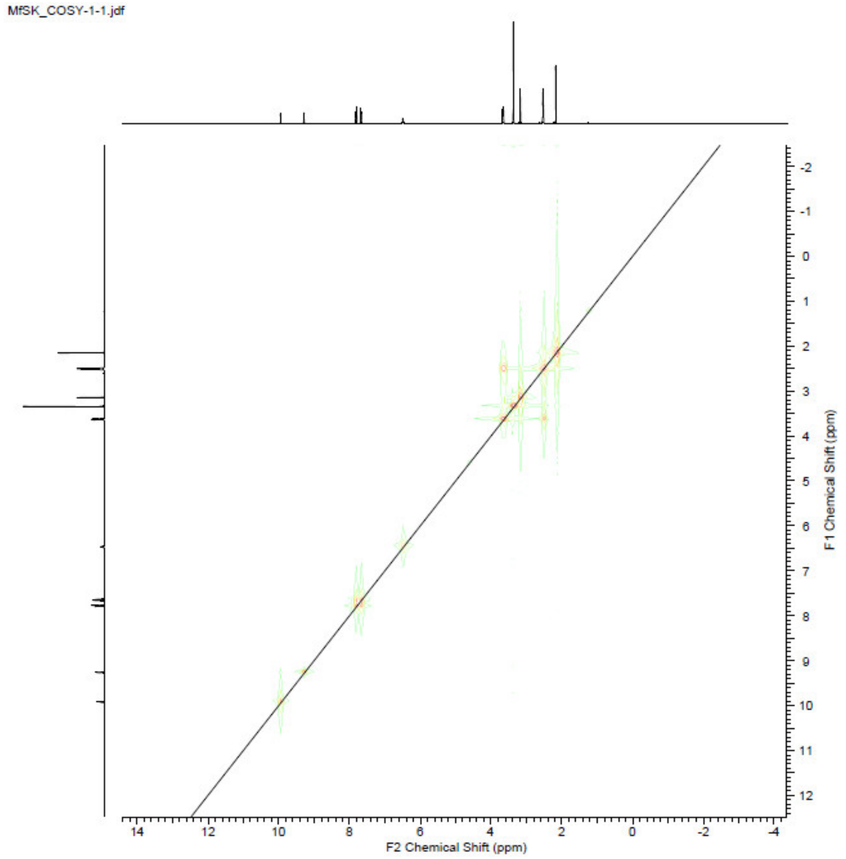
MfSK_COSY-1-1.jdf
F2 Chemical Shift (ppm)
F1 Chemical Shift (ppm)

COSY

Determination of purity of target compounds

The purity, or, more accurate, chromatography homogeneity was determined by a HPLC method using a 150 mm Kromasil C4 phase column with 100 Å porosity. Composition of the mobile phase was acetonitrile: phosphate buffer pH = 7.4 45 : 55, the flow rate 0.62 ml/s, UV detection was used at 254 nm. The retention times of all compounds were less than 10 minutes. The purity of the compound was determined as the percentage of the total area of the chromatogram that belonged to the particular compound.

Solubility issues during inhibitory activity determination and attempts to circumvent them

The determination of APN inhibitory activity of some compounds was not possible due to either their poor initial solubility in the buffer or the formation of a precipitate during their incubation with the enzyme and the substrate. We tried several known cosolvents for circumventing this problem: methanol, dimethyl sulfoxide (DMSO), pyrrolidin-2-one, 1-methylpyrrolidin-2-one (NMP), and *N*, *N*-dimethylformamide. We avoided acetone due to the eventual possibility of decomposition of our Schiff bases. Finally, we choose DMSO as the cosolvent for ketones 14-2-1 and 14-10-1 and a perchlorate 24-6-3, and NMP as the cosolvent for other poorly soluble Schiff bases. No simple dependence between lipophilicity of the compounds, and their solubility issues was found. Values of logP of compounds without solubility problems ranged between 0.17 and 3.34, values of compounds, which were possible to solubilize, between 1.6 and 2.09, and the logP value for those substances for which activity could be determined with the addition of neither DMSO nor NMP was in the range 0.89 to 2.21. For overcoming solubility issues of the remaining compounds, we also tried an HPLC protocol inspired by [23] although the HPLC approach is rare in the enzymology context. Here, a comparison of absorbance values was replaced with a comparison of areas under peaks. We expected that separation of constituents of the reaction mixture could remove problems with turbidity caused by precipitates, which disturbs absorbance measurement. Unfortunately, this approach did not bring any improvement.

Detailed results of determination of the antiproliferative activity of target compounds in different cell lines

Effect of tested compounds on cell proliferation in THP-1, MCF-7 and DU-145 cell lines after 48 h of incubation. Proliferation was determined using WST-1 assay. The results are shown as the mean ± SD of three independent experiments, each performed in triplicate. Statistical significance was calculated using a one-way analysis of variance (ANOVA) followed by Dunnett's post-test using GraphPad Prism 5.00 software. Statistical significance was assessed at levels of $p < 0.05$, $p < 0.01$ and $p < 0.001$.

**24-11-2**	**THP-1**		
**Concentration**	Proliferation of control (%)	SD (%)	Level of statistically significant

[μmol/l]			difference in comparison with drug-free control
1	98.91	0.54	--
5	98.07	0.96	--
10	96.12	2.35	--
30	88.23	3.65	p < 0.001
50	75.61	1.98	p < 0.001
75	74.67	3.67	p < 0.001
100	70.13	3.73	p < 0.001

**24-11-2**	**MCF-7**		
**Concentration [μmol/l]**	Proliferation of control (%)	SD (%)	Level of statistically significant difference in comparison with drug-free control
1	96.06	4.55	--
5	98.58	2.79	--
10	98.69	4.41	--
30	92.82	1.36	p < 0.05
50	63.78	2.70	p < 0.001
75	46.20	1.70	p < 0.001
100	44.22	1.80	p < 0.001

**24-11-2**	**DU-145**		
**Concentration [μmol/l]**	Proliferation of control (%)	SD (%)	Level of statistically significant difference in comparison with drug-free control
1	99.32	0.51	--
5	98.36	1.25	--
10	97.87	1.03	--
30	98.91	1.65	--
50	98.91	0.61	--
75	97.54	1.16	--
100	96.14	2.11	--

**34-6-1**	**THP-1**		

Concentration [μmol/l]	Proliferation of control (%)	SD (%)	Level of statistically significant difference in comparison with drug-free control
1	97.78	1.69	--
5	97.58	0.99	--
10	93.87	0.88	$p < 0.01$
30	86.64	1.73	$p < 0.001$
50	73.78	2.39	$p < 0.001$
75	71.38	1.46	$p < 0.001$
100	69.17	1.94	$p < 0.001$

34-6-1 **MCF-7**

Concentration [μmol/l]	Proliferation of control (%)	SD (%)	Level of statistically significant difference in comparison with drug-free control
1	100.09	3.23	--
5	96.80	0.48	--
10	97.62	3.21	--
30	96.30	0.52	--
50	99.57	3.94	--
75	85.41	4.03	$p < 0.001$
100	81.17	7.23	$p < 0.001$

34-6-1 **DU-145**

Concentration [μmol/l]	Proliferation of control (%)	SD (%)	Level of statistically significant difference in comparison with drug-free control
1	98.33	0.98	--
5	98.26	0.87	--
10	97.24	1.25	--
30	97.25	1.98	--
50	96.59	2.34	--
75	99.61	3.77	--
100	99.25	4.56	--

24-2-3 **THP-1**

**Concentration [μmol/l]**	Proliferation of control (%)	SD (%)	Level of statistically significant difference in comparison with drug-free control
1	98.43	0.56	--
5	96.59	1.28	--
10	91.99	2.32	$p < 0.01$
30	82.21	1.43	$p < 0.001$
50	67.65	3.13	$p < 0.001$
75	63.91	3.29	$p < 0.001$
100	62.15	3.40	$p < 0.001$

**24-2-3**	**MCF-7**		
**Concentration [μmol/l]**	Proliferation of control (%)	SD (%)	Level of statistically significant difference in comparison with drug-free control
1	97.30	1.58	--
5	97.16	2.99	--
10	87.53	0.78	$p < 0.001$
30	78.84	4.91	$p < 0.001$
50	75.70	3.96	$p < 0.001$
75	68.44	1.31	$p < 0.001$
100	60.51	1.93	$p < 0.001$

**24-2-3**	**DU-145**		
**Concentration [μmol/l]**	Proliferation of control (%)	SD (%)	Level of statistically significant difference in comparison with drug-free control
1	98.32	0.98	--
5	97.25	1.23	--
10	97.24	1.58	--
30	97.48	1.87	--
50	98.71	3.13	--
75	98.19	1.49	--
100	96.32	3.32	--

**24-10-3**	**THP-1**		

Concentration [μmol/l]	Proliferation of control (%)	SD (%)	Level of statistically significant difference in comparison with drug-free control
1	97.11	0.35	--
5	96.39	1.36	--
10	90.71	1.04	$p < 0.01$
30	80.82	4,00	$p < 0.001$
50	63.35	3.96	$p < 0.001$
75	39.81	1.11	$p < 0.001$
100	36.12	2.05	$p < 0.001$

**24-10-3**	**MCF-7**		
**Concentration [μmol/l]**	Proliferation of control (%)	SD (%)	Level of statistically significant difference in comparison with drug-free control
1	97.12	3.62	--
5	80.72	0.87	$p < 0.001$
10	66.53	1.58	$p < 0.001$
30	52.73	1.47	$p < 0.001$
50	49.99	0.87	$p < 0.001$
75	40.20	2.29	$p < 0.001$
100	34.73	1.20	$p < 0.001$

**24-10-3**	**DU-145**		
**Concentration [μmol/l]**	Proliferation of control (%)	SD (%)	Level of statistically significant difference in comparison with drug-free control
1	98.31	1.06	--
5	97.59	0.58	--
10	96.75	2.72	--
30	94.38	1.02	$p < 0.01$
50	82.57	0.60	$p < 0.001$
75	76.97	2.52	$p < 0.001$
100	69.03	2.79	$p < 0.001$

**24-5-3**	**THP-1**		

Concentration [μmol/l]	Proliferation of control (%)	SD (%)	Level of statistically significant difference in comparison with drug-free control
1	97.95	0.60	--
5	98.49	0.55	--
10	91.27	0.96	$p < 0.001$
30	73.45	0.70	$p < 0.001$
50	62.20	2.53	$p < 0.001$
75	46.36	2.62	$p < 0.001$
100	39.39	2.80	$p < 0.001$

**24-5-3**	**MCF-7**		
**Concentration [μmol/l]**	Proliferation of control (%)	SD (%)	Level of statistically significant difference in comparison with drug-free control
1	98.34	0.65	--
5	97.01	1.92	--
10	86.33	0.96	$p < 0.001$
30	75.81	1.36	$p < 0.001$
50	70.37	1.60	$p < 0.001$
75	55.54	2.63	$p < 0.001$
100	31.82	1.07	$p < 0.001$

**24-5-3**	**DU-145**		
**Concentration [μmol/l]**	Proliferation of control (%)	SD (%)	Level of statistically significant difference in comparison with drug-free control
1	99.23	0.69	--
5	98.12	1.26	--
10	97.98	2.15	--
30	97.48	1.36	--
50	97.01	0.97	--
75	97.96	0.95	--
100	96.61	2.21	--
